# PD-1 regulates latent effector differentiation of thymic cytotoxic CD8^+^ T cells

**DOI:** 10.1038/s41467-026-73392-7

**Published:** 2026-05-23

**Authors:** Zhiming Mao, Jacob B. Hirdler, Joanina K. Gicobi, Li Ding, Mark A. Maynes, Michelle A. Hsu, Emilia R. Dellacecca, Wenjing Zhang, Jacob J. Teske, Ying Li, Aubrey Y. Liew, Geoffrey Zhao, Adrian T. Ting, Virginia M. Shapiro, Fabrice Lucien-Matteoni, Henrique Borges da Silva, Daniel D. Billadeau, Haidong Dong

**Affiliations:** 1 Department of Immunology, Mayo Clinic College of Medicine and Science, Rochester, MN USA; 2 Department of Urology, Mayo Clinic, Rochester, MN USA; 3 Division of Oncology Research, Mayo Clinic, Rochester, MN USA; 4 Department of Molecular Medicine, Mayo Clinic, Rochester, MN USA; 5 Department of Quantitative Health Sciences, Mayo Clinic, Jacksonville, FL USA; 6 Department of Immunology, Mayo Clinic College of Medicine and Science, Phoenix, AZ USA

**Keywords:** Tumour immunology, Melanoma, Thymus, Cytotoxic T cells, Lymphopoiesis

## Abstract

Durable T cell immunity against cancer depends on the continual replenishment of effector CD8⁺ T cells. Thymic output has been associated with favorable prognosis in cancer patients across a range of ages, suggesting that the thymus is an important source for replenishing T cells capable of controlling cancer progression. However, whether CD8⁺ T cells acquire effector potential within the thymus, and how thymic output of effector CD8⁺ T cells contribute to peripheral tumor immunity, remain unclear. In this study, we discover that thymic single-positive (SP) CD8⁺ T cells undergo latent effector differentiation following thymic selection, but this process is subject to PD-1 regulation. We further demonstrate that PD-1 limits the contribution of thymic output of CD8⁺ T cells in shaping the TCR repertoire within the tumor tissues for tumor immunosurveillance. Although PD-1 inhibition facilitates the expansion of effector CD8⁺ T cells in the periphery, these cells gradually lose antitumor activity within tumors due to accelerated exhaustion in the absence of PD-1. Thus, while latent effector differentiation of thymic CD8⁺ T cells enables a rapid response to malignant cells in the periphery, PD-1 restrains this process to prevent overt or terminal effector differentiation, which may compromise balanced and durable peripheral immunity.

## Abstract (web summary)

Programmed cell death receptor-1 (PD-1) has been implicated in thymic regulation of T cell development and function. Here, the authors characterize CD8⁺ T cell development in PD-1–deficient mice and show that PD-1 constrains the emergence of an effector-like program during thymic development, thereby shaping peripheral T cell responses and exhaustion in tumours.

## Introduction

Durable T cell immunity against cancer depends on the continual replenishment of cytotoxic CD8^+^ T cells that have been appropriately primed and differentiated for the elimination of cancer cells^1^. Recent studies have reported continued thymic output across a range of ages in humans and a strong correlation between thymic output and favorable prognosis in cancer patients, suggesting that the thymus is an important source for replenishing T cells capable of controlling cancer progression throughout a lifetime^2,3^. Before migrating to peripheral lymphoid organs, developing T cells must receive fate-determining TCR signals and undergo thymic positive and negative selection to ensure they are safe for the host and functionally competent^4,5^. Although the proliferation and migration competence of mature T cells in the thymus has been identified^5^, it remains unclear how the effector potential of CD8^+^ T cells is regulated in the thymus before they egress to peripheral tissues to mount durable anti-tumor immunity.

The success of immune checkpoint inhibitor (ICI) therapy targeting PD-1 or PD-L1 has been associated with the generation and expansion of cytotoxic CD8^+^ T cells^6,7^. However, only a small portion of patients experience durable control of cancer progression after ICI therapy^8–10^. PD-1 inhibition is expected to revitalize effector CD8^+^ T cells from exhaustion, a functional state caused by persistent tumor antigen stimulation^11^. Current understanding is that PD-1 inhibition restores or revitalizes CD8^+^ T cells that are at a stem-like or pre-exhaustion stage, but not those that are terminally exhausted^12,13^. However, it is still not clear whether PD-1 inhibition would affect the thymic output of mature CD8^+^ T cells capable of eliminating tumor cells in the periphery.

The role of PD-1 in thymic selection has been studied in the context of T cell receptor (TCR) transgenic mice with germline PD-1 deficiency^14^. While the absence of PD-1 enhances β-selection of thymocytes, it paradoxically reduces the efficiency of positive selection^14^. This phenomenon is attributed to an increased generation of double- positive thymocytes due to enhanced β-selection in germline PD-1-deficient mice; however, these cells are not qualified for positive selection. Furthermore, transgenic mice with constitutive PD-1 overexpression on double- positive thymocytes impaired positive selection in vivo, indicating the role of PD-1 in suppressing TCR-mediated positive selections^15^. However, past research did not evaluate how PD-1 regulates the effector potential of thymic CD8^+^ T cells after positive selection.

In this study, we used a CD8 (E8I)-specific conditional *Pdcd1* knockout mouse model to investigate the impact of PD-1 deficiency following thymic positive selection. We found that PD-1 did not affect the output of positive selection but instead limited the latent effector differentiation of positively selected single positive (SP) CD8^+^ cells by suppressing the expression of the cytotoxic effector molecules NKG7 and Granzyme B. This latent effector differentiation is thymic-intrinsic, as a peripheral PD-1 blockade does not recapitulate effector differentiation in the thymus. In the periphery, PD-1-deficient CD8^+^ T cells infiltrate the tumor tissues and delay tumor growth. Notably, a subset of CD8^+^ T cell repertoire found within CD8^+^ tumor-infiltrating lymphocytes (TILs) can be traced back to thymic SP CD8^+^ T cells in the absence of PD-1, suggesting a thymic contribution of these tumor-infiltrating CD8^+^ T cell clones. Although PD-1 deficiency promotes rapid expansion of effector CD8^+^ T cells, progressive acquisition of exhaustion-associated features compromises the durability of anti-tumor immunity. Collectively, our results indicate that PD-1 restrains latent effector differentiation of thymic SP CD8^+^ T cells, thereby limiting the thymic output of effector CD8^+^ T cells for peripheral tumor immunity. Our findings show a latent effector differentiation program embedded in thymic mature CD8⁺ T cells that can be unleashed in the absence of PD-1 signals to promote tumor immunity.

## Results

### PD-1 prelimits the cytotoxic effector potential of thymic single positive CD8^+^ T cells

Cytotoxic potential is one of the key functional features of CD8^+^ T cells. In the periphery, PD-1 regulates the cytotoxic potential of CD8^+^ T cells by suppressing actin remodeling at the immunological synapse and the release of cytotoxic granules^16^. In the thymus, the absence of PD-1 leads to enhanced β-selection^14^, but how PD-1 regulates the cytotoxic potential of SP CD8^+^ T cells after thymic selection has not been clearly defined. To address this, we generated a mouse model with CD8^+^ T cell-specific *Pdcd1* knockout (CD8 E8I-Cre-*Pdcd1*^fl/fl^), hereafter referred to as CD8-Pdcd1 cKO (or Pdcd1 cKO) mice (Supplementary Fig. 1a, b), where the loss of *Pdcd1* was induced in CD8^+^ T cells after their positive selection in the thymus, which was confirmed by flow cytometry (Supplementary Fig. 1f). In this model, the absence of PD-1 did not affect the generation of SP CD8^+^ T cells based on their frequency compared to control mice (Pdcd1^fl/fl^) (Supplementary Fig. 1c–e).

**Fig. 1:**
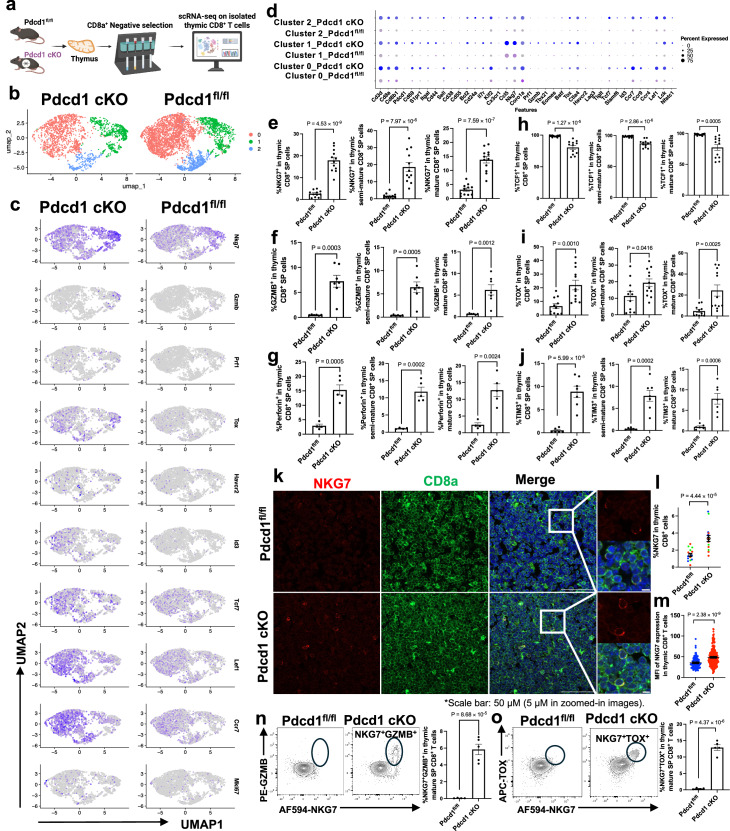
The transcriptional and protein expression of cytotoxic effector-associated genes increase in thymic SP CD8^+^ T cells in the absence of PD-1. **a**–**d** scRNA-seq analysis of thymic CD8^+^ T cells from representative control (Pdcd1^fl/fl^) and Pdcd1 cKO (*Pdcd1*^*fl/fl*^ CD8 E8I cre^+^) mice. **a** Schematic of the experimental workflow for scRNA-seq analysis of thymic SP CD8^+^ T cells. **b** UMAP plots of thymic SP CD8^+^ T cell clusters. **c** UMAP distribution and expression levels of key genes in thymic SP CD8^+^ T cells. Gene expression is depicted by a gradient, with darker blue representing higher expression levels. **d** Dotplot of featured genes expression among identified three clusters. Dot color indicates the average expression level, with darker colors indicating higher expression (blue: Pdcd1 cKO; purple: Pdcd1^fl/fl^). Dot size represents the percentage of cells expressing each gene within a cluster. **e**–**j** Flow cytometry analysis for thymic SP CD8^+^ cells (left), identifying semi-mature (TCRβ^+^CD69^+^, middle) and mature (TCRβ^+^CD69^−^, right) CD8^+^ T cell subsets. **h**–**m** Bar graphs show the frequency of NKG7 (**e** Pdcd1^fl/fl^, *n* = 11; Pdcd1 cKO, *n* = 12), Granzyme B (**f** *n* = 6 and *n* = 7), perforin (**g** *n* = 4 and *n* = 5), TCF-1 (**h** *n* = 11 and *n* = 12), TOX (**i** *n* = 11 and *n* = 12), and TIM3 (**j** *n* = 6 and *n* = 7) in each subset. **k**–**m** Immunofluorescence analysis of NKG7 protein in the thymus, with representative images of NKG7 (red) and CD8a (green) staining in (**k**). **l** Quantification of NKG7^+^ cells among thymic CD8^+^ T cells across three representative slides. **m** Quantification of median fluorescent intensity (MFI) of NKG7 in thymic CD8^+^ T cells. Frequency of GZMB^+^ (**n**) and TOX^+^ (**o**) along with NKG7^+^ in thymic SP CD8^+^ T cells (Pdcd1^fl/fl^, *n* = 4; Pdcd1 cKO, *n* = 5). In bar graphs, each point represents one mouse, with two biological replicates. scRNA-seq data represent one mouse per genotype and are shown for transcriptional profiling. Differential expression testing was performed using the Wilcoxon rank-sum test with Benjamini–Hochberg correction for multiple testing at the gene level. Each data point in (**e**–**j**) represents one mouse, with three biological replicates. For quantitative analysis shown in (**k**–**m**), multiple regions per section and multiple sections per mouse were analyzed and averaged to generate one value per mouse. Statistical comparisons were performed using mouse-level means (*n* = 3 mice per genotype). Data are presented as mean ± SEM. Statistical analyses were performed at the mouse level using unpaired two-tailed t-tests. Schematic (**a**) created using Biorender. Mao, Z. (2026) https://BioRender.com/tpqbc4u.

To examine whether PD-1 deficiency would affect the effector potential of SP CD8^+^ T cells in the thymus, we performed single-cell RNA sequencing (scRNA-seq) analysis of thymic SP CD8^+^ T cells isolated from control and CD8-Pdcd1 cKO mice (Fig. 1a). From these analyses, we identified three uniform manifold approximation and projection (UMAP) clusters (Fig. 1b). Among them, we found Cluster 1 was enriched with cytotoxic effectors genes (*Nkg7*, *Prf1*, and *Gzmb*), T cell activation-associated genes (*Cd69*, *Lck*, and *Nfatc1*), along with associated inhibitory receptors and transcriptional regulators (*Tox*, *Ctla4*, *Havcr2* [*Tim-3*], *Tigit*, and *Lag3*). Cluster 1 genes were more predominantly expressed in CD8-Pdcd1 cKO mice compared to control mice (Fig. 1c, d, and Supplementary Fig. 2a–c). We found cluster 1 genes to be enriched for multiple immune cell-signaling pathways, whereas T cell signaling pathways and protein synthesis/processing pathways were predicted to be inhibited in the absence of PD-1 (Supplementary Fig. 2d). The other two clusters (cluster 0 and cluster 2) were relatively enriched with stem-like features, characterized by upregulated expression of genes, such as *Ccr7*, *Lef1*, and *Tcf7* in the absence of PD-1 (Fig. 1d and Supplementary Fig. 2a). Our results suggest that PD-1 deficient SP CD8^+^ T cells acquire an effector-like gene profile.

Next, we performed flow cytometry analysis to validate the effector potential of thymic SP CD8^+^ T cells acquired in the absence of PD-1. We found that the frequency of NKG7^+^, GzmB^+^, and perforin^+^ SP CD8^+^ T cells increased in the thymus of CD8-Pdcd1 cKO mice compared to control mice (Fig. 1e–g). Notably, two subsets of SP CD8^+^ T cells, i.e., semi-mature (TCRβ^+^CD69^+^) and mature (TCRβ^+^CD69^−^) cells, showed increased expression of NKG7^+^, perforin^+^, and GzmB^+^ in the absence of PD-1 (Fig. 1e–g). Additionally, the transcription factors EOMES and T-Bet, which are associated with effector and memory differentiation, were also increased in SP CD8^+^ T cells in the absence of PD-1 (Supplementary Fig. 1g, h). In contrast, the stemness-associated transcription factor, TCF-1, decreased in SP CD8^+^ T cells in the absence of PD-1 (Fig. 1h). We also found T cell activation-induced transcriptional regulator TOX^17^, and the immune inhibitory receptor TIM-3 were increased in SP CD8^+^ T cells from Pdcd1 cKO mice compared to control mice (Fig. 1i, j). The acquired cytotoxic effector molecules are lineage specific, as we did not observe changes of NKG7 protein expression in thymic CD4^+^CD8^+^ double-positive and CD4^+^ SP cells (Supplementary Fig. 1i, j). To visualize the expression of NKG7 in thymic SP CD8^+^ T cells, we performed immunofluorescent staining of the thymus and found a significant increase in NKG7 expression and median fluorescent intensity in the thymic CD8^+^ T cells in the absence of PD-1 (Fig. 1k–m). From there, we also observed a positive association of NKG7 with other effector markers, such as NKG7^+^GzmB^+^, NKG7^+^TOX^+^ and NKG7^+^TIM3^+^ in thymic mature CD8^+^ T cells in the absence of PD-1 (Fig. 1n, o, and Supplementary Fig. 1k). To test whether this latent effector differentiation could be driven by TCR signals, we measured the expression of Nur77, a specific and sensitive marker of TCR signals, in thymic SP CD8^+^ T cells. We found that the frequency of Nur77^+^ cells were modestly increased in the absence of PD-1 within semi-mature T cells (Supplementary Fig. 1l). Our results suggest that latent effector differentiation may be initiated by TCR signals following positive selection but is restrained by PD-1 signaling at this stage in the thymus.

**Fig. 2:**
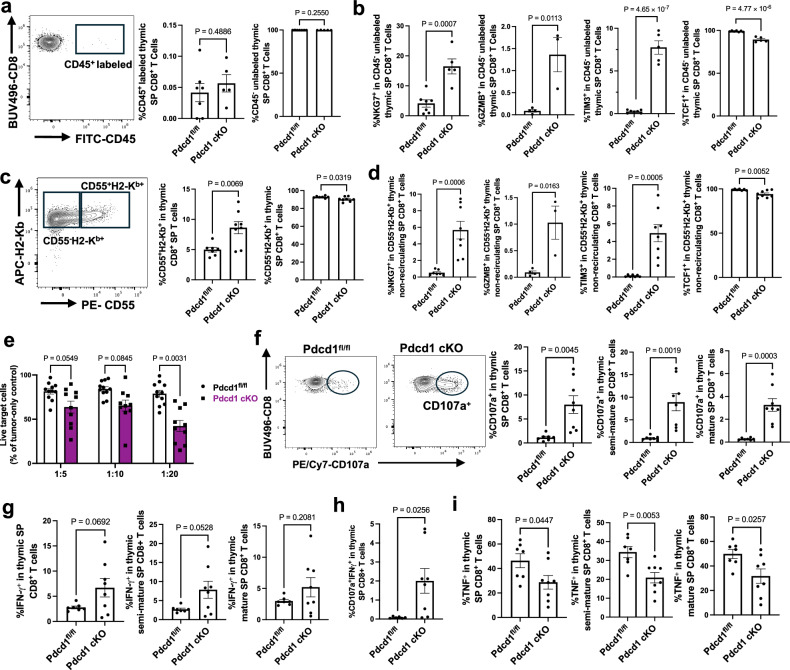
Thymic SP CD8^+^ T cells exhibit cytotoxic effector function in the absence of PD-1. **a**, **b** Distinguishing circulating from thymic resident CD8^+^ T cells via intravenous injection of anti-CD45 labeling. **a** Gating and frequency of CD45^+^ or CD45^−^ cells in thymic SP CD8^+^ T cells (Pdcd1^fl/fl^, *n* = 7; Pdcd1 cKO, *n* = 5). **b** Percentages of NKG7^+^ (Pdcd1^fl/fl^, *n* = 7; Pdcd1 cKO, *n* = 5), GZMB^+^ (*n* = 4 and *n* = 3), TIM3^+^ (*n* = 7 and *n* = 5), and TCF1^+^ (*n* = 7 and *n* = 5) cells within CD45^−^ unlabeled thymic SP CD8^+^ T cells. **c** Gating and frequency of recirculating-like CD55^+^H2-K^b+^ or non-recirculating CD55^−^H2-K^b+^ among thymic SP CD8^+^ T cells (Pdcd1^fl/fl^, *n* = 7; Pdcd1 cKO *n* = 8 mice). **d** Percentage of NKG7^+^ (*n* = 7 and *n* = 8), GZMB^+^ (*n* = 4 and *n* = 3), TIM3^+^ (*n* = 7 and *n* = 8) and TCF1^+^ (*n* = 7 and *n* = 8) within thymic non-recirculating SP CD8^+^ T cells. **e** Numbers of live target P815 cells (calculated as % of tumor-only control) after 48-h co-culture with thymic SP CD8^+^ T cells from Pdcd1^fl/fl^ (*n* = 11) and Pdcd1 cKO (*n* = 10) at indicated target-to-effector ratios. Gating and frequency of CD107a^+^ degranulation (**f**), IFN-γ^+^ (**g**), or both (**h**), and TNF^+^ (**i**) in thymic SP, semi-mature SP and mature SP CD8^+^ T cells (Pdcd1^fl/fl^, *n* = 7; Pdcd1 cKO *n* = 8). Each data point shown in this figure represents one mouse, with two to three biological replicates. Data are presented as mean ± SEM. Statistical analyses in (**e**) were performed using a mixed-effects model with Geisser-Greenhouse correction to account for repeated measures across dilution series, followed by Sidak’s multiple comparison test. Other statistical analyses were performed at the mouse level using unpaired two-tailed t-tests.

To test a potential caveat that mature T cells recirculated from the periphery might contribute to the effector phenotype of thymic SP CD8^+^ T cells, we performed 3-min intravenous labeling assay using CD45 antibody^18^, which labeled recirculating T cells but not thymic resident T cells. As expected, although CD45 antibody labeling identified the majority of peripheral CD8^+^ T cells in the blood and around 5% of splenic CD8^+^ T cells (Supplementary Fig. 3b–e), CD45⁺ cells comprised only a mean of 0.0416% of thymic SP CD8⁺ T cells in control mice and 0.0566% in CD8-Pdcd1 cKO mice, demonstrating consistently minimal recirculant contribution (Supplementary Fig. 3a and Fig. 2a). From there, we confirmed that the frequencies of NKG7^+^, GZMB^+^, TIM3^+^, and TCF1^+^ cells were increased in the CD45^-^ unlabeled thymic resident SP CD8^+^ T cells in the absence of PD-1 (Fig. 2b). Since CD55 expression has been reported to increase during T cell maturation, and mature peripheral T cells express higher CD55 compared with immature thymocytes^19,20^, we further evaluated CD55 expression as an enrichment marker for mature, recirculating-like subsets. CD55⁺H2-K^b^⁺ thymic SP CD8⁺ T cells comprised around 4.98% of the recirculating-like compartment in Pdcd1^fl/fl^ mice verses 8.62% in Pdcd1 cKO mice (Fig. 2c). However, among most thymic non-recirculating CD55^-^H2-K^b+^ SP CD8^+^ T cells, we found the frequency of NKG7, GZMB, and TIM3 increased while TCF1 decreased in the absence of PD-1 (Fig. 2d). Taken together, these results indicate that latent effector differentiation occurs in thymic resident SP CD8^+^ T cells rather than in recirculating mature CD8^+^ T cells.

### Thymic SP CD8^+^ T cells demonstrate cytotoxic effector function in the absence of PD-1

To investigate whether latent effector differentiation would produce virtual effector cells that are capable of killing tumor cells, we performed an ex vivo thymocyte-mediated cytotoxicity assay^21^, in which freshly isolated thymic CD8^+^ cells were co-cultured with P815 target cells for 48 h. Thymic CD8^+^ cells from Pdcd1 cKO mice exhibited a trend towards increased target cell killing compared with controls, which were more pronounced at higher effector-to-target ratios (Fig. 2e). In addition, we assessed degranulation and intracellular cytokine production following 4-h PMA and ionomycin stimulation of isolated thymic CD8^+^ T cells to measure T cell-intrinsic maximal effector capacity. We observed a significant increase in the frequency of CD107a^+^ degranulating cells in both thymic semi-mature and mature SP *Pdcd1*-deficient CD8^+^ T cells compared with controls (Fig. 2f). From there, the frequency of CD107a^+^IFNγ^+^ thymic CD8^+^ T cells significantly increased in Pdcd1 cKO mice (Fig. 2h), while the frequency of IFNγ^+^, TOX^+^CD107a^+^ or TCF1^−^CD107a^+^ thymic CD8^+^ T cells only marginally increased (Fig. 2g and Supplementary Fig. 1m, n). However, the frequencies of TNF^+^ thymic CD8^+^ T cells decreased in both semi-mature and mature thymic SP CD8^+^ T cells (Fig. 2i), while the percentage of IL-2^+^ CD8^+^ T cells did not change in the absence of PD-1 (Supplementary Fig. 1o). Collectively, our results suggest that latent effector differentiation generates functional cytotoxic effector-like CD8^+^ T cells in the absence of PD-1.

### Peripheral CD8^+^ T cells retained the cytotoxic effector potential post-thymic selection in the absence of PD-1

To investigate how the latent effector differentiated thymic CD8^+^ T cells would affect the peripheral T cell pool, we used CD55^+^CD24^+^ cells to identify recent thymic emigrants (RTEs). Although the frequency of RTEs in the peripheral blood was comparable between Pdcd1 cKO mice and control mice, PD-1-deficient RTEs upregulated NKG7, GZMB, and TIM-3 and downregulated TCF1 expression (Supplementary Fig. 3f). In mature peripheral CD8^+^ T cells (CD55^+^CD24^−^), which were reduced in frequency in the absence of PD-1, we likewise observed increased expression of cytotoxic effector markers and reduced TCF1 expression (Supplementary Fig. 3g). These results suggest the thymic output of effector-like CD8^+^ T cells retains key cytotoxic features in the absence of PD-1.

Along with the increased total number, but not the frequency, of splenic CD8^+^ T cells in the CD8-Pdcd1 cKO mice compared to control mice (Supplementary Fig. 4a, b), peripheral CD8^+^ T cells demonstrated increased proliferative capacity (Ki67^+^) in the absence of PD-1 (Supplementary Fig. 4c). Functionally, we found a notable increase in the frequencies of CD107a^+^ and IFNγ^+^ CD8^+^ T cells, but a decrease in TNF^+^ CD8^+^ T cells in the spleen of PD-1 cKO mice compared to control mice (Supplementary Fig. 4d–f). Accordingly, effector-like (CD44^+^CD62L^−^) CD8^+^ T cells were significantly expanded in the spleens of CD8-Pdcd1 cKO mice compared to controls (Supplementary Fig. 4g), which also expressed higher levels of cytotoxic effector molecules, including NKG7, Perforin, GZMB and EOMES, but reduced levels of TCF1 and T-bet (Supplementary Fig. 4h–p). From there, we also confirmed that the increase of cytotoxic effector molecules was only found in effector-like CD44⁺CD62L^−^ but not in naïve CD44^−^CD62L⁺ splenic CD8^+^ T cells in the absence of PD-1 (Supplementary Fig. 5a, b). Collectively, these findings suggest that PD-1 deficiency unleashes the cytotoxic and proliferative capacity of peripheral CD8⁺ T cells, driven by thymic latent effector differentiation.

To investigate whether these effector phenotypes have been programmed at transcriptional levels, we performed scRNA-seq analysis of splenic CD8^+^ T cells. Among the six transcriptionally distinct clusters (Supplementary Fig. 6a–c), all of them showed upregulated *Nkg7* expression in PD-1-deficient CD8⁺ T cells compared to control CD8^+^ T cells (Supplementary Fig. 6c–e). However, only Cluster 5 was enriched with genes encoding both cytotoxic effector and exhaustion-associated genes, such as *Nkg7*, *Prf1*, *Gzmb*, *Havcr2*, *Lag3*, and *Tigit*, along with enriched effector-associated transcription factors (*Tbx21*, *Eomes*) (Supplementary Fig. 6d, e, g). Pathway analysis revealed that cluster 5 was enriched for immune activation-related pathways, such as TCR signaling, whereas pathways associated with protein synthesis and processing were predicted to be inhibited in the absence of PD-1 (Supplementary Fig. 6f). Collectively, our results demonstrate that peripheral CD8⁺ T cells retain a transcriptional program favoring effector differentiation in the absence of PD-1.

To determine whether acute PD-1 blockade is sufficient to recapitulate the latent effector differentiation phenotype observed in Pdcd1 cKO CD8⁺ T cells, we performed side-by-side in vitro stimulation assays using naïve CD8⁺ T cells from young, age-matched Pdcd1^fl/fl^ and Pdcd1 cKO mice. To model acute anti-PD-1 blockade, these Pdcd1^fl/fl^ naive CD8⁺ T cells were stimulated with anti-CD3/CD28 in the presence of anti-PD1 antibody for 24 hours^22^. As a pharmacodynamic control, an anti-PD-1 antibody was also included in cultures of Pdcd1 cKO cells. Robust binding was confirmed to Pdcd1^fl/fl^ naive CD8⁺ T cells, but reduced binding to Pdcd1 cKO cells before and after T cell activation, confirming target engagement (Supplementary Fig. 5c, d). Early activation markers, including CD69 and S6 phosphorylation (pS6, a readout of mTOR activation), were comparable between control and *Pdcd1*-deficient naive CD8⁺ T cells in the presence of anti-PD-1 antibody, indicating similar proximal activation (Supplementary Fig. 5e, f). Although Pdcd1 cKO cells displayed increased frequencies of NKG7⁺ and TOX⁺ cells, acute PD-1 blockade of Pdcd1^fl/fl^ naïve cells did not induce a similar effector phenotype (Supplementary Fig. 5g, h).

To further investigate whether peripheral PD-1 blockade would affect thymic latent effector differentiation in vivo, we administered five doses of anti-PD-1 antibody every 2 days in naïve mice. While peripheral PD-1 blockade expanded effector cells in splenic CD8^+^ T cells, which were characterized by increased expression of NKG7, Perforin, Granzyme B, and TOX, and decreased TCF1 expression (Supplementary Fig. 4q), but PD-1 blockade did not affect the effector phenotype of thymic SP CD8⁺ T cells (Supplementary Fig. 4r). Per T cell proliferative capacity, PD-1 blockade did not affect the frequency of Ki67^+^ CD8^+^ T cells in both spleen and thymus of treated mice (Supplementary Fig. 4q, r). Since we observed Ki67^+^ CD8^+^ T cells increased in PD-1-deficient CD8^+^ T cells (Supplementary Fig. 4c), it is unexpected that PD-1 blockade did not increase T cell proliferative capacity in our model. An explanation is that cell-intrinsic PD-1 signals may mainly control T cell proliferation in the peripheral, while PD-1 ligation by its ligand may further control T cell effector function^23^. Collectively, these data indicate acute PD-1 blockade, in vitro and in vivo, is insufficient to recapitulate the latent effector differentiation observed in *Pdcd1*-deficient CD8^+^ T cells, supporting a model in which PD-1 constrains early effector imprinting.

### Latent effector differentiation of CD8^+^ T cells contributes to peripheral antitumor immunity in the absence of PD-1

**Fig. 3:**
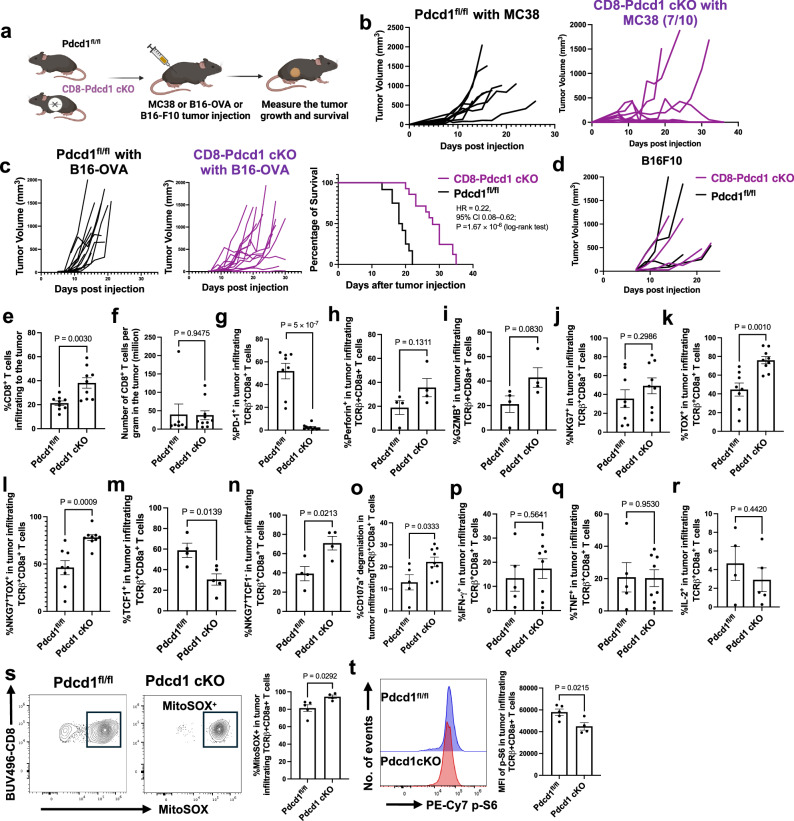
Tumor immunity is regulated by PD-1 in an immunogenicity-dependent manner. **a** Schematic of the study with tumor models. **b**–**d** Tumor growth curve of MC38 tumor (**b**, Pdcd1^fl/fl^, *n* = 8; Pdcd1 cKO *n* = 10). B16-OVA tumor **c** along with survival curve (Pdcd1^fl/fl^, *n* = 12; Pdcd1 cKO, *n* = 15), and B16-F10 tumor (**d**, Pdcd1^fl/fl^, *n* = 4; Pdcd1 cKO, *n* = 4). Percentage (**e**, *n* = 9 and *n* = 9) or absolute number (**f**, *n* = 7 and *n* = 10) of CD8^+^ TILs in Pdcd1^fl/fl^ and Pdcd1 cKO mice. Frequencies of PD-1^+^ (**g** *n* = 10 and *n* = 9), perforin^+^ (**h**, *n* = 4 and *n* = 4), GZMB^+^(**i**, *n* = 4 and *n* = 4), NKG7^+^ (**j**, *n* = 8 and *n* = 9), TOX^+^ (**k**, *n* = 8 and *n* = 9), NKG7^+^TOX^+^ (**l**, *n* = 8 and *n* = 9), TCF1^+^ (**m**, *n* = 4 and *n* = 5), NKG7^+^TCF-1^−^(**n**, *n* = 4 and *n* = 5), CD107a degranulation (**o**, *n* = 5 and *n* = 9), IFN-γ^+^ (**p**, *n* = 5 and *n* = 9), TNF^+^ (**q** *n* = 5 and *n* = 9), and IL-2^+^ (**r**, *n* = 4 and *n* = 5) in CD8^+^ TILs isolated at day 12 after B16-OVA tumor injection in Pdcd1^fl/fl^ and Pdcd1 cKO mice. **s** Gating and frequency of MitoSOX^+^ CD8^+^ TILs (Pdcd1^fl/fl^, *n* = 5; Pdcd1 cKO *n* = 4 mice). **t** Gating strategy and MFI of phosphorylated S6 (p-S6) in CD8^+^ TILs (Pdcd1^fl/fl^, *n* = 5; Pdcd1 cKO *n* = 4 mice). Each line represents one biologically independent mouse (**b**, **d**, unit of analysis: individual mouse). For MC38 and B16-F10 models, data were pooled from two independent biological experiments; for the B16-OVA model, data were pooled from four independent biological experiments. Survival curves in (**d**) were generated using the Kaplan–Meier method and compared using the log-rank (Mantel–Cox) test. Median survival were 28 days for CD8-Pdcd1 cKO mice and 18.5 days for Pdcd1^fl/fl^ mice. Hazard ratios (HR) with 95% confidence intervals (CI) are shown. Each point represents one mouse in (**e**–**t**), with two to three biological independent replicates. Data are presented as mean ± SEM. Statistical analyses were performed at the mouse level using unpaired two-tailed t-tests (**e**–**t**). **a** Created in BioRender. Mao, Z. (2026) https://BioRender.com/z190wcl.

To determine the impact of the thymic output of effector-like CD8^+^ T cells in peripheral tumor immunity, we measured and compared tumor growth between CD8-Pdcd1 cKO mice and control mice. Using three tumor models according to their degree of immunogenicity: MC38 (high), B16-OVA (intermediate), and B16F10 (low) (Fig. 3a), we found that the growth of MC38 tumors (7/10) were dramatically rejected, while the growth of B16-OVA tumors was modestly suppressed, in CD8-Pdcd1 cKO mice compared to control mice (Fig. 3b, c), while the survival of mice with B16-OVA tumors was prolonged in the absence of PD-1 (Fig. 3c). However, the tumor growth of B16F10 tumors cannot be suppressed in both CD8-Pdcd1 cKO mice and control mice (Fig. 3d). Our results suggest that PD-1 deficiency may unleash strong antitumor activity of peripheral CD8⁺ T cells, but this effect appears limited to tumors with high or intermediate immunogenicity.

To understand why the growth of tumors with intermediate immunogenicity was only delayed but not completely rejected in the absence of PD-1, we performed flow cytometry analysis of CD8^+^ T cells in TILs on day 12 post B16-OVA tumor injection, a time point with peak accumulation of effector cells^24^. PD-1 deficiency in CD8⁺ TILs (Fig. 3g) resulted in an increased frequency of CD8⁺ TILs (Fig. 3e), but not in the number of CD8⁺ TILs per gram of tumor tissue (Fig. 3f). Although PD-1-deficient CD8⁺ TILs showed modest increases in perforin⁺, GzmB⁺ and NKG7⁺ cells (Fig. 3h–j), they exhibited significant enrichment of TOX⁺, NKG7⁺TOX⁺ and NKG7⁺TCF1^−^ subsets (Fig. 3k–n). Functionally, PD-1-deficient CD8⁺ TILs retained higher cytotoxic activity (CD107a⁺) compared to controls (Fig. 3o), but did not produce more effector cytokines, such as IFNγ, TNF, or IL-2 (Fig. 3p–r). Interestingly, these cells displayed increased mitochondrial oxidative stress (MitoSOX⁺; Fig. 3s) and reduced mTORC1 activity, indicated by lower pS6 expression (Fig. 3t). Taken together, these findings suggest that while PD-1 loss expands cytotoxic effector cells and enhances antitumor activity, the durability of tumor immunity is compromised by TOX expression (a T cell exhaustion factor)^25–27^ and diminished metabolic fitness.

**Fig. 4:**
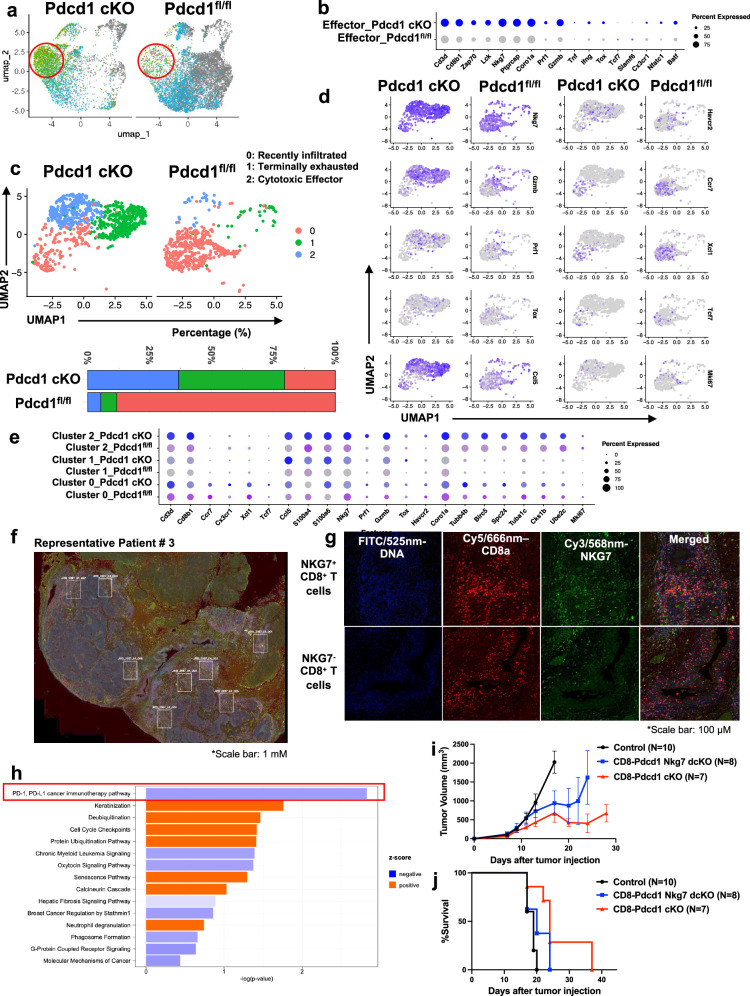
NKG7 is necessary to regulate anti-tumor immunity in the absence of PD-1. **a**–**e** Single cell RNA sequencing analysis of pooled CD8^+^ tumor infiltrating lymphocytes (TILs) isolated on day 12 post B16-OVA tumor injection in Pdcd1^fl/fl^ (*n* = 3) and Pdcd1 cKO mice (*n* = 3). **a** UMAP of pooled CD8^+^ TILs annotated using the ProjectTIL database. **b** Dot plot graph of expression of cytotoxicity-associated effector genes. **c** Three main sub-clusters within the effector CD8^+^ TIL cluster shown in (**a**). **d** UMAP distribution and expression of representative genes across the subclusters within effector CD8^+^ TILs. Gene expression is depicted by a gradient, with darker blue representing higher expression levels. **e** Dot plot graph of representative signature genes within each sub-cluster as shown in (**c**). **f**–**h** Spatial transcriptomics profiling of NKG7^+^ and NKG7^−^ CD8^+^ T cells in patients with muscle-invasive bladder cancer patients for representative regions of interests (ROIs, **f**) and representative images of NKG7^+^ or NKG7^−^CD8^+^ T cells (**g**). **h** Pathway enrichment analysis comparing NKG7⁺ versus NKG7^−^ CD8⁺ T cells from pooled four patient samples. The PD-1/PD-L1 pathway (boxed) is among the most negatively associated pathways in NKG7⁺ CD8⁺ T cells. Negative Z-scores are shown in blue; positive Z-scores in orange. Functional validation of NKG7 in regulation of PD-1 deficient CD8^+^ T cell-mediated anti-tumor immunity with tumor growth (**i**) and survival curves (**j**) of B16-OVA tumor in control mice (*n* = 10), CD8-Pdcd1 cKO (*n* = 8) and *Pdcd1*^*fl/fl*^ *Nkg7*^fl/fl^CD8 E8I cre^+^ (CD8-Pdcd1 Nkg7 dcKO, *n* = 7) mice. scRNA-seq data represent three biologically independent mice per genotype and were used for transcriptional profiling. Differential expression testing was performed using the Wilcoxon rank-sum test with Benjamini–Hochberg correction for multiple testing at the gene level. Data (**i**, **j**) were pooled from three independent biological experiments. For **i**, data are presented as mean ± SEM of the tumor volume. Survival curve (**j**) was generated using the Kaplan–Meier method and analyzed. using the log-rank (Mantel–Cox) test (*χ*²(2) = 11.55, *p* = 0.0031). Hazard ratios (HR) with 95% confidence intervals (CI) were calculated using Cox proportional hazards regression.

To determine the impact of PD-1 deficiency in the programming of antitumor activity within TILs, we performed scRNA-seq analysis of CD8^+^ TILs isolated from B16-OVA tumors. According to the published mouse ProjectTIL profile dataset^28^, we found that CD8^+^ TILs exhibited a more pronounced effector memory phenotype in CD8-Pdcd1 cKO mice compared to control mice (Fig. 4a), along with their higher expression of genes encoding cytotoxic molecules *Nkg7*, *Prf1*, and *Gzmb* (Fig. 4b and Supplementary Fig. 7e). The gene signatures of PD-1 deficient CD8^+^ TILs were associated with immune cell activation and mitochondrial metabolism pathways including TCR signaling, immunogenic cell death, respiratory electron transport and mitochondrial translation, whereas pathways related to protein synthesis and processing, similar to those in thymus and spleen, were predicted to be inhibited in the absence of PD-1 (Supplementary Fig. 7f).

To understand how CD8^+^ TILs gradually lost their antitumor activity, we further sub-clustered the CD8^+^ TILs using our scRNA-seq data (Fig. 4c and Supplementary Fig. 7g). We identified two major clusters (cluster 1 and cluster 2) in Pdcd1 cKO CD8^+^ TILs and one major cluster (cluster 0) in Pdcd1^fl/fl^ CD8^+^ TILs (Fig. 4c). Cluster 2 was characterized as “cytotoxic effector CD8^+^ T cells” with elevated expression of genes encoding cytotoxic molecules, including *Nkg7*, *Prf1*, *Gzmb*, and *Coro1a* (Fig. 4d, e). Cluster 1 was characterized as “terminally exhausted CD8^+^ T cells” with upregulated expression of *Tox* and downregulation of cytotoxic molecules, including *Nkg7*, *Prf1*, *Gzmb*, and *Coro1a* (Fig. 4d, e). Of note, cluster 2 also had a unique gene expression enriched with *Tubb4b*, *Birc5*, and *Spc24* compared to cluster 1 (Fig. 4e and Supplementary Fig. 7h). Cluster 0 was characterized as “recently infiltrated CD8^+^ T cells” (*Xcl1*, *Ccr7*, *Tafa2*, and *Tcf7*) (Fig. 4d, e). Therefore, our results suggest that the transient enhancement of antitumor activity by PD-1-deficient CD8⁺ TILs may gradually decline due to an advanced exhaustion program that is normally regulated by PD-1.

### NKG7 is an emerging functional marker of latent effector differentiation of CD8⁺ T cells for tumor immunity

In our analysis of latent effector differentiation in thymic CD8⁺ T cells and their peripheral progeny, NKG7 emerged as a key effector-associated molecule, showing upregulation at both the transcriptional and protein levels. To validate whether NKG7 expression is subject to PD-1 regulation in human tumor tissues, we performed spatial transcriptional analysis of NKG7^+^ vs. NKG7^−^ CD8^+^ TILs in four patients with muscle-invasive bladder cancer (Fig. 4f–h and Supplementary Fig. 8a, b). Among these samples, we were able to select regions of tissue enriched with NKG7^+^ CD8^+^ T cells or NKG7^−^ CD8^+^ T cells (Fig. 4g), from which we defined a differentially expressed genes that were upregulated in NKG7^+^ CD8^+^ T cells in comparison with NKG7^−^ CD8^+^ T cells (Supplementary Fig. 8c). Pathway analysis indicated that NKG7^+^ CD8^+^ T cells were enriched with genes negatively associated with the “PD-1/PD-L1 cancer immunotherapy pathway” (Fig. 4h), suggesting that the PD-1/PD-L1 pathway may regulate the expression of NKG7 in CD8^+^ TILs in human tumor tissues. To assess whether NKG7 plays a necessary role in the enhanced anti-tumor immunity in the absence of PD-1, we generated a strain of *Pdcd1*^fl/fl^*Nkg7*^flfl^ CD8 E8i Cre^+^ mice (referred to as CD8-Pdcd1 Nkg7 dcKO), in which both PD-1 and NKG7 are conditionally deleted after thymic positive selection in CD8^+^ T cells (Supplementary Fig. 8d). Using this model, we subcutaneously injected B16-OVA tumors, with CD8-Pdcd1 cKO mice and Pdcd1^fl/fl^ mice as controls. Compared with mice deficient in PD-1 alone, those lacking both PD-1 and NKG7 in CD8⁺ T cells exhibited reduced antitumor immunity, although they were still able to partially delay tumor growth compared to control mice (Fig. 4i). Accordingly, the survival of CD8-Pdcd1 Nkg7 dcKO mice was reduced compared with CD8-Pdcd1 cKO mice but remain modestly improved relative to Pdcd1^fl/fl^ mice (Fig. 4j). Given the role of NKG7 in T cell cytotoxicity and longevity^29^, our results indicate that NKG7 may serve as a key functional marker of latent effector differentiation in thymic CD8⁺ T cells and their peripheral progeny under PD-1 regulation, which is particularly important for tumor immunity.

### Latent effector differentiation of thymic CD8^+^ T cells shares TCR repertoire with peripheral TILs in the absence of PD-1

**Fig. 5:**
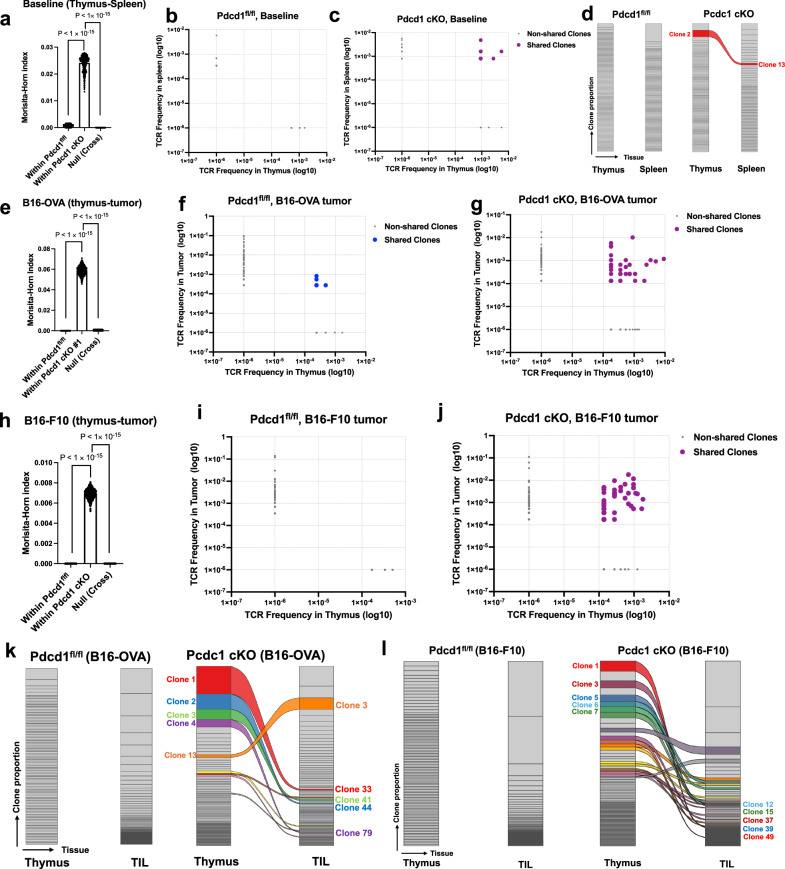
CD8^+^ T cell clonotypes are shared between the thymus and spleen or tumor tissues in the absence of PD-1. Morisita–Horn (MH) index quantified overlaps of TCR clonotypes of thymus–spleen (**a**, no tumors), thymus-B16-OVA tumors (**e**) or thymus-B16-F10 tumors (**h**), which were benchmarked against a cross-mouse null model. Clonal frequency scatter plots show productive TCR clonotype frequencies in thymus versus spleen (**b**, **c**), thymus-B16-OVA tumors (**f**, **g**), and thymus-B16-F10 tumors (**i**, **j**) from Pdcd1^fl/fl^ (**b**, **f**, **i**) and Pdcd1 cKO (**c**, **g**, **j**) mice after depth-aware normalization. Sankey diagram illustrates the selected overlapping productive TCR clonotypes between thymus and spleen in mice with no tumors (**d**), thymus-B16-OVA tumor (**k**) or thymus-B16-F10 tumors (**l**) in Pdcd1^fl/fl^ (left) and Pdcd1 cKO (right) mice. Productive repertoires were analyzed using depth-aware normalization. To control for differences in sequencing depth and clonal frequency distributions, repertoires were rarefied to a uniform read depth within each genotype–tissue comparison. For the MH index (frequency-weighted similarity), (**a**, **e**, **h**) was computed across 1000 rarefaction iterations per sample pair. Data were presented as mean ± SEM. Statistical analysis was performed using ordinary one-way ANOVA with two-sided testing, followed by Dunnett’s multiple comparisons test. Observed overlap values were benchmarked against a cross-mouse null model generated by pairing thymus and peripheral/tumor repertoires from different mice processed in parallel. Gray dots denote clonotypes detected in only one tissue; blue dots denote clonotypes detected in both tissues in Pdcd1^fl/fl^, and purple dots denote clonotypes detected in both tissues in Pdcd1 cKO. Statistical comparisons were performed on iteration-derived distributions within each biological sample pair relative to the null model; the biological unit of analysis is the mouse/sample pair, not the rarefaction iteration. For **d**, **k**, **l**, the original (unrarefied) top 100 productive clonotypes were ranked by clonal frequency within each tissue (rank 1–100). Overlapping clonotypes are color-coded to indicate identical paired TRA and TRB sequences.

To determine whether thymic SP CD8^+^ T cells undergoing latent effector differentiation directly contribute to the peripheral and tumor TCR repertoire in the absence of PD-1, we performed single-cell TCR sequencing of CD8^+^ T cells isolated from thymus, spleen and TILs. In non-tumor-bearing mice, thymus–spleen TCRs sharing was significantly increased in Pdcd1 cKO mice compared with Pdcd1^fl/fl^ controls, as reflected by both Jaccard and Morisita–Horn index, indicating enhanced overlap of both unique clonotypes and expanded clones at baseline (Supplementary Fig. 9a and Fig. 5a–d). In the B16-OVA tumor model, Pdcd1 cKO mice demonstrated increased thymus–TIL overlap of TCR clones across two independent experiments, with concordant increases in both identity-based and frequency-weighted metrics (Fig. 5e–g, k, and Supplementary Fig. 9j–m). Of note, one TCR clonotype (with TCRVβ CDR3 sequence: CASSRANYEQYF)^30^, which is specific for one known OVA antigen peptide, was only detected in the tumor but not in the thymus in one experiment (Supplementary Fig. 9m). In the less immunogenic B16-F10 model, increased thymus–tumor overlap of TCR clones was likewise observed in Pdcd1 cKO mice (Fig. 5h–j, l, and Supplementary Fig. 9c), indicating PD-1 deficiency consistently facilitates a higher thymus–tumor T cell clonal sharing during tumor development. Importantly, the observed thymus–spleen or thymus–TIL overlaps of TCR clones in Pdcd1 cKO mice significantly exceeded stochastic expectations derived from those from the cross-mouse null model (Fig. 5a, e, h, and Supplementary Fig. 9a–c, j, k). Shared clonotypes differed between B16-OVA and B16-F10 tumors, indicating tumor-specific clonal selection. Since PD-1 deletion occurs after the completion of V(D)J recombination in our model, the overall V(D)J rearrangement landscapes were comparable and remained intact across the compartments (Supplementary Fig. 9d–i, n). Collectively, our results indicate that latent effector differentiation of thymic SP CD8⁺ T cells can directly contribute to the TCR repertoire of TILs, irrespective of tumor immunogenicity, although their magnitude may be regulated by PD-1.

**Fig. 6:**
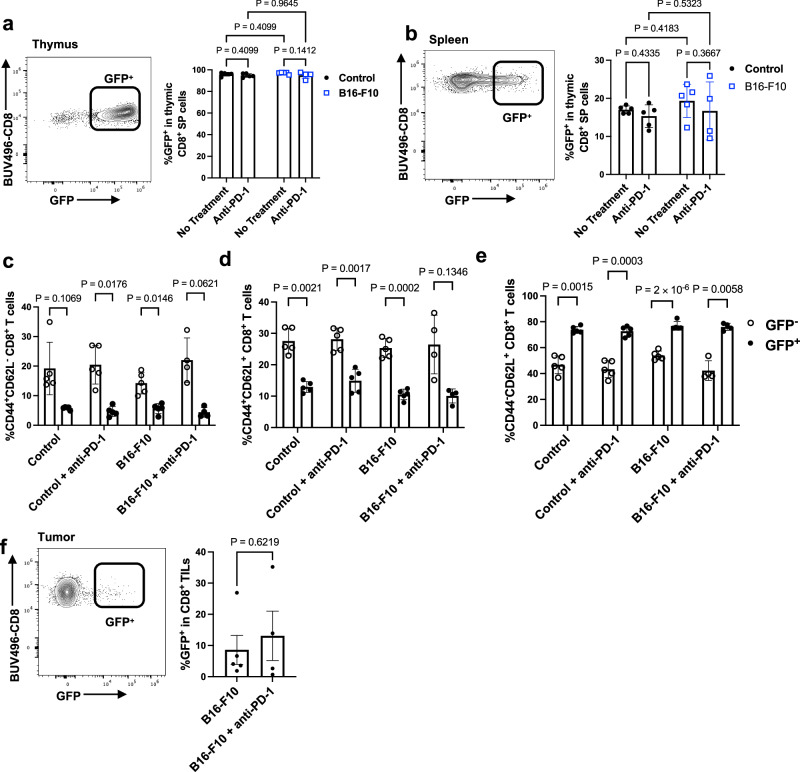
Tracing the recent thymic emigrants in tumor tissues following PD-1 blockade. Gating strategy and percentage of GFP^+^ CD8^+^ T cells in the thymus (**a**), spleen (**b**) or tumor tissues (**f**) after the last treatment of B16-F10 tumors with anti-PD-1 antibody (*n* = 4–5 mice per group) using RAG1-GFP mice. Frequencies of GFP^+^ and GFP^−^ CD8^+^ T cells within splenic effector-like CD44^+^CD62L^−^ (**c**), central memory-like CD44^+^CD62L^+^ (**d**) and CD44^−^CD62L^+^ naïve (**e**) subsets. Each dot indicates one individual mouse, and data are shown from two independent biological experiments. Data are presented as mean ± SEM. For **a**, **b**, **f**, statistical analysis was performed using a mixed-effects model, followed by an uncorrected Fisher’s LSD post hoc test without correction for multiple comparisons (single pooled variance). Statistical analysis of (**c**–**e**) was performed using a mixed-effects model with Geisser-Greenhouse correction, followed by Sidak’s multiple comparisons test. Source data are provided as a Source data file.

To assess whether peripheral PD-1 blockade would affect the thymic output of effector cells in TILs, we evaluated the infiltration of RTEs in tumor tissues. Using Rag1-GFP mice that allow us to track RTEs (GFP^+^ cells) in vivo^31^, we were able to detect GFP^+^ cells in both the thymus and TILs in B16-F10 tumor models in control mice (Fig. 6a–f). Accordingly, GFP^+^CD8^+^ RTEs were enriched with naïve-like (CD44^−^CD62L^+^) cells but not effector/memory-like (CD44^+^CD62L^−^ and CD44^+^CD62L^+^) cells compared to GFP^−^ CD8^+^ non-RTEs (Fig. 6c–e). However, the frequency of GFP^+^CD8^+^ RTEs within tumors was not significantly affected by peripheral anti-PD1 blockade (Fig. 6f). Anti-PD-1 treatment did not affect the frequency of GFP^+^ cells either in the thymus or spleen (Fig. 6a, b). Taken together, our results reveal a direct link between CD8⁺ T cell clones in the thymus and TILs in the absence of PD-1, suggesting a paradigm in which PD-1 regulates immune dynamics from the periphery to the thymus.

### Latent effector differentiation of CD8^+^ T cells can be enhanced by peripheral immunization for tumor immunity

**Fig. 7:**
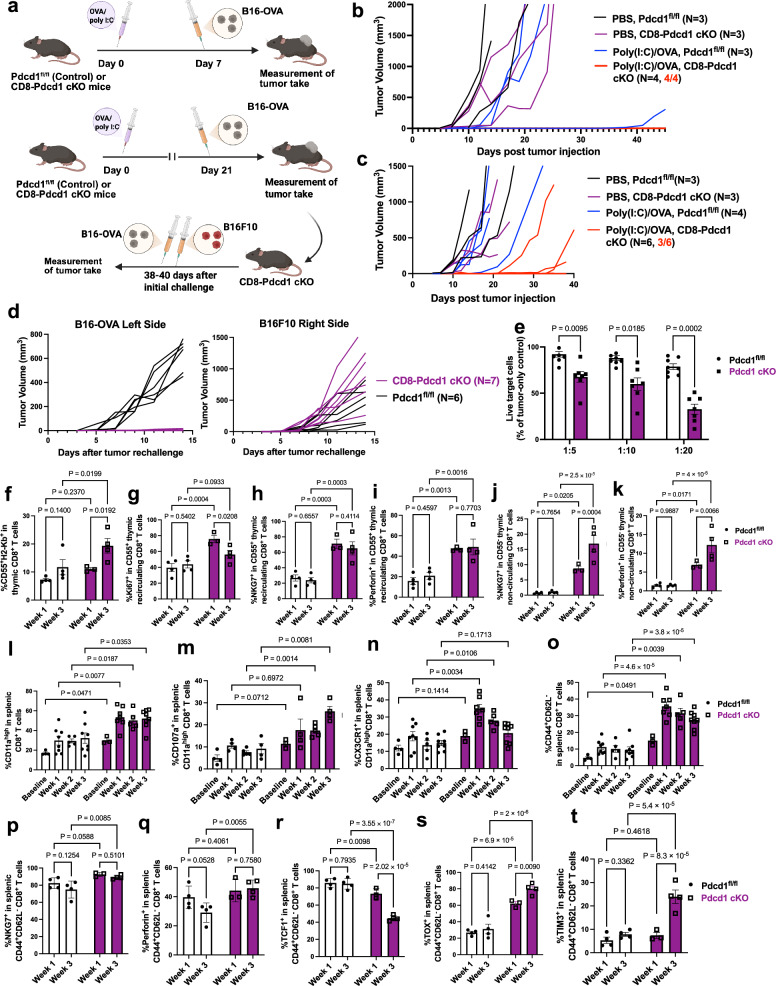
PD-1 deficiency shapes effector- and memory CD8^+^ T cell responses to tumor challenges post-immunization. **a** Schematic of experimental design illustrating poly(I:C)/OVA immunization followed by tumor challenge in Pdcd1^fl/fl^ (control) and CD8-Pdcd1 cKO mice. B16-OVA tumor growth after injection of tumor cells on day 7 (**b**) or day 21 (**c**) after immunization compared with PBS-treated controls (sample sizes are indicated). **d** Tumor rechallenge with B16-OVA (left flank) or B16-F10 (upper right flank) inoculated into CD8-Pdcd1 cKO mice (*n* = 7) that were tumor-free for 38–40 days after initial tumor injection in the lower right flank following immunization. Tumor growth in naïve mice is shown as a control (*n* = 6). **e** Percentages of live P815 target cells after 48-h co-culture with thymic CD8⁺ T cells isolated from Pdcd1^fl/fl^ (*n* = 7) or CD8-Pdcd1 cKO (*n* = 7) mice in the presence of IL-2 (40 IU/mL) and IL-15 (10 ng/mL) at indicated target-to-effector ratios. Frequency of thymic recirculating-like CD55^+^H2-K^b+^ (**f**) and Ki67^+^ (**g**), NKG7^+^ (**h**) or perforin^+^ (**i**) among CD55^+^ thymic recirculating-like or CD55^−^ SP CD8^+^ T cells (**j**, **k**) in Pdcd1^fl/fl^ and Pdcd1 cKO mice after poly(I:C)/OVA immunization for at 1 week (*n* = 4 and *n* = 3) and 3 weeks (*n* = 4 and *n* = 4). Frequency of antigen-primed CD11a^high^ (**l**) and CD107a^+^ degranulation (**m**), CX3CR1^+^ (**n**) effector-like CD44^+^CD62L^−^ among splenic CD8^+^ T cells or in CD11a^high^ subsets (**m**–**o**) at baseline, 1-week, 2-week, and 3-week after immunization (sample sizes are indicated). Frequencies of NKG7^+^ (**p**), perforin^+^ (**q**), TCF1^+^(**r**), TOX^+^(**s**) and TIM3^+^ (**t**) in splenic CD44^+^CD62L^−^ CD8^+^ T cells at 1-week (*n* = 4 and *n* = 3) and 3-week (*n* = 4 and *n* = 4) after immunization in Pdcd1^fl/fl^ and Pdcd1 cKO mice. Schematic **a** created in BioRender. Mao, Z. (2026) https://BioRender.com/pihxqaj. Each line in (**b**–**d**) or each dot in (**e**–**t**) represents one biologically independent mouse, with two to three biological independent experiments. Data are presented as mean ± SEM. Statistical analysis for (**e**,** l**–**o**) was performed using a mixed-effects model with Geisser-Greenhouse correction, followed by Sidak’s multiple comparison test. Statistical analysis for (**h**–**k, p**–**t**) was performed using a mixed-effects model, followed by uncorrected Fisher’s LSD post hoc test without correction for multiple comparisons (single pooled variance).

Although latent effector differentiation of CD8⁺ T cells only partially contributes to tumor immunity against intermediate immunogenic tumors (such as B16-OVA), an important question remains: can this latent effector differentiation in the thymus be enhanced through peripheral immunization? To test that, we immunized both control and CD8-Pdcd1 cKO mice with OVA protein and poly(I:C) followed by a challenge with B16-OVA tumor cells (Fig. 7a). We found that immunized CD8-Pdcd1 cKO mice, but not control mice, had completely no tumor taken on day 7 after immunization (Fig. 7b), a time point corresponding to peak effector cell generation (Supplementary Fig. 10i, j). However, on day 21 after immunization, a time point corresponding to effector T cell contraction (Supplementary Fig. 10i, j), CD8-Pdcd1 cKO mice only partially reject the tumor challenge (Fig. 7c). Among the immunized CD8-Pdcd1 cKO mice that had no tumor taken challenged on day 7, a tumor-specific T cell memory was established, which protected the mice from a second challenge with the same tumor (B16-OVA), but not with B16-F10 (Fig. 7d).

Interestingly, we found immunization in the periphery also enhanced the ex vivo tumor killing ability of thymic CD8^+^ T cells isolated from Pdcd1 cKO mice compared to control mice (Fig. 7e). To that end, we found a trend towards increase in the frequency of recirculating-like thymic CD55⁺H2-K^b^⁺ CD8^+^ T cells in Pdcd1 cKO mice compared with controls after immunization (Fig. 7f), along with an increased proliferative capacity (Ki67^+^) in thymic CD8^+^ T cells (Fig. 7g). In both thymic recirculating-like CD55^+^ and non-recirculating CD55^-^CD8^+^ T cells, we observed increased expression of cytotoxic effector molecules including NKG7 and Perforin in Pdcd1 cKO mice compared with controls after immunization (Fig. 7h–k and Supplementary Fig. 10a–d), which is consistent with their increased tumor killing ability. Notably, we observed an increase of mTOR activity (p-S6^+^) in both CD55^+^ recirculating-like and CD55^−^ non-recirculating thymic CD8^+^ T cells 3 weeks after immunization in the absence of PD-1 (Supplementary Fig. 10e, f), indicating that peripheral immunization may further affect the latent effector differentiation through the mTOR pathway. On the other hand, immunization appeared to trigger a transient overflow of thymic circulating CD8⁺ T cells (CD55⁺CD24⁺) into the spleen in the absence of PD-1 one week after immunization, an effect not attributable to differences in proliferative capacity, as indicated by comparable Ki67 expression (Supplementary Fig. 10g, h).

To explain an enhanced tumor immunity on day 7 after immunization in the absence of PD-1, we found a significant proliferation of CD8^+^ T cells in the spleen after immunization (Supplementary Fig. 10k), particularly a subset of antigen-primed CD11a^high^ CD8^+^ T cells (Fig. 7l). Accordingly, this subset of CD8^+^ T cells was enriched with higher frequencies of CD107a⁺ degranulating cells, CX3CR1⁺ and CD44⁺CD62L⁻ effector memory cells in Pdcd1 cKO mice compared to control mice (Fig. 7m–o), along with proportionally reduction of naïve (CD44^−^CD62L⁺) or central memory (CD44^+^CD62L⁺) cells (Supplementary Fig. 10l, m). While the effector-like CD44^+^CD62L^−^ splenic CD8^+^ T cells had increased expression of cytotoxic effector molecules NKG7 and perforin, exhaustion-associated molecules TOX and TIM-3 were also increased in the absence of PD-1 and peaked at 3 weeks after immunization along with a decrease of stem-like marker TCF1 (Fig. 7p–t). In contrast to an increased mTOR activity in the thymic CD8^+^ T cells (Supplementary Fig. 10e), immunization caused a decrease of mTOR activity in splenic CD8⁺ T cells (Supplementary Fig. 10n), suggesting the regulation of PD-1 in mTOR activity could be context dependent. Collectively, our findings suggest that peripheral immunization further enhances latent effector differentiation of CD8⁺ T cells in the absence of PD-1, thereby promoting antitumor immunity; however, the progression to an advanced exhaustion program may compromise the durability of T cell memory.

## Discussion

The prevailing view in T cell immunology is that naïve CD8⁺ T cells exit the thymus in a functionally quiescent state, and that cytotoxic effector differentiation occurs exclusively in the periphery following antigen encounter^32^. It has been proposed that effector CD8⁺ T cell fate is at least partly established as their precursor cells undergo maturation during thymic development^33^. However, the molecular mechanisms underlying this pre-programming in the thymus remain poorly defined. In this context, our study provides direct evidence showing that thymic SP CD8^+^ T cells are poised to a latent effector differentiation program to acquire cytotoxic capacity after thymic positive selection, but this process is tightly regulated by PD-1. Since peripheral immunization, but not PD-1 blockade, can enhance latent effector differentiation, this unique T cell differentiation program may involve a putative cell-intrinsic PD-1 signaling mechanism, as recently reported^23^.

Our findings help resolve a long-standing debate regarding whether the thymus supports effector-like CD8⁺ T cell differentiation: this differentiation occurs post-thymic selection but is masked by PD-1 signals. In the absence of PD-1, we uncover an underappreciated dimension of thymic T cell development—specifically, the emergence of cytotoxic-like CD8⁺ thymocytes. This latent effector differentiation would allow CD8^+^ T cells to rapidly acquire effector function in the periphery when they encounter pathogens or malignant antigens, thereby protecting the host from infections and tumors. However, this also raises the risk of normal tissue damage if their effector function is exaggerated. To this end, PD-1, as a checkpoint, is embedded in the thymus during the selection of SP CD8^+^ T cells to prevent exaggerating their effector functions.

Contrary to a recent study showing PD-1 is required to preserve and expand high-affinity, stem-like CD8^+^ TCR clones in tumor-draining lymph nodes^34^, we believe high-avidity TCRs described in the study may not accurately represent the spectrum of TCR affinities present in the thymus, as such TCRs with excessively high affinity for self-antigen are eliminated during negative selection. Consequently, TCRs that successfully pass thymic selection are expected to fall within an intermediate range of affinity or avidity. Our findings demonstrate that PD-1 signaling restricts these intermediate-affinity TCRs from initiating latent effector differentiation immediately after thymic selection, a critical checkpoint that occurs much earlier than the later PD-1–mediated limitation of stem-like high-affinity TCRs differentiating into effector cells in lymph nodes, as reported^34^.

The presence of cytotoxic-like thymic CD8^+^ T cells prompted us to consider the mechanisms underlying their differentiation and acquisition of cytotoxic potential, particularly after the thymic positively selecting TCR signal. Emerging evidence has shown NKG7 transcription in thymic mature CD8^+^ T cells^4,35^. Although these studies did not further characterize the cytotoxic function of these cells, a second wave of TCR signaling was identified that occurs specifically in CD8-fated thymocytes during lineage commitment^4^. Consistence with this, our findings that thymic SP CD8^+^ T cells exhibit a trend towards increased Nur77 expression, supporting the notion that these cells recently experienced an additional round TCR stimulation for acquiring the cytotoxic effector function after the positive selecting TCR signal for CD8^+^ T cell lineage commitment. However, our study only supports PD-1 to act as a regulator of latent effector differentiation in thymic SP CD8^+^ T cells. Further studies should determine other regulators in this unique T cell differentiation program in positively selected thymic CD8⁺ T cells in the context of TCR signaling, immune checkpoint signaling and cytokine cues or a combination of these factors.

One surprising finding from our study is the presence of shared TCR clonotypes between the thymus and tumor tissues in PD-1-deficient CD8⁺ T cells, regardless of tumor immunogenicity. This suggests that direct thymic output can shape the TCR repertoire of TILs, a process primarily regulated by PD-1 signaling rather than driven by tumor antigens. Our results provide additional evidence supporting the tumor immunosurveillance theory, which posits that continuous thymic output supplies a diverse TCR repertoire to screen tumor antigens in the periphery. In other words, comprehensive tumor immunosurveillance appears to rely on a stochastic contribution of thymic-derived CD8⁺ T cells rather than the selection of a fixed set of tumor-specific clones. However, this stochastic contribution carries the risk of generating self-reactive T cell clones. PD-1 signaling functions as a critical checkpoint to prevent both overt effector differentiation, which could induce autoimmunity, and terminal effector differentiation, leading to T cell exhaustion and compromised durable immunity^36^. Another interesting observation is that OVA-specific TCR clones were detected only in tumor tissues, not in the thymus. OVA protein, widely used as a surrogate tumor antigen, is not a natural antigen involved in thymic selection, which may explain its absence in the thymus. How OVA-specific TCRs arise in the periphery remains an open question. Addressing this question could help identify neoantigen-specific TCRs and guide the design of tumor vaccines to selectively expand these clones. Our model system provides a unique resource to explore this possibility.

Our single-cell RNA sequencing and flow cytometry analyses across the thymus, peripheral tissues, and tumor microenvironment identified NKG7 as an emerging functional marker of latent effector differentiation of CD8⁺ T cells from the thymus to the periphery, particularly in establishing robust tumor immunity. Consistent with this observation, we revealed that the presence of NKG7⁺ versus NKG7⁻ CD8⁺ T cells within tumor tissues correlates with signatures of reduced PD-1 signaling. Functionally, dual deletion of PD-1 and NKG7 in CD8⁺ T cells resulted in compromised, though not completely abolished, antitumor immunity, indicating that NKG7 is likely a key target molecule of PD-1 regulation. Thus, our findings suggest that NKG7 may serve not only as a useful biomarker for stratifying responses to PD-1 blockade but also as a gateway for dissecting how latent effector differentiation is programmed in the thymus.

In summary, our findings reveal a latent effector differentiation program of CD8⁺ T cells in the thymus, regulated by PD-1 signaling in the context of effector gene expression. We demonstrate that PD-1 limits the contribution of thymic effector-like CD8⁺ T cells to tumor immunosurveillance by shaping the TCR repertoire within tumor tissues, regardless of tumor immunogenicity. While latent effector differentiation of thymic CD8⁺ T cells enables a rapid peripheral response to malignant cells, PD-1 restrains this process to prevent overt or terminal effector differentiation, which could compromise balanced and durable immunity. Clinically, these insights suggest that PD-1 blockade alone may not optimize long-term antitumor immunity, as accelerated exhaustion can limit sustained responses. Future therapeutic strategies should consider combining PD-1 inhibition with approaches that promote early effector expansion while preserving memory potential, such as transient immunomodulation or metabolic support, to balance immediate tumor control with durable immunity. Additionally, biomarkers like NKG7 may help stratify patients likely to benefit from such combination strategies.

## Methods

### Mouse

All experimental procedures were approved by the Institutional Animal Care and Use Committee (IACUC) at Mayo Clinic Rochester. Animals were maintained under specific pathogen-free conditions and housed in filter-top cages with access to food pellets and water under controlled environmental conditions (20–22 °C, 30–70% relative humidity) and a 12 h light/12 h dark cycle. Mice were euthanized by carbon dioxide inhalation in accordance with approved institutional protocols.

*Pdcd1*^flox/flox (fl/fl)^ mice on a C57BL/6 background were a kind gift from Dr. Vassiliki A. Boussiotis from Harvard Medical School, and the protocol for generating these mice has been previously described^37^. *Nkg7*^fl/fl^ mice on a C57BL/6 background was generated by inGenious Targeting Laboratory (Ronkonkoma, NY) and details have been described^38^. E8I CD8-Cre mice were purchased from the Jackson Laboratory (C57BL/6-Tg (Cd8a-cre)1Itan/J, Strain stock #: 008766). The *Pdcd1*^fl/fl^ E8I CD8Cre (CD8-Pdcd1 cKO) mice were generated by crossing *Pdcd1*^fl/fl^ mice with E8I CD8-Cre mice. The *Pdcd1*^fl/fl^ *Nkg7*^fl/fl^ E8I CD8Cre^+^ (CD8-Pdcd1-Nkg7 dcKO) mice were generated by crossing *Pdcd1*^fl/fl^ mice and *Nkg7*^fl/fl^ mice to generate *Pdcd1*^fl/fl^ *Nkg7*^fl/fl^ and cross with E8I CD8-Cre mice. *Pdcd1*^fl/fl^ mice were genotyped using the primer pair: 1: 5′ TAT CCC TGT ATT GCT GCT GCT G 3′ and 2: 5′ AAT GAA TTG AGG AGT AGG GCC TG3′. E8I CD8Cre were genotyped using the following primer 1: 5′ CAA TGG AAG GAA GTC GTG GT 3′. Primer 2: 5′ TGG GAT TTA CAG GGC ATA CTG 3′ and primer 3: 5′ CAC ACA TGC AAG TCT AAA TCA GG 3′. RAG1-GFP mice on a C57BL/6 background was generated as previously described^31^ and obtained from Dr. Virginia Shapiro (Mayo Clinic). In all experiments, male and female mice used were after 8–12-week-old and were randomly assigned for experiments. The number of animals used in each experiment is indicated in the corresponding figure legends. Mice were randomly assigned to experimental groups regardless of sex. All experimental procedures were approved by the institutional animal care and use committee at Mayo Clinic Rochester (IACUC: A00006353-21-R24, A00002759-17-R23).

### Tumor cells, media, and cell culture

Dulbecco’s modified Eagle medium (DMEM, Gibco, Cat#11885-084), DMEM with 4.5 g/L glucose, L-glutamine and sodium pyruvate (DMEM^high^, Corning, Cat #10-013-CV) and RPMI 1640 (Corning, Cat#10-040-CV) were supplemented with 10% heat-activated fetal bovine serum (FBS, Gibco, Cat # A52567-01) and 10 mM HEPES buffer (Corning, Cat # 25-060-CI) and 1× Penicillin/Streptomycin (Cellgro, Cat#30-002-CI) for a complete medium. MC38 colon adenocarcinoma cells were purchased from MilliporeSigma (SCC172) and has been maintained using DMEM^high^ complete medium. B16-F10 mouse melanoma cell line was purchased from ATCC (CRL-6475) and cultured using DMEM complete medium. B16-OVA mouse melanoma cell line was a gift from Dr. Richard Vile at Mayo Clinic, Rochester and cultured using RPMI 1640 complete medium with 0.8 mg/mL Geneticin Selective Antibiotic (G418 Sulfate, Gibco, Cat#11811031). Cell lines were screened for *Mycoplasma* contamination and authenticated by short tandem repeat profiling (B16 at American Type Culture Collection and MC38 at IDEXX). P815 mastocytoma cells were obtained from the American Type Culture Collection (ATCC TIB-64) and cultured using DMEM complete medium.

### Tumor models and in vivo treatment

*Pdcd1*^fl/fl^ E8I CD8Cre^−^ (control), *Pdcd1*^fl/fl^ E8I CD8Cre^+^ (CD8-*Pdcd1* cKO) and *Pdcd1*^fl/fl^ *Nkg7*^fl/fl^ E8I CD8Cre^+^ (CD8-Pdcd1 Nkg7 dcKO) mice were subcutaneously injected with 0.5 million tumor cells resuspended in 100 μL PBS. For the immune checkpoint blockade study, naïve mice were intraperitoneally injected with 300 μg anti-PD-1 antibody (clone G4, Sigma-Aldrich, Cat# MABC1132-100UG) every 2 days (day 1, 3, 5, 7, and 9) for a total of five doses. For GFP^+^ tracking RTEs tracking experiment, RAG1-GFP mice was subcutaneously injected with 0.5 million tumor cells resuspended in 100 μL PBS. Mice were intraperitoneally injected with 200 μg anti-PD-1 antibody (Clone G4, Sigma-Aldrich, Cat# MABC1132-100UG) starting from day 7 every other 2 days (day 7, 10, 13, 16, and 19) for a total of five doses. For ovalbumin (OVA) immunization studies, 50 μg polyinosinic-polycytidylic acid (Poly(I:C), Novus, Cat#NBP2-25288) and 500 μg OVA peptide (MilliporeSigma, Cat# A5503) were mixed in 200 μL PBS and intraperitoneally injected into the mice. All animal experiments were conducted in accordance with protocols approved by the Institutional Animal Care and Use Committee (IACUC) at Mayo Clinic. Tumor growth was measured every 2–3 days starting from day 7 post-tumor injection until the euthanasia endpoint in compliance with animal care guidelines. Perpendicular tumor diameters were measured using a digital caliper (Carbon Fiber Composite, Fisher Scientific). Tumor size was calculated using the formula (length × width²)/2. Humane endpoints were predefined based on tumor burden and animal welfare criteria. The maximal permitted tumor volume was 2000 mm³, or earlier if animals exhibited signs of distress, including ulceration and impaired mobility. At no point were the approved tumor size limits exceeded in this study.

### Isolation of mouse CD8^+^ T cells

Mouse splenic and thymic CD8^+^ T cells were isolated using the CD8a^+^ T Cell Isolation Kit (Miltenyi Biotec, Cat#130-104-075). Mouse splenic naïve CD8^+^ T cells were isolated using Naive CD8a^+^ T Cell Isolation Kit, mouse (Miltenyi Biotec, Cat#130-096-543). For isolating CD8^+^ TILs, tumors were first cut into small pieces of 2–4 mm and digested using a tumor dissociation kit at 37 °C for 45 min with gentle agitation (Miltenyi Biotec, Cat#130-096-730). Dead cells were further removed using the Dead Cell Removal kit (Miltenyi Biotec, Cat# 130-090-101). CD8^+^ TILs were then isolated using CD8 (TIL) microbeads, mouse (Miltenyi Biotec, Cat#130-116-478).

### Flow cytometry and antibodies

A complete list of antibodies is provided in Supplementary Table 1. Flow cytometry was performed using a Cytek Aurora spectral flow cytometer (Cytek Biosciences). Single-cell suspensions were prepared from thymus, spleen, and TILs as described above. For viability staining, cells were incubated with Ghost Dye Violet 510 (Cytek Biosciences) or Zombie UV (BioLegend) diluted in PBS for 20 min at room temperature, protected from light. After washing, cells were stained with fluorophore-conjugated antibodies against surface markers in FACS buffer (PBS supplemented with 2% FBS and 2 mM EDTA) for 20–30 min at 4 °C. For intracellular staining, cells were fixed and permeabilized using Fixation/Permeabilization Concentrate (Cat# 00-5123-43, eBioscience) and Fixation/Permeabilization Diluent (Cat# 00-5223-56, eBioscience) mixed at a 1:3 ratio, for 20 min at room temperature or overnight at 4 °C. Cells were then washed with 1× permeabilization buffer (Permeabilization Buffer 10×, Cat# 00-8333-56, Invitrogen) and stained with intracellular antibodies diluted in permeabilization buffer.

A rabbit polyclonal antibody to mouse NKG7 (UniProtKB:Q99PA5) was obtained by immunizing a rabbit with keyhole limpet hemocyanin-conjugated NKG7 peptide DFWIVATGPHFSAHSGLWPTSQET (Cocalico Biologicals, Reamstown, PA). The anti-NKG7 polyclonal rabbit serum was affinity-purified using Sulfolink (Cat#20401, Thermo Fisher Scientific, Waltham, MA) as per the manufacturer’s instructions. NKG7 antibody was then labeled with Alexa Fluor 594 antibody labeling (Cat# A20185, Invitrogen) based on the manufacturer's instructions. For the PD-1 antibody binding assay, splenocytes were analyzed at baseline and after Dynabeads Mouse T-Activator CD3/CD28 (Thermofisher, Cat# 11456D) stimulation for 24 h at 37 °C. Cells were first incubated with 20 μg/mL Hamster anti-PD-1 antibody (Clone G4) for 20 min at room temperature, followed by staining with goat anti-Armenian Hamster IgG (H + L) Alexa Fluor 555 secondary antibody (Thermofisher, Cat# A78964) for 15 min. Cells were subsequently stained for surface markers prior to flow cytometric analysis.

Mitochondrial reactive oxygen species (ROS) were measured using MitoSOX Red (Thermo Fisher Scientific) according to the manufacturer’s instructions. Cells were resuspended in PBS and incubated with MitoSOX at a final concentration of 5 μM for 15–30 min at 37 °C, protected from light. Control samples received an equivalent volume of DMSO. Following incubation, cells were washed with PBS and subsequently stained for viability and surface markers prior to flow cytometric analysis. For CD107a degranulation and intracellular cytokine assays, freshly isolated thymocytes or splenocytes were stimulated with PMA (50 ng/mL, Sigma, Cat# P1585) and ionomycin (500 ng/mL, Sigma, Cat# I0634) or anti-CD3/CD28 (Thermofisher, Cat# 11456D) for 4–5 h at 37 °C in the presence of brefeldin A (1×; BioLegend, Cat# 420601), monensin (1×; BioLegend, Cat# 420701), and APC-conjugated anti-mouse CD107a antibody (BioLegend, Cat# 505809, clone 1D4B). Following stimulation, cells were stained for viability and surface markers prior to fixation and intracellular staining. Data acquisition was conducted using the Aurora’s full-spectrum detection system. Compensation and spectral unmixing were performed using SpectroFlo software (Cytek Biosciences). Gating strategies were applied using FlowJo software (v10.0.0). Fluorescence-minus-one and unstained controls were included for accurate gating and to validate antibody specificity.

### Acute PD-1 blockade on isolated naïve T cells in vitro

Naïve CD8^+^ T cells were isolated from age-matched, tumor-free young mice (6-week-old) using Naive CD8a^+^ T Cell Isolation Kit, mouse (Miltenyi Biotec, Cat#130-096-543). Pdcd1^fl/fl^ and Pdcd1 cKO naïve CD8⁺ T cells were stimulated with anti-CD3/CD28 (Thermofisher, Cat# 11456D) for 24 h. To model acute PD-1 blockade, these Pdcd1^fl/fl^ naive CD8⁺ T cells were additionally cultured in the presence of 20 µg/mL anti-PD1 antibody. Pharmacodynamic PD-1 target engagement assay was performed by incubating splenic CD8^+^ T cells from Pdcd1^fl/fl^ and Pdcd1 cKO with Hamster anti-PD-1 antibody (Clone: G4) at baseline or following 24 h anti-CD3/CD28 stimulation. These cells were further stained with Alexa Fluor 555-conjugated anti-Hamster secondary antibody (Invitrogen, Cat#A78964) for flow cytometric analysis.

### Single-cell RNA sequencing and analysis

Mouse thymic CD8^+^ T cells, splenic CD8^+^ T cells and CD8^+^ TILs were isolated from adult mice (>8 weeks old) for single-cell RNA sequencing. Single-cell libraries were prepared using Fluent Biosciences Pre-Templated Instant Partitions Sequencing (PIPseq) v3 Kit according to the manufacturer’s protocol. Briefly, cells were first filtered through a 40-μm cell strainer to remove debris and aggregates. The viability was assessed through Trypan Blue cell counting, and samples with more than 80% viability were processed for sample preparation. Each sample was loaded to the PIPseq microfluidic system, which partitioned individual cells into droplets containing molecular barcodes for unique transcript identification. During reverse transcription, unique molecular identifiers (UMIs) and well-specific barcodes were incorporated to enable precise quantification and tracking of individual transcripts. Subsequently, cDNA synthesis and amplification were performed to maximize transcript coverage and minimize bias. Amplified cDNA was purified and quality-checked using an Agilent 2100 Bioanalyzer. Following quality assessment, sequencing libraries were constructed by fragmenting cDNA, performing end-pair and A-tailing and ligating adapters compatible with the sequencing platform.

The library was sequenced on an Illumina NovaSeq platform using the S4 flow cell platform in a paired-end format. Read 1 (28 bp) was used to sequence the transcript barcode, while read 2 (91 bp) captured the transcript sequence. Unique Index read pairs (i7: 8 bp, i5: 8 bp) were used for demultiplexing. The sequencing was covered for an average depth of 40,000 reads per cell. Raw sequencing data were processed using Fluent Biosciences’ PIPseq analysis pipeline (v3.2). Reads were aligned to the GRCm39 reference genome, and UMIs were used to deduplicate transcripts. Cells with fewer than 500 detected genes, greater than 20% mitochondrial transcript counts, or fewer than 1000 UMI counts were excluded from further analysis.

Quality control, normalization, and downstream analysis were performed in R using Seurat (v4.3.0)^39^. Data were normalized using SCTransformed, and highly variable genes were identified for dimensionality reduction^40^. Principal component analysis was conducted, and the top 20 principal components were used for UMAP visualization. Clustering was performed using the Louvain algorithm implemented based on shared nearest neighbor graph construction in principal component space. Resolution parameters were iteratively optimized to identify biologically meaningful clusters. Subclustering was performed where necessary to further resolve heterogeneous populations. For integrated analyses, datasets were merged in Seurat based on shared gene features. Batch effects were corrected using Harmony (v1.0), which aligned datasets while preserving biological variation. The integrated dataset was subsequently scaled and used for downstream visualization and clustering. Differential expression analysis was performed using Seurat to identify genes differentially expressed between clusters or predefined groups. Representative differentially expressed genes (DGEs) in thymus, spleen, and tumor samples, as well as the complete DGE lists, are provided in Supplementary Data. Gene sets identified from differential expression analyses were uploaded to Ingenuity Pathway Analysis (IPA; Qiagen) for pathway enrichment analysis. Detailed pathway results are provided in the Source data file.

### TCR sequencing analysis

TCR repertoire profiling was performed using the Parse Evercode TCR platform according to the manufacturer’s instructions (Parse Biosciences). Single cell suspensions of thymic CD8^+^ T cells, splenic CD8^+^ T cells and tumor-infiltrating CD8^+^ T cells were prepared as described above and processed using the Evercode TCR kit. cDNA libraries were generated from whole-transcriptome amplification products containing V(D)J segments spanning the CDR3 regions of TCRα (TRA) and TCRβ (TRB) chains, followed by ligation of Illumina-compatible adapters. Libraries were sequenced on an Illumina NovaSeq platform (2 × 250 bp, SP flow cell) to achieve a minimum depth of 20,000 reads per cell. TCR clonotype assembly, sequence annotation, and clonotype calling were performed using the Parse Biosciences TCR analysis pipeline. Productive clonotypes were defined as unique paired TRA–TRB CDR3 nucleotide sequences with detectable template counts.

To control for differences in sequencing depth and clonal distribution, thymus and matched spleen (baseline) or TIL repertoires from each mouse were normalized by rarefaction. Within each mouse pair, repertoires were downsampled without replacement to the productive template count of the lower-depth sample. The productive template counts used for rarefication are provided in Supplementary Table 2. Rarefaction was repeated for 1000 independent iterations, and mean similarity metrics were used for downstream analyses. Repertoire sharing between tissues was quantified using two complementary similarity metrics: 1. Jaccard index, defined as the number of shared unique clonotypes divided by the total number of unique clonotypes across both samples (presence/absence-based similarity). 2. Morisita–Horn index, a frequency-weighted similarity measure accounting for clonotype abundance and clonal expansion.

To determine whether observed repertoire overlap exceeded stochastic expectations, a within-mouse permutation test was performed. For each mouse, thymic and spleen/TIL clonotype identities were pooled while preserving marginal frequency distributions and repertoire sizes. Clonotype labels were randomly reassigned between tissues 1000 times to generate empirical null distributions for both similarity metrics. Observed values were compared against these null distributions to compute empirical P values. To estimate background public TCR sharing, thymic repertoires were cross-compared with spleen or TIL repertoires from non-matched mice processed in parallel. These cross-mouse comparisons served as an external null benchmark. For visualization of clonal relationships (Fig. 5d, k, l), the original, unrarefied top 100 clonotypes per sample were ranked by template count and frequency. Corresponding TRA and TRB CDR3 sequences and clone counts are provided in the Source data file.

### In vivo CD45 intravenous labeling

Pdcd1^fl/fl^ and CD8-Pdcd1 cKO mice were injected intravenously with 2 μg of FITC anti-mouse CD45 antibody (Biolegend, Cat#103118, clone 30-F11) in 200 μL of PBS via the retro-orbital sinus 3 min before euthanasia. Thymus, spleen and peripheral blood were immediately harvested, and single-cell suspensions were prepared for flow cytometry. CD45^+^ cells were considered as circulating, while CD45^−^ cells were considered as non-circulating.

### Immunofluorescent staining of thymic CD8^+^ T cells

The thymus was quickly removed and fixed overnight in 4% PFA with gentle shaking, embedded in cryoprotective medium (OCT compound), and cut into 10 μm-thick sections. Sections were subjected to immunofluorescence staining as described^41^. Briefly, slides were first washed in PBS buffer and permeabilized in 0.15% Triton X-100 solution, followed by blocking in 5% normal goat serum in PBS. Rabbit anti-mouse NKG7 (1:500 dilution) and rat anti-mouse CD8a (1:500 dilution, Biolegend, clone 53-6.7, Cat#100702) antibodies were then applied to slides for overnight incubation. To detect and amplify the stained signal of CD8a, first, goat anti-rat IgG mouse absorbed biotinylated antibody (1:100 dilution, Vector Laboratories, Cat#BA-9401.5) was added to the slide for 1 h incubation at room temperature, and then followed with staining of Streptavidin, DyLight 488 (Vector Laboratories, Cat# SA-5488-1). For detection of NKG7, a goat anti-Rabbit IgG (H + L) Cross-Adsorbed Secondary Antibody, Alexa Fluor™ 568 (1:500 dilution, Invitrogen, Cat# A-11011) were incubated for 1 h at room temperature. Slides were washed and stained with Hoechst (1:500 dilution) in PBS buffer and covered with anti-fade mounting medium. Confocal images were collected with an LSM-800 laser scanning confocal microscope with a 40×-water Plan-Apochromat objective lens.

### Spatial transcriptomics of human bladder cancer tissue

Human muscle-invasive bladder cancer tissue slides were selected with the approval of Mayo Clinic IRB# 21-000078. These patients underwent radical cystectomy but did not receive neoadjuvant therapies. Tissue sectioning and immunohistochemical (IHC) staining were conducted at the Pathology Research Core (Mayo Clinic, Rochester, MN) using the Leica Bond RX automated stainer (Leica). Formalin-fixed, paraffin-embedded (FFPE) tissue sections (5 μm thick) were mounted on glass slides and processed for GeoMx DSP. Tissue sections were deparaffinized, rehydrated, and subjected to antigen retrieval before incubation with fluorescently labeled morphology markers to visualize regions of interest (ROIs). Slides were incubated with rabbit anti-human NKG7 monoclonal antibody (1:1000, Dong Lab, described previously^38^) and mouse anti-human CD8 antibody (1:500, RIV11 clone, Thermo Scientific, MA5-48276), followed by Goat anti-Rabbit IgG (H + L) Cross-Adsorbed Secondary Antibody, Alexa Fluor 532 (Thermofisher Scientific, Cat#A-11009) and Goat anti-Mouse IgG (H + L) Cross-Adsorbed Secondary Antibody, Alexa Fluor 647 (Thermofisher Scientific, Cat#A-21235). ROIs were selected based on fluorescence imaging using the GeoMx instrument. The selected ROIs were then hybridized with barcoded RNA detection probes (GeoMx DSP RNA assays). Following hybridization, ultraviolet (UV) light was used to release ROI-specific oligonucleotide barcodes. Released barcodes were collected and amplified by PCR. Library quality and quantity were assessed using an Agilent 2100 Bioanalyzer and qPCR. Sequencing was performed on an Illumina NovaSeq S4 platform, generating paired-end reads. Data analysis was conducted using the GeoMx NGS Pipeline software for spatial alignment, normalization, and quantification of transcript counts. Further analyses, including ROI-specific differential gene expression and pathway enrichment, were performed using the GeoMx DSP Analysis Suite.

### Thymocyte-mediated tumor killing assay

P815 target tumor cells were plated at 10,000 cells per well in RPMI complete medium in a round-bottom 96-well plate. Hamster anti-CD3 monoclonal antibody (clone 145-2C11, eBioscience, Cat #16-0031-82) was added at the final concentration of 5 µg/mL and incubated at room temperature for 30 min for coating on P815 cells, and then the antibody was washed away before the addition of thymocytes. Freshly isolated thymic Pdcd1^fl/fl^ and Pdcd1 cKO SP CD8^+^ T cells at baseline or after immunization were added to each well following a ratio of target to effector 1:20, 1:10, 1:5, and 1:0. Co-culture of the thymic CD8^+^ T cells and P815 target cells were performed for 48 h at 37 °C in a humidified incubator with 5% CO₂. Total number of remaining P815 target cells were counted using flow cytometry after gating out the dead cells and T cells in comparison with control wells with target cells only.

### Statistical analysis

All statistical analyses were performed using GraphPad Prism (v10) and R (4.4.1). Data are presented as mean ± SEM unless indicated otherwise. Statistical significance was defined as two-sided *P* < 0.05 after appropriate multiple-testing correction. For all in vivo mouse experiments, the biological unit of replication was an individual mouse. Two to three technical replicates were averaged per mouse prior to statistical testing. For spatial transcriptomic analyses, the biological unit of replication was the individual patient. For TCR repertoire analyses, the biological unit was the mouse (thymus–spleen or thymus–TIL sample pair). Rarefaction iterations were used to estimate sampling variance, but were not considered independent replicates.

Comparisons between two independent groups were performed using unpaired, two-tailed t-tests. For experiments involving more than two groups, one-way ANOVA followed by Tukey’s multiple-comparisons test was used. For experiments involving repeated measures, mixed-effects models were applied. Where appropriate, the Geisser-Greenhouse correction was used. Post hoc comparisons were performed using Sidak’s or Fisher’s LSD tests as indicated in the corresponding figure legends. Survival curves were generated using the Kaplan–Meier method and compared using the log-rank (Mantel–Cox) test. Hazard ratios (HR) and 95% confidence intervals (CI) were calculated using Cox proportional hazards regression.

Single-cell RNA-seq data were processed using Seurat. Differential expression analyses at the single-cell level were performed using the Wilcoxon rank-sum test with Benjamini–Hochberg false discovery rate (FDR) correction. These results are descriptive and hypothesis-generating rather than inferential at the population level. For spatial transcriptomic comparisons between NKG7⁺CD8⁺ and NKG7^−^CD8⁺ T cells, raw counts were aggregated at the patient level (pseudobulk) prior to differential expression analysis, with the patient defined as the biological unit of inference. Differential expression was performed using DESeq2 with the patient included as a blocking factor. Multiple testing correction was performed using the Benjamini–Hochberg FDR method, and adjusted *P* values (*p*adj) are reported.

### Reporting summary

Further information on research design is available in the Nature Portfolio Reporting Summary linked to this article.

## Supplementary information

Supplementary Information

Description of Additional Supplementary Files

Supplementary Data 1

Reporting Summary

Transparent Peer Review File

## Source data

Source Data

## Data Availability

All sequencing data generated in this study have been deposited in the Gene Expression Omnibus (GEO) database. Single-cell RNA sequencing (scRNA-seq) data are available under accession number GSE328625. TCR sequencing data are available under accession number GSE328442. Spatial transcriptomics data are available under accession number GSE328664. All data supporting the findings of this study are available within the paper and its Supplementary Information files or from the corresponding author on request. Source data are provided with this paper.
