# Supplementary material for: PD-1 regulates latent effector differentiation of thymic cytotoxic CD8+ T cells
Source: Nat Commun. 2026 May 23;17:6769. doi: 10.1038/s41467-026-73392-7 (PMC13385908; doi:10.1038/s41467-026-73392-7)
Supplement: Supplementary file 1 — Supplementary Information [file 41467_2026_73392_MOESM1_ESM.pdf]

Supplementary Information

PD-1 regulates latent effector differentiation of thymic cytotoxic CD8$^+$ T cells

Zhiming Mao, Jacob B. Hirdler, Joanina K. Gicobi, Li Ding, Mark A. Maynes, Michelle A. Hsu, Emilia R. Dellacecca, Wenjing Zhang, Jacob J. Teske, Ying Li, Aubrey Y. Liew, Geoffrey Zhao, Adrian T. Ting, Virginia M. Shapiro, Fabrice Lucien-Matteoni, Henrique Borges da Silva, Daniel D. Billadeau, Haidong Dong*

*Corresponding Author:

Haidong Dong (Dong.Haidong@mayo.edu)

This file includes:

Supplementary Figures 1-10

Supplementary Table 1-2

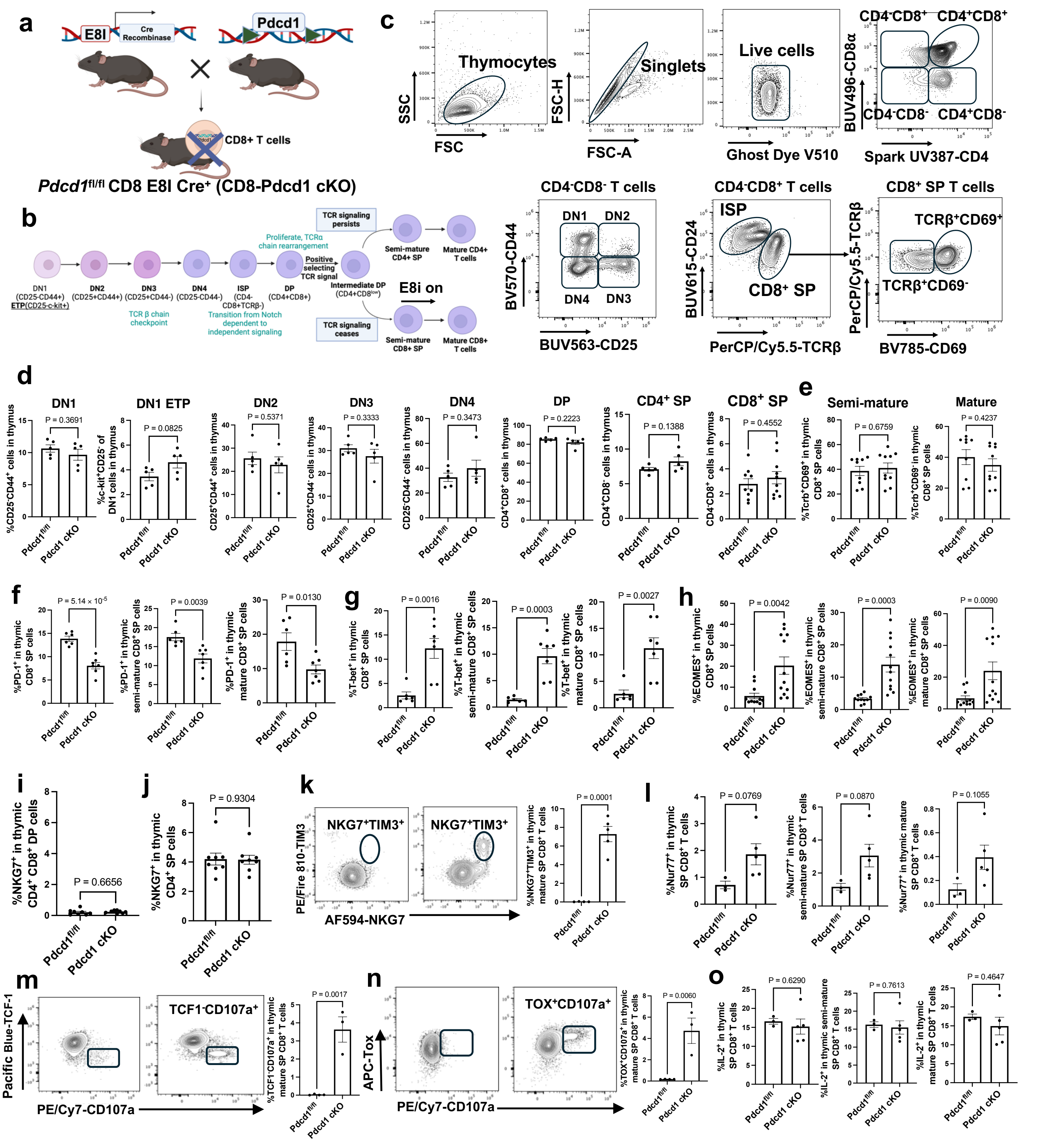

Supplementary Figure 1. Flow cytometry analysis of the thymic profile of single positive CD8⁺ T cells. (**a**) Schematic presentation of the production of *Pdcd1*$^{fl/fl}$ CD8 E8I Cre⁺ (CD8-Pdcd1 cKO) mice. **(b)** Schematic presentation of the stage in which E8i enhancer works during thymic T cell development. (**c**) Flow cytometry analysis of thymic post-positively selected single positive (SP), semi-mature (TCRβ⁺CD69⁺) and mature (TCRβ⁺CD69⁻) SP CD8⁺ T cells. ISP: immature single positive. (**d**) Frequency of double negative (DN)1, DN1 ETP (early T lineage precursor), DN2, DN3, DN4, double positive (DP), CD4⁺ SP (n=5 and n=5) and CD8⁺ SP (n=9 and n=10) thymocytes in Pdcd1$^{fl/fl}$ and Pdcd1 cKO mice. (**e**) Frequency of thymic semi-mature and mature SP CD8⁺ T cells in all CD8⁺ SP thymocytes⁺ in Pdcd1$^{fl/fl}$ (n=9) and Pdcd1 cKO (n=10) mice. (**f**-**h**) Frequency of

PD-1$^+$ (**f,** n=6 and n=6), T-bet$^+$ (**g,** n=6 and n=7) and EOMES$^+$ (**h,** n=11 and n=12) in thymic SP CD8$^+$ cells, semi-mature and mature CD8$^+$ SP cells. (**i, j**) Frequency of NKG7$^+$ in thymic CD4$^+$CD8$^+$ double positive (DP) cells (**i**) and CD4$^+$ SP cells (**j**) in Pdcd1$^{fl/fl}$ (n=11) and Pdcd1 cKO (n=12) mice. (**k**) Contour plot (left) and frequency (right) of NKG7$^+$TIM3$^+$ in mature CD8$^+$ SP thymocytes in Pdcd1$^{fl/fl}$ (n=4) and Pdcd1 cKO (n=5) mice. (**l**) Frequencies of Nur77$^+$ in thymic total, semi-mature, and mature SP CD8$^+$ T cells in Pdcd1$^{fl/fl}$ (n=3) and Pdcd1 cKO (n=5) mice. (**m, n**) Contour plot of TCF1$^-$CD107a$^+$ (**m**) and TOX$^+$CD107a$^+$ (**n**) in thymic mature SP CD8$^+$ T cells after PMA and ionomycin activation (Left). The percentage of TCF1$^-$CD107a$^+$ and TOX$^+$CD107a$^+$ in Pdcd1$^{fl/fl}$ (n=4) and Pdcd1 cKO mice (n=3) was shown (right). (**o**) Frequency of IL-2$^+$ in thymic total, semi-mature and mature SP CD8$^+$ T cells following PMA and ionomycin stimulation in Pdcd1$^{fl/fl}$ (n=3) and Pdcd1 cKO (n=5) mice. (**a-b**) was created in BioRender. Mao, Z. (2026) https://BioRender.com/272jcun Each point represents one mouse, with two to three biological replicates averaged per mouse prior to statistical analysis. Data are presented as mean ± SEM. Statistical analyses were performed at the mouse level using unpaired two-tailed t-tests.

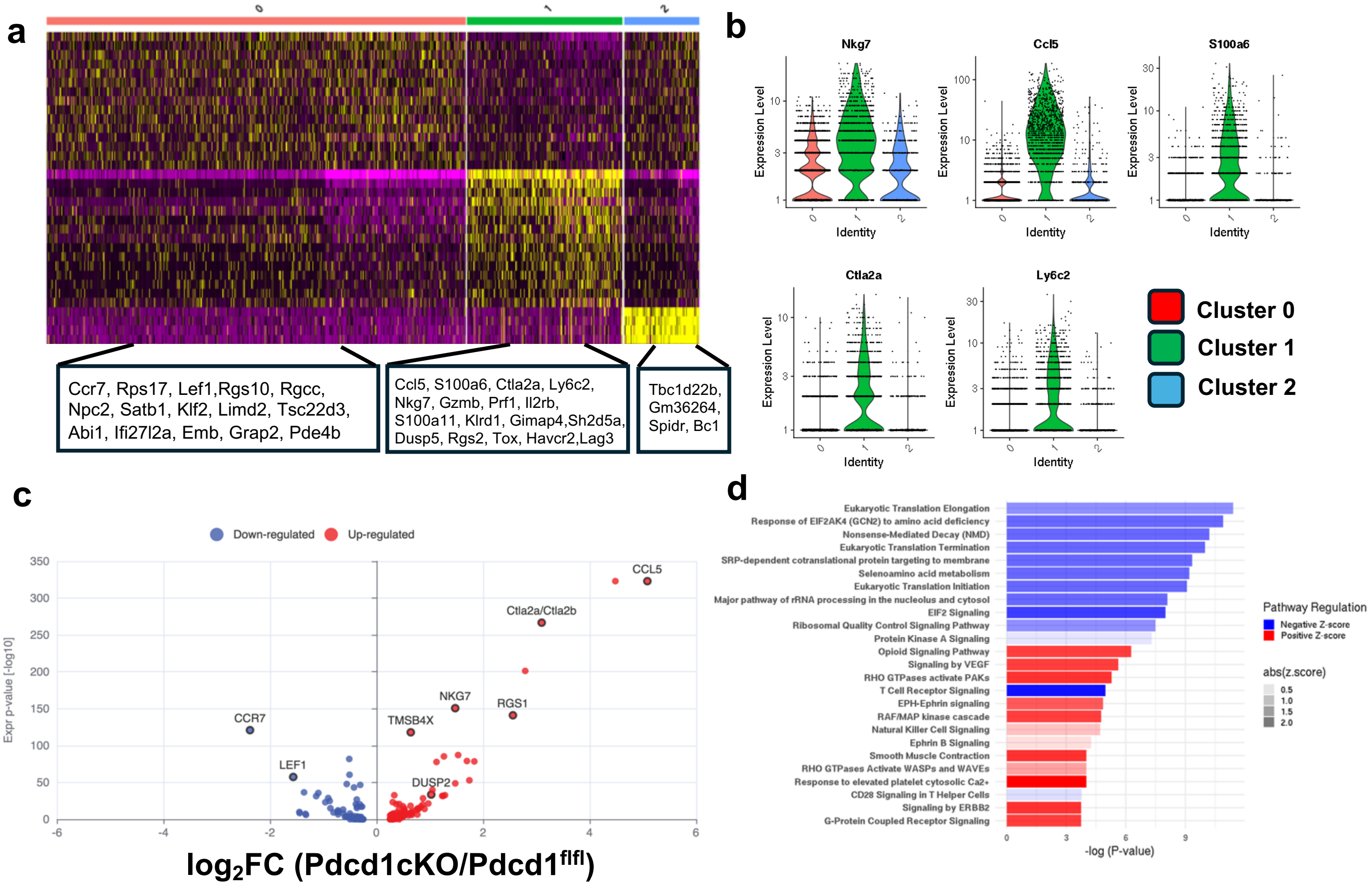

Supplementary Figure 2. Single cell RNA-seq analysis of thymic CD8^{+} T cells. (a) Heatmap of differential gene expression across three identified clusters in thymic CD8^{+} T cells shown in **Fig 1(b)**. Yellow represents upregulated genes, while purple represents downregulated genes within each cluster. (**b**) Violin plots illustrate the expression levels of signature top candidate genes within cluster 1 across the three clusters in **Fig 1(b)**. (**c**) Differential Gene Expression (DGE) of cluster 1 in CD8-Pdcd1 cKO mice compared with control. Red: genes upregulated in Pdcd1 cKO mouse. Blue: genes downregulated in Pdcd1 cKO mouse. (**d**) Ingenuity pathway analysis (IPA) on DGE in cluster 1. Positive Z score indicates the gene set more likely to activate the pathway. Negative Z score indicates the gene set more likely to inhibit the pathway. The density of color indicates the absolute values of Z score.

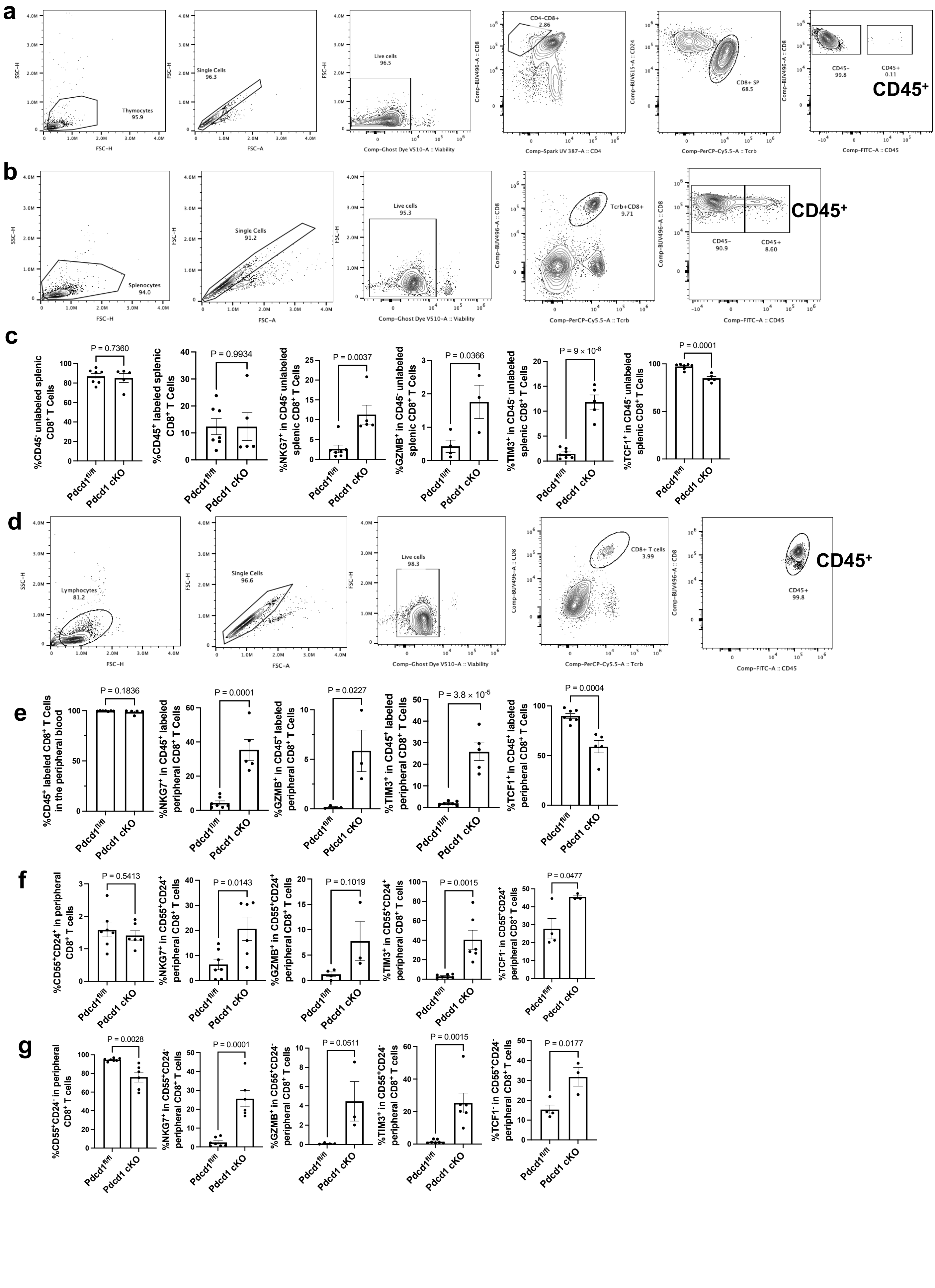
a
Thymocytes 95.9
Single Cells 96.3
Live cells 96.5
CD4-CD8+ 2.86
CD8+ SP 68.5
CD45- 99.8
CD45+ 0.11
CD45⁺
FSC-H
SSC-H
FSC-A
Comp-Ghost Dye V510-A :: Viability
Comp-Spark UV 387-A :: CD4
Comp-BUV496-A :: CD8
Comp-BUV615-A :: CD24
Comp-PerCP-Cy5.5-A :: Tcrb
Comp-FITC-A :: CD45
b
Splenocytes 94.0
Single Cells 91.2
Live cells 95.3
Tcrb+CD8+ 9.71
CD45- 90.9
CD45+ 8.60
CD45⁺
c
P = 0.7360
%CD45⁻ unlabeled splenic CD8⁺ T Cells
P = 0.9934
%CD45⁺ labeled splenic CD8⁺ T Cells
P = 0.0037
%NKG7⁺ in CD45⁻ unlabeled splenic CD8⁺ T Cells
P = 0.0366
%GZMB⁺ in CD45⁻ unlabeled splenic CD8⁺ T Cells
P = 9 × 10⁻⁶
%TIM3⁺ in CD45⁻ unlabeled splenic CD8⁺ T Cells
P = 0.0001
%TCF1⁺ in CD45⁻ unlabeled splenic CD8⁺ T Cells
Pdcd1fl/fl
Pdcd1 cKO
d
Lymphocytes 81.2
Single Cells 96.6
Live cells 98.3
CD8+ T cells 3.99
CD45+ 99.8
CD45⁺
e
P = 0.1836
%CD45⁺ labeled CD8⁺ T Cells in the peripheral blood
P = 0.0001
%NKG7⁺ in CD45⁺ labeled peripheral CD8⁺ T Cells
P = 0.0227
%GZMB⁺ in CD45⁺ labeled peripheral CD8⁺ T Cells
P = 3.8 × 10⁻⁵
%TIM3⁺ in CD45⁺ labeled peripheral CD8⁺ T Cells
P = 0.0004
%TCF1⁺ in CD45⁺ labeled peripheral CD8⁺ T Cells
f
P = 0.5413
%CD55⁺CD24⁺ in peripheral CD8⁺ T cells
P = 0.0143
%NKG7⁺ in CD55⁺CD24⁺ peripheral CD8⁺ T cells
P = 0.1019
%GZMB⁺ in CD55⁺CD24⁺ peripheral CD8⁺ T cells
P = 0.0015
%TIM3⁺ in CD55⁺CD24⁺ peripheral CD8⁺ T cells
P = 0.0477
%TCF1⁻ in CD55⁺CD24⁺ peripheral CD8⁺ T cells
g
P = 0.0028
%CD55⁺CD24⁻ in peripheral CD8⁺ T cells
P = 0.0001
%NKG7⁺ in CD55⁺CD24⁻ peripheral CD8⁺ T cells
P = 0.0511
%GZMB⁺ in CD55⁺CD24⁻ peripheral CD8⁺ T cells
P = 0.0015
%TIM3⁺ in CD55⁺CD24⁻ peripheral CD8⁺ T cells
P = 0.0177
%TCF1⁻ in CD55⁺CD24⁻ peripheral CD8⁺ T cells

Supplementary Figure 3. Evaluation of circulating and non-circulating CD8$^+$ T cells in thymus and spleen at baseline. (a-d) CD45$^+$ Intravenous labeling of circulating, peripheral CD8$^+$ T cells. **(a, b, d)** Flow cytometry gating strategy of CD45$^+$ CD8$^+$ T cells in thymus **(a)**, spleen **(b)** and peripheral blood **(d)**. (**c, e)** Percentage of NKG7, GZMB, TIM3 and TCF-1 in non-circulating CD45$^-$ spleen (**c**) and circulating CD45$^+$ peripheral blood CD8$^+$ T cells (**e**) in Pdcd1$^{fl/fl}$ (n=7) and Pdcd1 cKO (n=5) mice. (**f**) Frequencies of CD55$^+$CD24$^+$ recent thymic emigrants (RTE) in peripheral blood CD8$^+$ T cells and the percentages of NKG7, GZMB, TIM3 and TCF1 in CD55$^+$CD24$^+$ CD8$^+$ T cells in Pdcd1$^{fl/fl}$ and Pdcd1 cKO mice. (**g**) Frequency of CD55$^+$CD24$^-$ mature peripheral CD8$^+$ T cells and the percentages of NKG7, GZMB, TIM3 and TCF1 in CD55$^+$CD24$^-$ CD8$^+$ T cells in Pdcd1$^{fl/fl}$ and Pdcd1 cKO mice. Each point represents one mouse, with two to three biological replicates averaged per mouse prior to statistical analysis. Data are presented as mean ± SEM. Statistical analyses were performed at the mouse level using unpaired two-tailed t-tests.

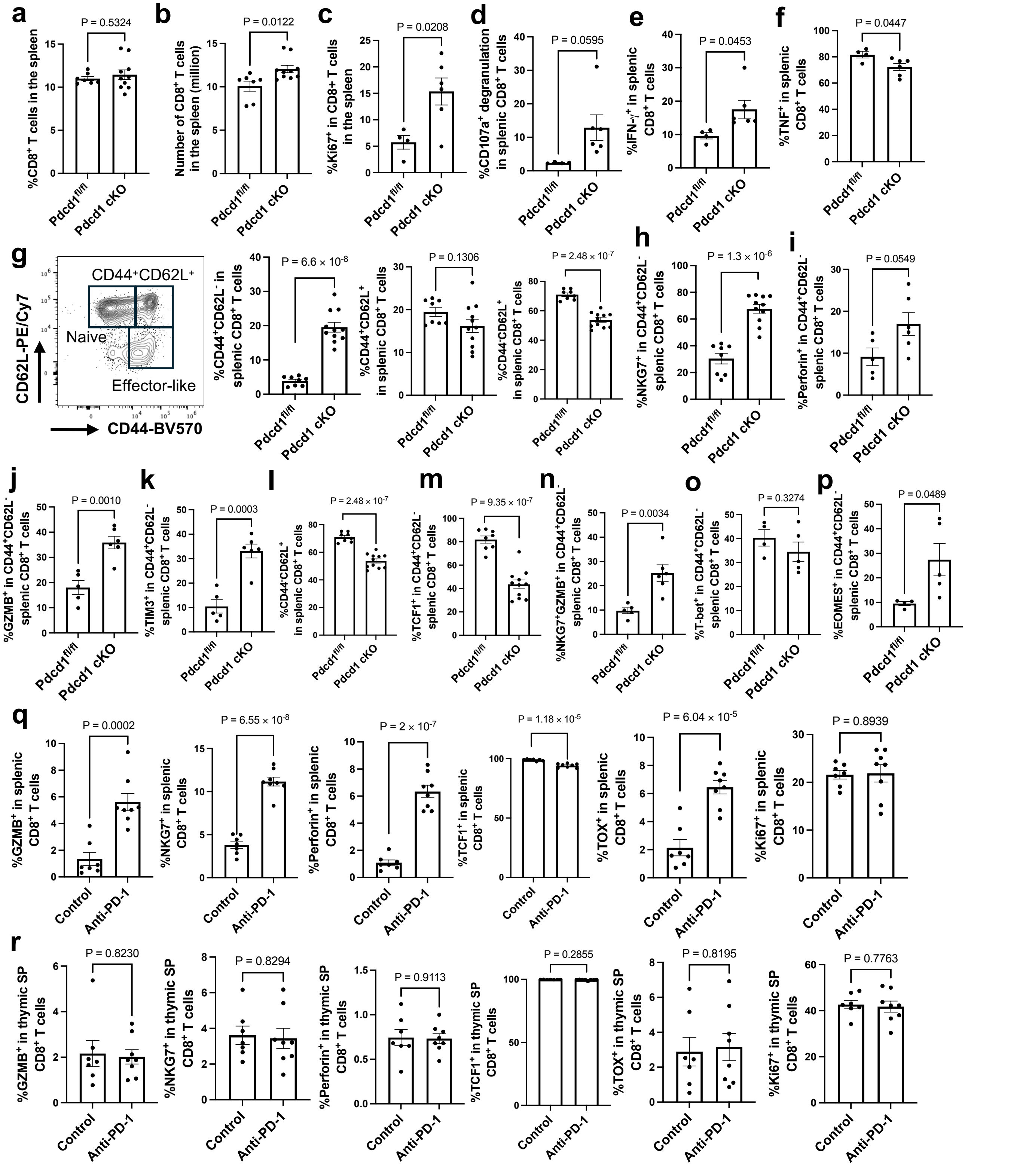

Supplementary Figure 4. Increased cytotoxic potential of peripheral effector CD8⁺ cells in the absence of PD-1. (a-c) Frequencies, absolute numbers, and proliferative capacity of splenic CD8⁺ T cells in Pdcd1$^{fl/fl}$ (n=7) and Pdcd1 cKO (n=10) mice. **(a)** Percentage of CD8⁺ T cells in the spleen. **(b)** Total number of splenic CD8⁺ T cells. **(c)** Percentage of Ki67⁺ CD8⁺ T cells in the spleen. **(d-f)** Degranulation and cytokine production of splenic CD8⁺ T cells following PMA and ionomycin stimulation for 4 hours in Pdcd1$^{fl/fl}$ (n=4) and Pdcd1 cKO (n=6) mice. Percentage of CD107a⁺ **(d)**, IFN-γ⁺ **(e)**, and TNF⁺ **(f)** splenic CD8⁺ T cells. **(g)** Phenotypic characterization of splenic CD8⁺ T cells. Gating strategy (left) and frequencies (right) of CD44⁺CD62L⁺, CD44⁺CD62L⁻ (effector-like) and CD44⁻CD62L⁺ (naive) among TCRβ⁺CD8⁺ T cells in the spleen of Pdcd1$^{fl/fl}$ (n=8) and Pdcd1 cKO (n=11)

mice. **(h-p)** Cytotoxic-associated markers and transcription factor expression profiles in effector-like $CD44^+CD62L^-$ splenic $CD8^+$ T cells of $Pdcd1^{fl/fl}$ and Pdcd1 cKO mice. Frequencies of $NKG7^+$ **(h,** n=8 and n=11**)**, $Perforin^+$ **(i,** n=5 and n=6**)**, Granzyme B^+ **(j,** n=5 and n=6**)**, $TIM3^+$ **(k,** n=5 and n=6**)**, TOX^+ **(l,** n=5 and n=6**)**, TCF-1^+ **(m,** n=8 and n=11**)**, $NKG7^+GZMB^+$ **(n,** n=5 and n=6**)**, T-bet^+ **(o,** n=4 and n=5**)** and $EOMES^+$ **(p,** n=4 and n=5**)** splenic $CD8^+$ T cells. (**q-r**) Impact of peripheral anti-PD-1 blockade on thymic SP $CD8^+$ T cells in naïve mice. Frequencies of $GZMB^+$, $NKG7^+$, $Perforin^+$, $TCF1^+$, TOX^+ and $Ki67^+$ in splenic CD8+ T cells (**q**) and thymic SP $CD8^+$ T cells (**r**) with or without 5 doses of 300 μg anti-PD-1 blockade every other day (day 1, 3, 5, 7, 9). Each point represents one mouse, with two to three technical replicates averaged per mouse prior to statistical analysis. Data are presented as mean ± SEM. Statistical analyses were performed at the mouse level using unpaired two-tailed t-tests.

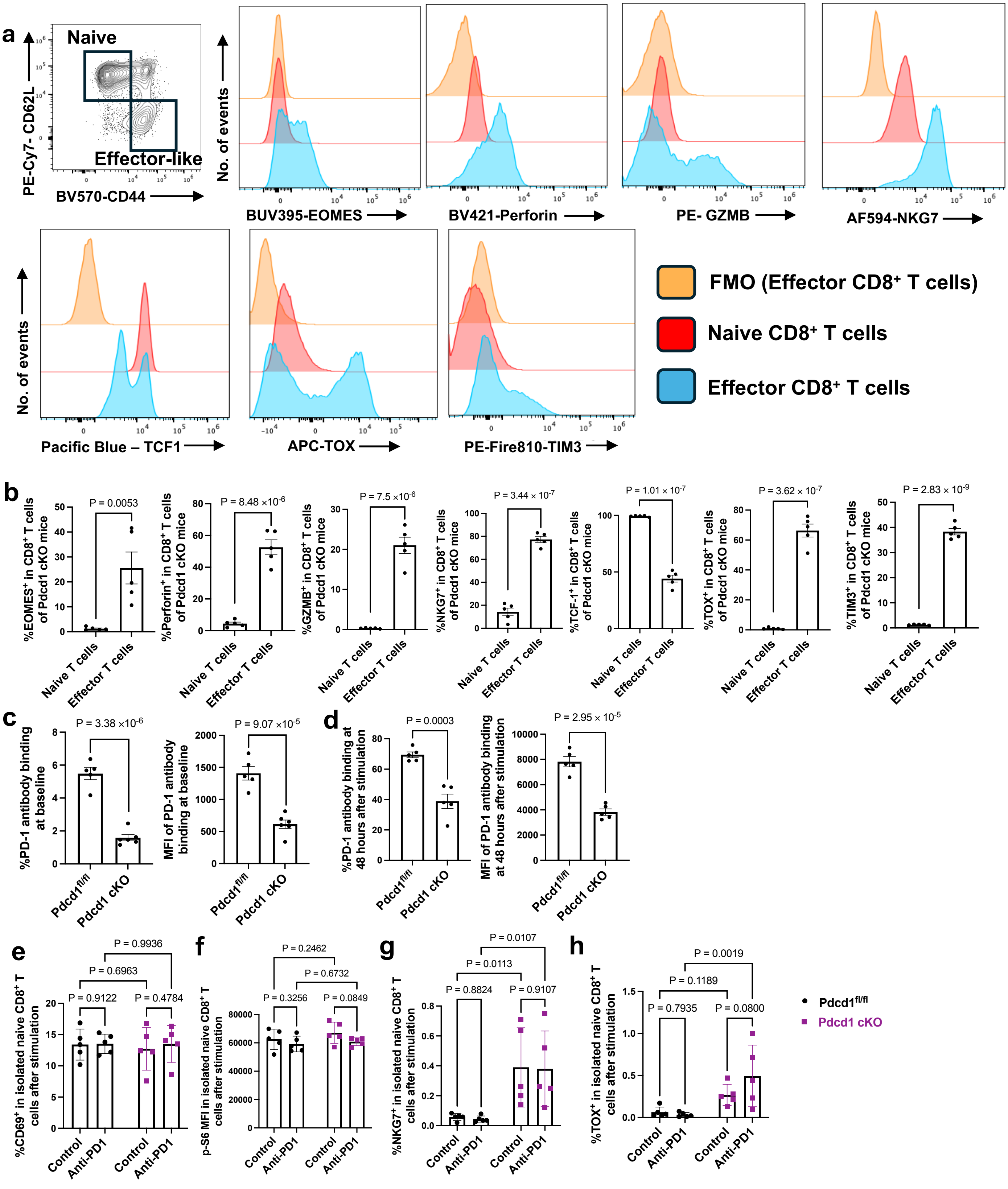

Supplementary Figure 5. Acute PD-1 blockade does not recapitulate latent effector differentiation observed in Pdcd1 cKO mice. (a-b) Cytotoxic effector marker expression in naïve T cells and effector-like splenic CD8⁺ T cells at baseline in the absence of PD-1. **(a)** Contour plot showing naïve and effector-like splenic CD8+ T cells followed by histograms showing the protein expression of EOMES, Perforin, GZMB, NKG7, TCF1, TOX and TIM3 in these cells using FMO as control in Pdcd1 cKO mice (n=5). (**b**) Frequencies of EOMES⁺, Perforin⁺, GZMB⁺, NKG7⁺, TCF1⁺, TOX⁺ and TIM3⁺ in naïve CD8⁺ T cells and effector-like CD8⁺ T cells in Pdcd1

cKO mice (n=5). (**c-d**) Pharmacodynamic PD-1 binding target engagement assay. Splenic CD8$^+$ T cells from Pdcd1$^{fl/fl}$ (n=5) and Pdcd1 cKO (n=5) were incubated with Hamster anti-PD-1 antibody (Clone: G4) at baseline (**c**) or following 24h anti-CD3/CD28 stimulation (**d**), followed by staining with Alexa Fluor 555-conjugated anti-Hamster secondary antibody. Frequencies (Left) and MFI (right) of AF555$^+$ cells indicating PD-1 antibody binding are shown at baseline (**c**) and after stimulation (**d**). (**e-h**) Evaluation of activation and cytotoxic effector protein expression following acute PD-1 blockade. Splenic naïve CD8$^+$ T cells from Pdcd1$^{fl/fl}$ (n=5) and Pdcd1 cKO (n=5) mice were isolated and stimulated with anti-CD3/CD28 stimulation for 24 hours, with or without addition of 20 μg/mL anti-PD-1 antibody. Frequencies of CD69$^+$ (**e**) NKG7$^+$(**g**), TOX$^+$ (**h**), and MFI of p-S6 (**f**) are shown for stimulated naïve CD8$^+$ T cells. Each dot represents one biologically independent mouse, with two biological repeats averaged per mouse prior to statistical analysis. Data are presented as mean ± SEM. Statistical analysis was performed using repeated measures two-way ANOVA followed by uncorrected Fisher's LSD with a single pooled variance.

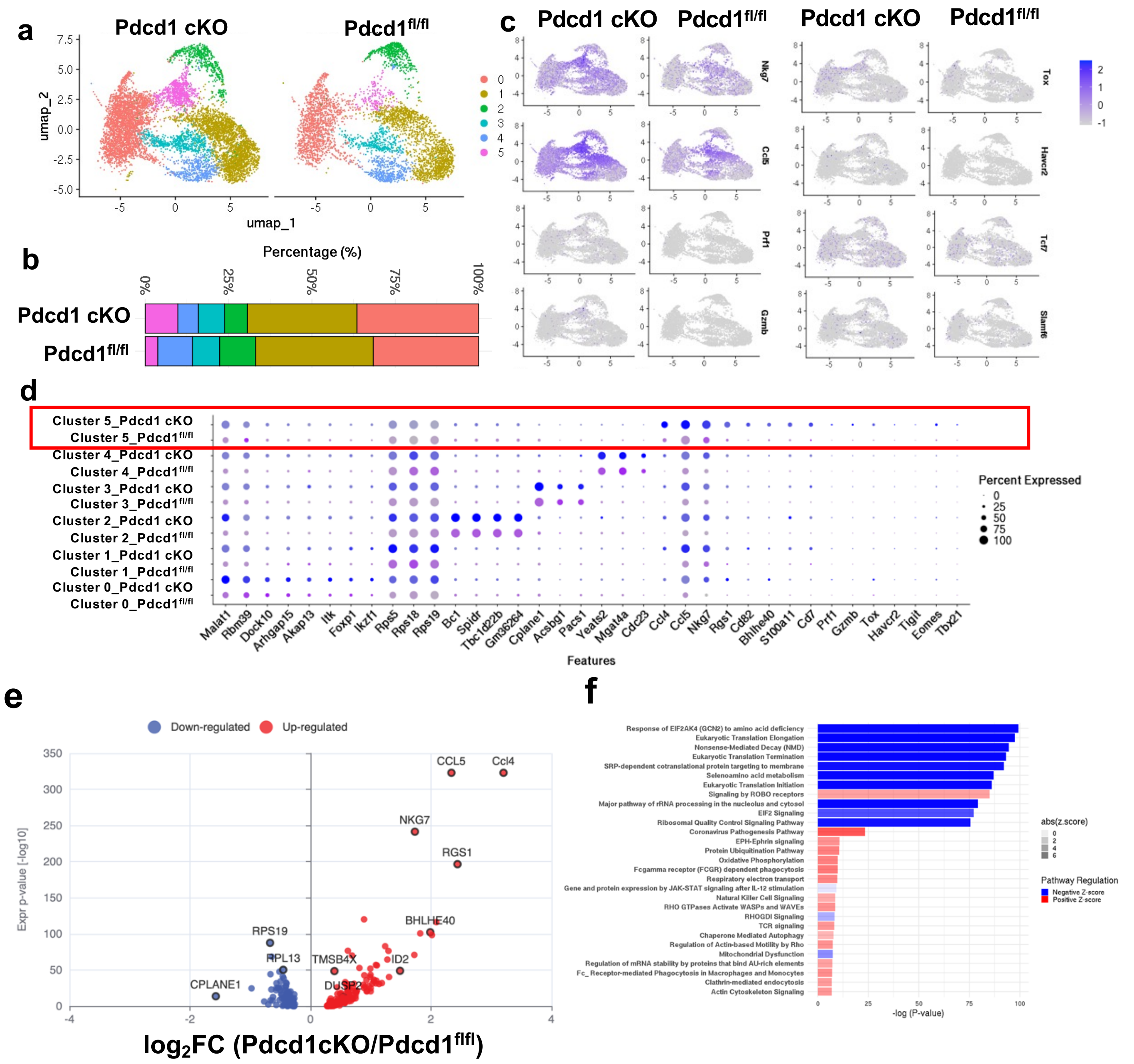

Supplementary Figure 6. Single cell RNA-seq analysis of splenic CD8⁺ T cells. **(a)** UMAP visualization of splenic CD8⁺ T cell clusters in from one Pdcd1 cKO (left) and one Pdcd1$^{fl/fl}$ (right) mouse. **(b)** Distribution of cluster frequencies within splenic CD8⁺ T cells. (**c**) Feature plots showing the UMAP distribution and normalized expression of *Nkg7, Ccl5, Prf1*, *Gzmb*, *Tox*, *Havcr2*, *Tcf7*, and *Slamf6* in splenic CD8⁺ T cells of Pdcd1 cKO (left) and Pdcd1$^{fl/fl}$ (right) mice. Gene expression is depicted by a gradient, with darker blue representing higher expression levels. **(d)** Dotplot showing the differential gene expression within each cluster and selected genes associated with cytotoxic potential stratified by Pdcd1 cKO and Pdcd1$^{fl/fl}$ CD8⁺ T cells. (**e**) DGE of cluster 5 effector cells in CD8-Pdcd1 cKO mice compared with control. Red: genes upregulated in Pdcd1 cKO mouse. Blue: genes downregulated in Pdcd1 cKO mouse. (**f**) IPA on DGE of cluster 5. Positive Z score indicates the gene set more likely to activate the pathway. Negative Z score indicates the gene set more likely to inhibit the pathway. The density of color indicates the absolute values of Z score. Differential expression testing was performed using the Wilcoxon rank-sum test as implemented in Seurat, with Benjamini–Hochberg correction for multiple testing. Complete differential gene expression list is shown in **Supplementary Table 2**.

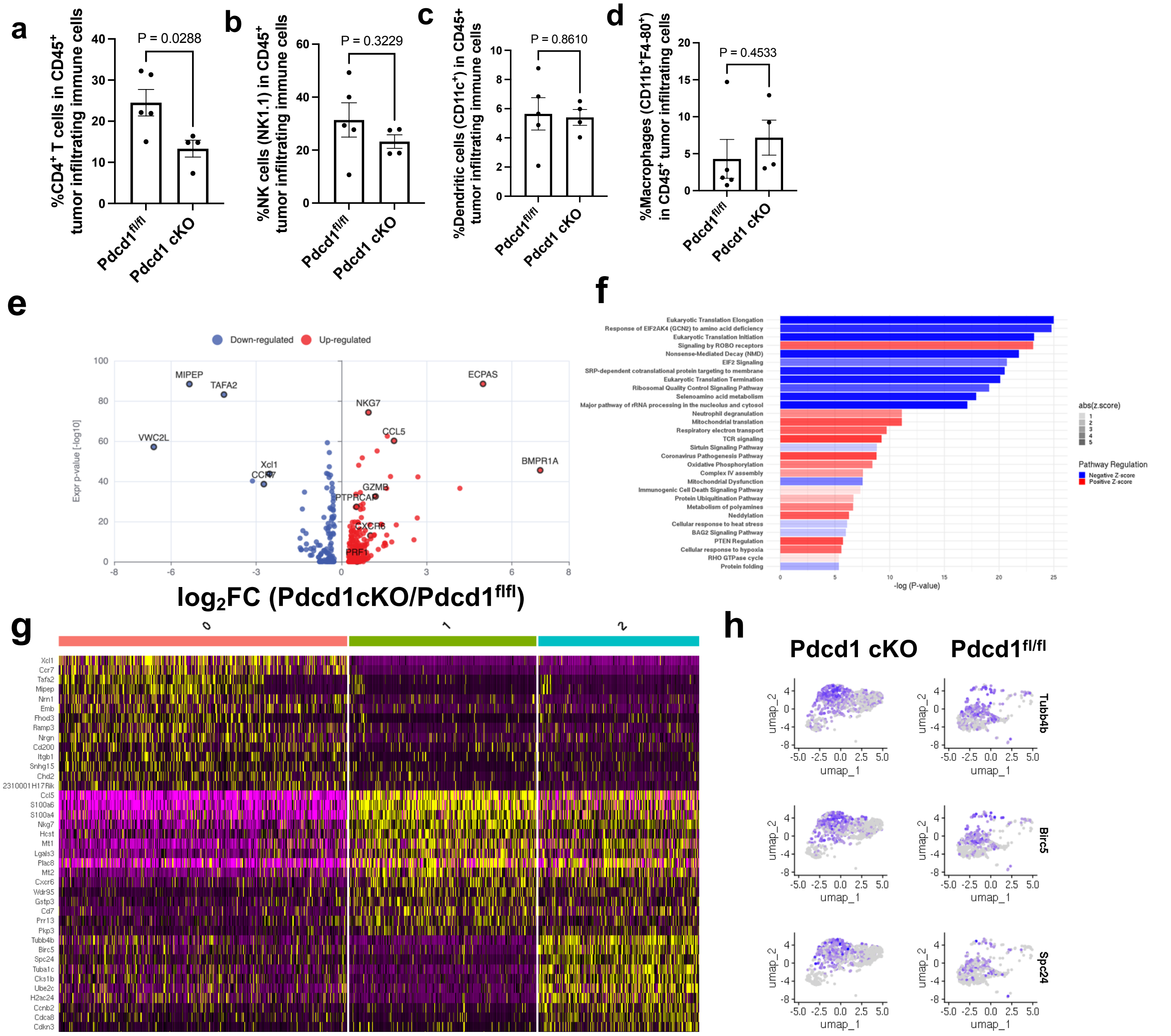

Supplementary Figure 7. Single cell RNA-seq analysis of CD8$^+$ TILs. (**a-d**) Frequencies of TCRβ$^+$CD4$^+$ T cells (CD4$^+$ T cells, **a**), NK1.1$^+$ (NK cells, **b**), CD11c$^+$ (Dendritic cells, **c**) and TCRβ$^-$CD11b$^+$F4-80$^+$ (macrophages, **d**) in CD45$^+$ tumor infiltrating immune cells) in Pdcd1$^{fl/fl}$ (n=5) and Pdcd1 cKO (n=4) mice. (**e**) DGE of CD8$^+$ TILs in pooled Pdcd1 cKO mice (n=3) compared with control (n=3). Red: genes upregulated in Pdcd1 cKO mouse. Blue: genes downregulated in Pdcd1 cKO mouse. (**f**) IPA analysis of DGE of CD8$^+$ TILs. Red: genes upregulated in Pdcd1 cKO mouse. Blue: genes downregulated in Pdcd1 cKO mouse. Positive Z score indicates the gene set more likely to activate the pathway. Negative Z score indicates the gene set more likely to inhibit the pathway. The density of color indicates the absolute values of Z score. (**g**) Heatmap of the differential gene expression of each sub-cluster identified in the UMAP of CD8$^+$ TILs in Fig. 4C**. (h)** Feature plots show the UMAP distribution and expression levels of other featured genes that were only upregulated in cluster 1 stratified by CD8$^+$ TILs from control and Pdcd1 cKO mice. Each point represents one mouse, with two biological replicates averaged per mouse prior to statistical analysis. Data are presented as mean ± SEM. Statistical analyses were performed at the mouse level using unpaired two-tailed t-tests. Differential expression testing was performed using the Wilcoxon rank-sum test as implemented in Seurat, with Benjamini–Hochberg correction for multiple testing.

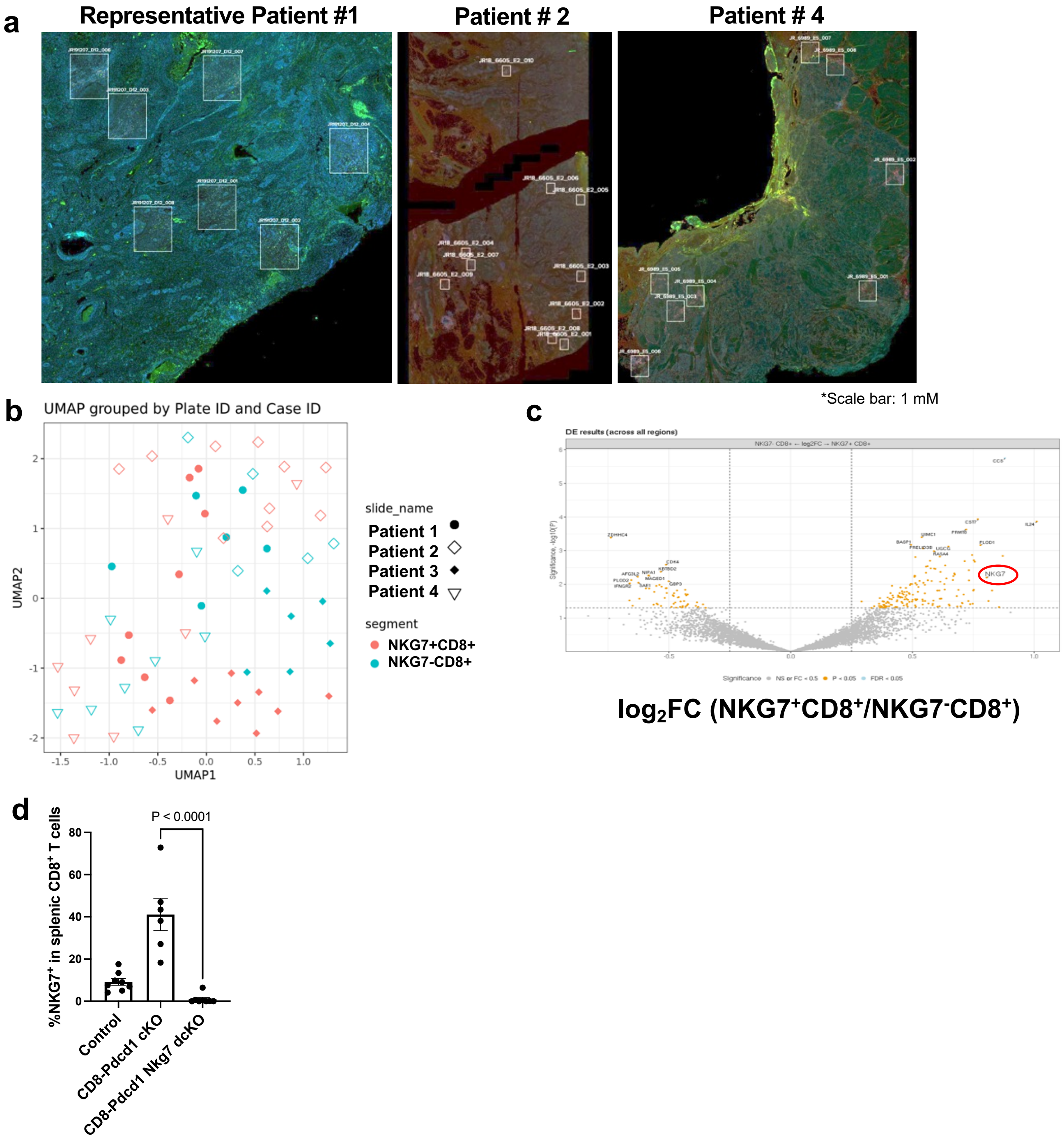

Supplementary Figure 8. Spatial transcriptome analysis of NKG7 expressing $CD8^+$ T cells in human tumor tissues. (**a**) Region of interest selected in tumor slides of other three patients with invasive bladder cancer for spatial transcriptomics in addition to the representative **Fig. 4f. (b)** UMAP graph shows the principal component-based dimensionality reduction of the four patient tumor tissue samples, highlighting distinct clustering of $NKG7^+CD8^+$ vs. $NKG7^-CD8^+$ T cells. **(c)** DGE comparing $NKG7^+CD8^+$ T cells vs. $NKG7^-CD8^+$ T cells, shown as log_2fold change (log_2FC). NKG7 expression is highlighted in the volcano plot. (**d**) The frequency of $NKG7^+$ in splenic CD8+ T cells of CD8-Pdcd1 Nkg7 dcKO (n=8) compared with CD8-Pdcd1 cKO (n=6) and control mice (n=8) at the end point post B16-OVA tumor challenge. Each dot in (**b**) represents a segmented region within ROI from pooled four independent patients. Data from four biologically independent patients were analyzed. Differential expression testing was performed using patient-level aggregation, with false discovery rate (FDR) correction applied for multiple comparisons. Each dot in (**d**) represents one mouse, with three technical

replicates averaged per mouse prior to statistical analysis. Data are presented as mean ± SEM and p value was calculated using unpaired two-tailed t-tests.

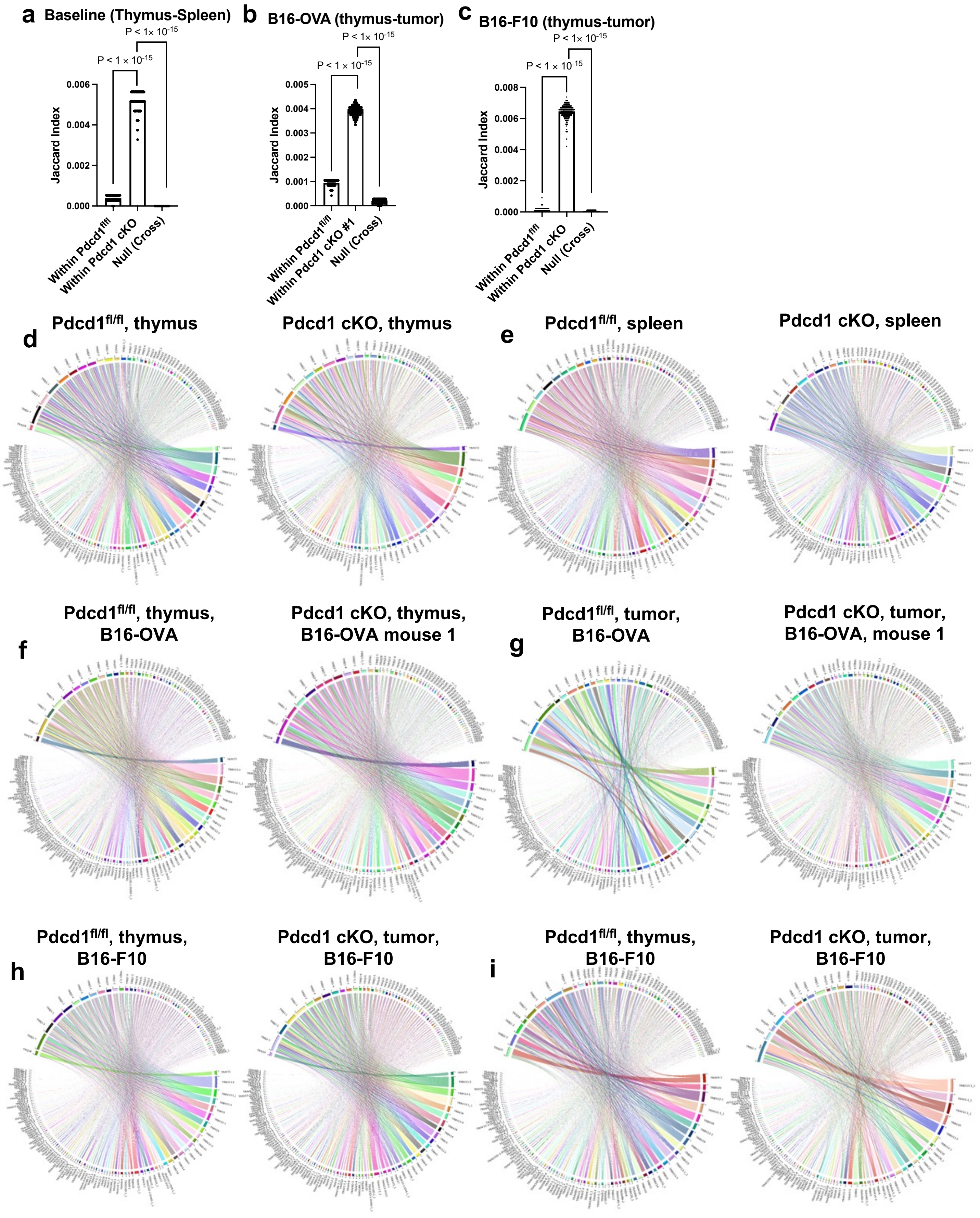
a
Baseline (Thymus-Spleen)
P < 1× 10-15
P < 1 × 10-15
Jaccard Index
0.006
0.004
0.002
0.000
Within Pdcd1fl/fl
Within Pdcd1 cKO
Null (Cross)
b
B16-OVA (thymus-tumor)
P < 1× 10-15
P < 1 × 10-15
Jaccard Index
0.005
0.004
0.003
0.002
0.001
0.000
Within Pdcd1fl/fl
Within Pdcd1 cKO #1
Null (Cross)
c
B16-F10 (thymus-tumor)
P < 1× 10-15
P < 1 × 10-15
Jaccard Index
0.008
0.006
0.004
0.002
0.000
Within Pdcd1fl/fl
Within Pdcd1 cKO
Null (Cross)
d
Pdcd1fl/fl, thymus
Pdcd1 cKO, thymus
e
Pdcd1fl/fl, spleen
Pdcd1 cKO, spleen
f
Pdcd1fl/fl, thymus, B16-OVA
Pdcd1 cKO, thymus, B16-OVA mouse 1
g
Pdcd1fl/fl, tumor, B16-OVA
Pdcd1 cKO, tumor, B16-OVA, mouse 1
h
Pdcd1fl/fl, thymus, B16-F10
Pdcd1 cKO, tumor, B16-F10
i
Pdcd1fl/fl, thymus, B16-F10
Pdcd1 cKO, tumor, B16-F10

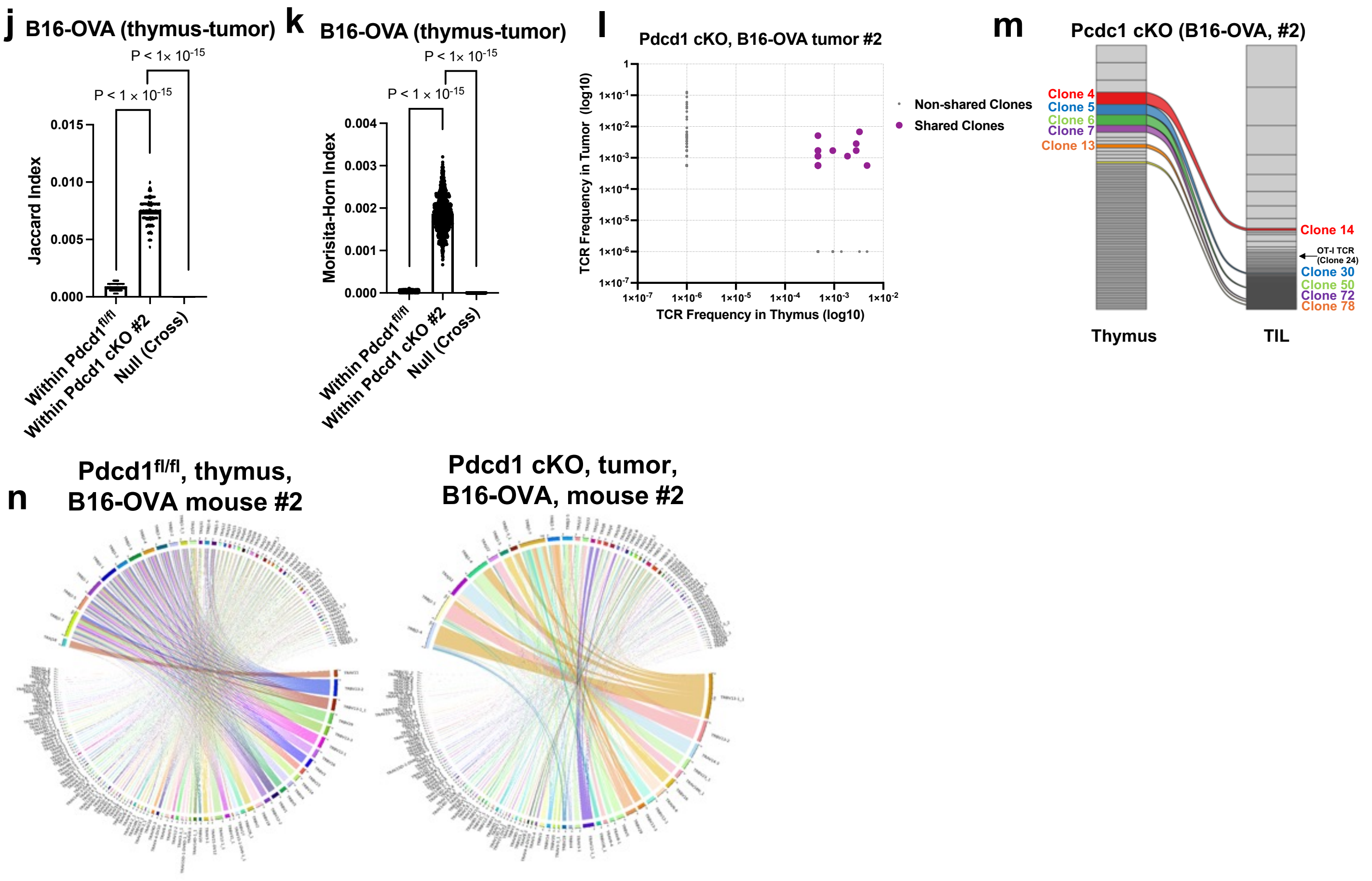

Supplementary Figure 9. TCR landscape analysis in thymus and spleen at baseline and in thymus and tumor after tumor growth. (a-c) Jaccard index quantifying TCR repertoire overlap after rarefaction as described in **Fig. 5**, benchmarked against a cross-mouse null model. Overlap between thymus and spleen at baseline (**g**), and between thymus and tumor in mice bearing B16-OVA tumors (**h**) or B16-F10 tumors (**I**) 12 days post tumor injection. V(D)J rearrangements of TCR repertoire in the thymus (**d**) and spleen (**e**) of Pdcd1$^{fl/fl}$ and Pdcd1 cKO mice baseline. V(D)J rearrangements in the thymus (**f**) and tumor (**g**) of Pdcd1$^{fl/fl}$ and Pdcd1 cKO mice at day 12 post B16-OVA tumor injection. (**h, i**) V(D)J rearrangements in the thymus and tumor at day 12 after B16-F10 tumor injection in Pdcd1$^{fl/fl}$ (**H**) and Pdcd1 cKO mice (**I**). **(j-n)** TCR overlapping analysis for a second Pdcd1 cKO mouse bearing B16-OVA tumor. Jaccard index (**j**) and Morisita-Horn index (**k**) quantifying overlap in thymus-tumor overlap after rarefication, benchmarked against the null model. (**l**) Clonal frequency scatter plots of productive TCR clonotype frequencies in thymus versus B16-OVA TILs from a second independent Pdcd1 cKO mice after depth-aware normalization. **(m)** Sankey diagrams illustrating selected overlapping productive clonotypes between thymus and B16-OVA TILs from the original (unrarefied) top 100 productive clonotypes. (**n**) V(D)J rearrangements in the thymus and tumor of a second CD8-Pdcd1 cKO mouse following B16-OVA tumor injection. For (**a-c, j-l**), each dot represents one rarefaction iteration (1,000 iterations per biological sample pair). Rarefaction was used to estimate the sampling distribution of overlap metrics while controlling for sequencing depth and clonality. Statistical comparisons were performed on iteration-derived distributions within each biological sample pair relative to the null model; the biological unit of analysis is the mouse/sample pair, not the rarefaction iteration.

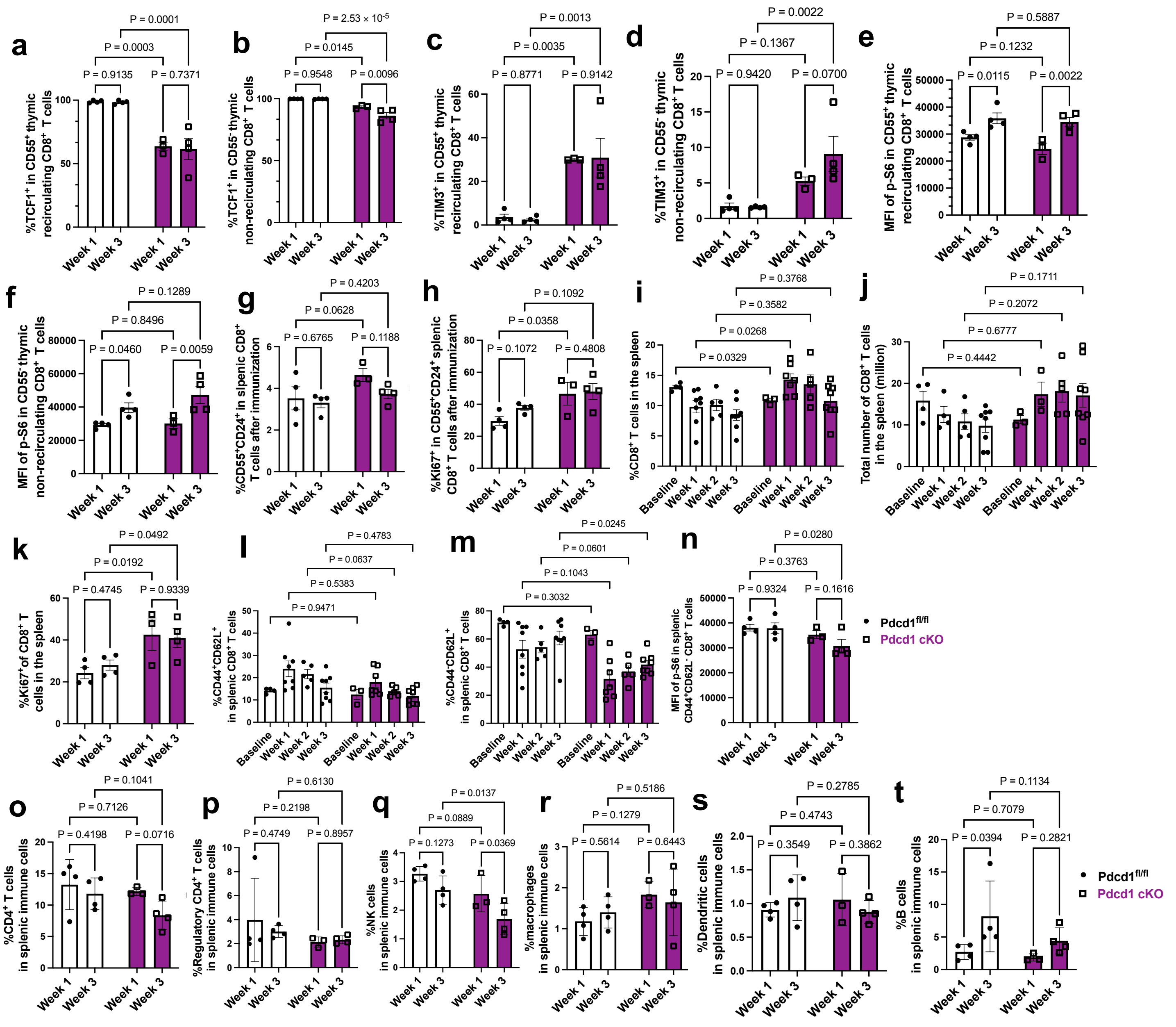

Supplementary Figure 10. Thymus and spleen phenotype of CD8$^+$ T cells after poly(I:C)/OVA immunization. **(a, b)** Frequencies of TCF1$^+$ in CD55$^+$ thymic recirculating-like CD8$^+$ T cells **(a)** and CD55$^-$ thymic non-recirculating **(b)** CD8$^+$ T cells at 1-week (n=4 and n=3) and 3-week (n=4 and n=3) after poly(I:C)/OVA immunization in Pdcd1$^{fl/fl}$ and Pdcd1 cKO mice. **(c, d)** Frequencies of TIM3$^+$ in CD55$^+$ thymic recirculating-like CD8$^+$ T cells **(c)** and CD55$^-$ thymic non-recirculating **(d)** CD8$^+$ T cells at 1-week and 3-week after poly(I:C)/OVA immunization. (**e, f**) Median fluorescent intensity (MFI) of p-S6 in CD55$^+$ thymic recirculating-like **(e)** and CD55$^-$ non-recirculating **(f)** CD8+ T cells at 1-week and 3-week after poly(I:C)/OVA immunization. (**g**) Frequencies of CD55$^+$CD24$^+$ RTE in splenic CD8+ T cells after poly(I:C)/OVA immunization. **(h)** Percentage of Ki67$^+$ in CD55$^+$CD24$^+$ splenic CD8$^+$ T cells after poly(I:C)/OVA immunization. (**i**) Percentage of splenic CD8+ T cells at baseline (n=4 and n=3) and following 1-week (n=8 and n=7), 2-week (n=5 and n=5) and 3-week (n=8 and n=8) poly(I:C)/OVA immunization in Pdcd1$^{fl/fl}$ and Pdcd1 cKO mice. (**j**) Total number count of splenic CD8+ T cells at baseline (n=4 and n=3) and following 1-week (n=4 and n=3), 2-week (n=5 and n=5) and 3-week (n=8 and n=8) poly(I:C)/OVA immunization in Pdcd1$^{fl/fl}$ and Pdcd1 cKO mice. (**k**) Frequency of Ki67$^+$ of splenic CD8+ T cells at 1-week (n=4 and n=3) and 3-week (n=4 and n=4) after immunization. (**l, m**) Percentage of CD44$^+$CD62L$^-$ (**l**) and CD44$^-$CD62L$^+$ (**m**) CD8$^+$ T cells at baseline and following 1-week (n=8 and n=7), 2-week (n=5 and n=5) and 3-week (n=8 and n=8) immunization in Pdcd1$^{fl/fl}$ and Pdcd1 cKO mice. (**n**) MFI of p-S6 in splenic effector-like

$CD44^+CD62L^-$ splenic $CD8^+$ T cells after immunization. (**o-t**) Frequencies of $CD4^+$ T cells (**o**), $Foxp3^+$ regulatory $CD4^+$ T cells (**p**), NK cells (**q**), $TCR\beta^-CD11b^+F4\text{-}80^+$ macrophages (**r**), $TCR\beta^-CD11c^+$dendritic cells (**s**), $CD19^+$B cells (**t**) in splenic $CD45^+$ immune cells after immunization. Each dot represents one biologically independent mouse. Data were pooled from two to three independent experiments. For (**i-j, l-m**), statistical analysis was performed using a mixed-effects model with Geisser-Greenhouse correction, followed by Sidak's multiple comparison test. Other statistical analysis was performed using a mixed-effects model, followed by uncorrected Fisher's LSD *post hoc* test without correction for multiple comparisons (single pooled variance). Source data are provided as a Source Data file.

Supplementary Table 1. Flow cytometry reagents

Reagent	Conjugate/Channel	Clone	Vendor	Catalog number	Dilutions
**In vivo labeling reagents**					
CD45	FITC	30-F11	Biolegend	103118	
**Viability Dye**					
Zombie UV	UV 355 nm	NA	Biolegend	423108	1 to 1000
Ghost Dye V510	Violet (405 nm)	NA	Cytek Biosciences	SKU 13-0870-T100	1 to 1000
**Thymocytes in vitro killing assay**					
CD3	NA	145-2C11	Thermofisher	16-0031-82	
**Mitochondrial Superoxide Indicators**					
MitoSOX	Red ~396/610 nm	NA	Thermofisher	M36008	
**Cell Surface Staining**					
TCRβ	PerCP-Cy5.5	H57-597	Biolegend	109227	1 to 200
CD4	Spark UV387	GK1.5	Biolegend	100492	1 to 200
CD8	BUV496	53-6.7	BD Biosciences	569181	1 to 200
CD8	BV510	53-6.7	Biolegend	100752	1 to 200
CD25	BUV563	PC61.5	Thermofisher	365-0251-82	1 to 100
CD24	BUV615	M1/69	BD Biosciences	751499	1 to 200
CD11a	BV711	M1/4	BD Biosciences	740676	1 to 200
CD44	BV570	IM7	Biolegend	103037	1 to 100
CD62L	PE-Cy7	MEL-14	Biolegend	104418	1 to 100
PD-1	APC-Cy7	29F.1A12	Biolegend	153224	1 to 100
CD69	BV785	H1.2F3	Biolegend	104543	1 to 100
CD117	BV750	2B8	BD Biosciences	747412	1 to 100
CX3CR1	BV650	SA011F11	Biolegend	149033	1 to 100
CD127	BUV737	SB/199	BD Biosciences	612841	1 to 100
CD11b	BUV805	M1/70	eBiosciences	368-0112-82	1 to 100
CD19	cFluor UV440	6B5	Cytek Biosciences	SKU R7-20835	1 to 100
TIM-3	PE-Fire810	RMT3-23	Biolegend	149033	1 to 100
CD11c	RB780	HL3	BD Biosciences	755338	1 to 100
CD55	PE	RIKO-3	Biolegend	131804	1 to 200
H2-Kb	APC	AF6-88.5	Biolegend	116518	1 to 200
NK1.1	PE-Fire 700	S17016D	Biolegend	156528	1 to 100
F4-80	BV785	BM8	Biolegend	123141	1 to 100
**Intracellular and cytokine staining**					
Perforin	BV421	S16009A	Biolegend	154319	1 to 100
Granzyme B	PE	QA18A28	Biolegend	396406	1 to 100
KLRG1	BUV661	2F1	BD Biosciences	741586	1 to 100
TCF1	Pacific Blue	C63D9	Cell signaling Technology	9066S	1 to 100
TOX	APC	REA473	Miltenyl Biotech	130-118-335	1 to 50
T-bet	RB545	O4-46	BD Biosciences	569253	1 to 50
EOMES	BUV395	X4-83	BD Biosciences	567171	1 to 100
CD107a	APC	1D4B	Biolegend	505809	1 to 100
IFN-γ	BV480	XMG1.2	Thermofisher	414-7311-80	1 to 100
TNF-α	RB705	MP6-XT22	BD Biosciences	570733	1 to 100
IL-2	PE-Cy5	JES6-5H4	Biolegend	503824	1 to 100
phosphorylated-S6	PE-Cy7	cupk43k	Thermofisher	25-9007-42	1 to 100
Nur77	Alexa Fluor 488	12.14	Thermofisher	53-5965-82	1 to 100
Ki67	Alexa Fluor 700	16A8	Biolegend	652420	1 to 200

Supplementary Table 2. Rarefication number per mouse for TCR sequencing

Comparison	Organ	Mouse condition	Productive clonotype Reads number	**After Rarefication**
Comparision 1. Baseline thymus-spleen	Thymus	Control Baseline	1,909	
	Spleen	Control Baseline	2,943	**1,909**
	Thymus	Pdcd1 cKO Baseline	1,097	
	Spleen	Pdcd1 cKO Baseline	1,252	**1,097**
Comparision 2. B16-OVA thymus-tumor	Thymus	Control B16-OVA	4,125	
	Tumor	Control B16-OVA	3,623	**3,623**
	Thymus	Pdcd1 cKO B16-OVA	5,557	
	Tumor	Pdcd1 cKO B16-OVA	7,558	**5,557**
Comparision 3. B16-F10 thymus-tumor	Thymus	Control B16-F10	5,901	
	Tumor	Control B16-F10	2,834	**2,834**
	Thymus	Pdcd1 cKO B16-F10	7,222	
	Tumor	Pdcd1 cKO B16-F10	5,759	**5,759**
B16-OVA thymus-tumor mouse 2	Thymus	Pdcd1 cKO B16-OVA	2,139	
	Tumor	Pdcd1 cKO B16-OVA	1,765	**1,765**
