# Supplementary material for: PD-1 regulates latent effector differentiation of thymic cytotoxic CD8+ T cells
Source: Nat Commun. 2026 May 23;17:6769. doi: 10.1038/s41467-026-73392-7 (PMC13385908; doi:10.1038/s41467-026-73392-7)
Supplement: Supplementary file 2 — Description of Additional Supplementary Files [file 41467_2026_73392_MOESM2_ESM.pdf]

Description of Additional Supplementary Files

Supplementary Data 1: Differential Gene Expression
