# Supplementary material for: PD-1 regulates latent effector differentiation of thymic cytotoxic CD8+ T cells
Source: Nat Commun. 2026 May 23;17:6769. doi: 10.1038/s41467-026-73392-7 (PMC13385908; doi:10.1038/s41467-026-73392-7)
Supplement: Supplementary file 4 — Reporting Summary [file 41467_2026_73392_MOESM4_ESM.pdf]

nature portfolio

Corresponding author(s): Haidong Dong

Last updated by author(s): Apr 16, 2026

Reporting Summary

Nature Portfolio wishes to improve the reproducibility of the work that we publish. This form provides structure for consistency and transparency in reporting. For further information on Nature Portfolio policies, see our Editorial Policies and the Editorial Policy Checklist.

Statistics

For all statistical analyses, confirm that the following items are present in the figure legend, table legend, main text, or Methods section.

n/a	Confirmed	
[ ]	[x]	The exact sample size (*n*) for each experimental group/condition, given as a discrete number and unit of measurement
[ ]	[x]	A statement on whether measurements were taken from distinct samples or whether the same sample was measured repeatedly
[ ]	[x]	The statistical test(s) used AND whether they are one- or two-sided<br>*Only common tests should be described solely by name; describe more complex techniques in the Methods section.*
[x]	[ ]	A description of all covariates tested
[ ]	[x]	A description of any assumptions or corrections, such as tests of normality and adjustment for multiple comparisons
[ ]	[x]	A full description of the statistical parameters including central tendency (e.g. means) or other basic estimates (e.g. regression coefficient) AND variation (e.g. standard deviation) or associated estimates of uncertainty (e.g. confidence intervals)
[ ]	[x]	For null hypothesis testing, the test statistic (e.g. *F*, *t*, *r*) with confidence intervals, effect sizes, degrees of freedom and *P* value noted<br>*Give P values as exact values whenever suitable.*
[x]	[ ]	For Bayesian analysis, information on the choice of priors and Markov chain Monte Carlo settings
[x]	[ ]	For hierarchical and complex designs, identification of the appropriate level for tests and full reporting of outcomes
[x]	[ ]	Estimates of effect sizes (e.g. Cohen's *d*, Pearson's *r*), indicating how they were calculated

Our web collection on statistics for biologists contains articles on many of the points above.

Software and code

Policy information about availability of computer code

Data collection	No Custom code
Data analysis	GraphPad Prism v10 (GraphPad Inc., San Diego, CA), FlowJo v.10 software (Ashland, OR 97520 USA), Fiji Java 8, R studio, Seurat, ggplot2, sankeyNetwork, Ingenuity Pathway Analysis (IPA, QIAGEN), GeoMx DSO Analysis Suite

For manuscripts utilizing custom algorithms or software that are central to the research but not yet described in published literature, software must be made available to editors and reviewers. We strongly encourage code deposition in a community repository (e.g. GitHub). See the Nature Portfolio guidelines for submitting code & software for further information.

Data

Policy information about availability of data

All manuscripts must include a data availability statement. This statement should provide the following information, where applicable:

- Accession codes, unique identifiers, or web links for publicly available datasets
- A description of any restrictions on data availability
- For clinical datasets or third party data, please ensure that the statement adheres to our policy

a data availability statement is included in the manuscript

Research involving human participants, their data, or biological material

Policy information about studies with human participants or human data. See also policy information about sex, gender (identity/presentation), and sexual orientation and race, ethnicity and racism.

Reporting on sex and gender	Patients were not stratified by sex or gender, and no sex- or gender-specific analysis or interpretations were performed.
Reporting on race, ethnicity, or other socially relevant groupings	Patients were seen at Mayo Clinic and represent the typical population treated in this region of the United States. There was no intentional selection or exclusion based on race or ethnicity, and the study population is not biased in this regard.
Population characteristics	Our database was used to collect baseline clinicpathological variables, including age, gender, metastasis and recurrent status, tumor stage, histological type, lymph node (LN) status, and clinical follow-up data.
Recruitment	Patients with muscle invasive bladder cancer were collected following radical cystectomy under an IRB-approved protocol. Samples were obtained between 1990 and 2016. Patients with advanced hepatocellular carcinoma were collected before and after combinational immunotherapy. Samples were collected between 2022 and 2024.
Ethics oversight	Mayo Clinic IRB protocol number is provided in the Material and Methods section.

Note that full information on the approval of the study protocol must also be provided in the manuscript.

Field-specific reporting

Please select the one below that is the best fit for your research. If you are not sure, read the appropriate sections before making your selection.

[x] Life sciences [ ] Behavioural & social sciences [ ] Ecological, evolutionary & environmental sciences

For a reference copy of the document with all sections, see nature.com/documents/nr-reporting-summary-flat.pdf

Life sciences study design

All studies must disclose on these points even when the disclosure is negative.

Sample size	No formal statistical method was used to predetermine sample size. Sample sizes were chosen based on prior experience with these tumour models and immunological assays, sample sizes commonly used in similar studies, expected biological variability, and practical considerations related to animal use and assay feasibility.
Data exclusions	No data was excluded in this study.
Replication	All attempts at replication were successful; the number of experimental replicates are indicated in the respective figure legends.
Randomization	Age-matched mice were randomly assigned to experimental groups. Human cystectomy specimens were obtained from our institutional Tissue Registry.
Blinding	Blinding was applied during tumor measurement and flow cytometry data analysis. For experiments that were required to know genotype and/or treatment allocations during mouse breeding, tumor injection and sample collection, blinding was not feasible.

Reporting for specific materials, systems and methods

We require information from authors about some types of materials, experimental systems and methods used in many studies. Here, indicate whether each material, system or method listed is relevant to your study. If you are not sure if a list item applies to your research, read the appropriate section before selecting a response.

Materials & experimental systems

n/a	Involved in the study	
[ ]	[x]	Antibodies
[ ]	[x]	Eukaryotic cell lines
[x]	[ ]	Palaeontology and archaeology
[ ]	[x]	Animals and other organisms
[x]	[ ]	Clinical data
[x]	[ ]	Dual use research of concern
[x]	[ ]	Plants

Methods

n/a	Involved in the study	
[x]	[ ]	ChIP-seq
[ ]	[x]	Flow cytometry
[x]	[ ]	MRI-based neuroimaging

Antibodies

Antibodies used	Ghost Dye V510 (Cat # SKU 13-0870-T100, Cytek Biosciences, dilution 1:1000), PerCP/Cyanine5.5 anti-mouse TCRβ chain Antibody

(Biolegend, Cat#109227, Clone H57-597, dilution 1:200), BUV395 Rat Anti-Mouse CD4 (BD Biosciences, Cat#563790, clone GK1.5, dilution 1:200), BUV496 Rat Anti-Mouse CD8a (BD Biosciences, Cat#569181, Clone 53-6.7, dilution 1:200), Brilliant Ultra Violet™ 563 CD25 monoclonal antibody (Thermofisher, Cat# 365-0251-82, Clone PC61.5, dilution 1:100), BUV615 Rat Anti-Mouse CD24 (BD biosciences, Cat#751499, Clone M1/69, dilution 1:200), BV711 Rat Anti-Mouse CD11a (BD biosciences, Cat#740676, Clone M1/4, dilution 1:200), BV570 anti-mouse CD44 Antibody (Biolegend, Cat#103037, Clone IM7, dilution 1:100), PE/Cy7 anti-mouse CD62L Antibody (Biolegend, Cat#104418, Clone, MEL-14, dilution 1:100), APC/Cy7 anti-mouse PD-1 Antibody (Biolegend, Cat#135224, Clone 29F.1A12, dilution 1:100), BV785 anti-mouse antibody (Biolegend, Cat#104543, Clone H1.2F3, dilution 1:100), BV750 Rat Anti-Mouse CD117 (BD Biosciences, Cat#747412, Clone 2B8, dilution 1:100), BV650 anti-mouse CX3CR1 (Biolegend, Cat#149033, Clone SA011F11, dilution 1:100), BUV737 Rat Anti-Mouse CD127 (BD Biosciences, Cat#612841, Clone SB/199, dilution 1:100), PE/Fire 810 anti-mouse Tim-3 (Biolegend, Cat#149033, Clone RMT3-23, dilution 1:100), BUV805 anti-mouse CD11b (eBioscience, Cat#368-0112-82, Clone M1/70, dilution 1:100), PE/Fire 700 anti-mouse NK1.1(Biolegend, Cat#156528, Clone S17016D, dilution 1:100), cFluor UV440 anti-mouse CD19 (Cytek Biosciences, Cat# SKU R7-20835, Clone 6B5, dilution 1:100), RB780 Hamster Anti-Mouse CD11c (BD Biosciences, Cat #755338, Clone HL3, dilution 1:100), PE anti-mouse CD55 (DAF) antibody (Biolegend, Cat#131804, Clone RIKO-3, dilution 1:200), APC anti-mouse H-2Kb Antibody (Biolegend, Cat#116518, Clone AF6-88.5,, dilution 1:100), anti-mouse NKG7 antibody (described in this paper) , RB545 anti-Tbet (BD biosciences, Cat#569253, Clone O4-46,, dilution 1:50), BUV395 anti-Eomes (BD biosciences, Cat#567171, Clone X4-83, dilution 1:100), PE anti-Granzyme B Recombinant Antibody (Biolegend, Cat#396406, Clone QA18A28, dilution 1:100), BV421 anti-Perforin antibody (Biolegend, Cat#154319, Clone S16009A, dilution 1:100), BUV661 Hamster anti-KLRG1(BD biosciences, Cat#741586, Clone 2F1, dilution 1:100), anti-TOX Antibody- REAfinity (Miltenyl Biotech, Cat#130-118-335, Clone REA473, dilution 1:50), Pacific blue anti-TCF1 antibody (Cell signaling technology, Cat#9066S, Clone C63D9, dilution 1:100), APC anti-mouse CD107a antibody (Biolegend, Cat#505809, Clone XMG1.2, dilution 1:100), polyclonal rabbit anti-human NKG7 antibody (described in methods), BV421 anti-human CD3 Antibody (Biolegend, Cat#317344, Clone OKT3), PE-Cy7 mouse anti-human CD8 (BD Biosciences, Cat#557746, Clone RPA-T8), FITC anti-human CD38 (Biolegend, Cat#303503, Clone HIT2), FITC anti-mouse CD45 antibody (Biolegend, Cat#103118, Clone 30-F11), rat anti-mouse CD8a (Biolegend, Clone 53-6.7, Cat#100702), goat anti-rat IgG mouse absorbed biotinylated antibody (Vector laboratories, Cat#BA-9401.5), Goat anti-Rabbit IgG (H+L) Cross-Adsorbed Secondary Antibody, Alexa Fluor™ 568 (Invitrogen, Cat# A-11011), Goat anti-Mouse IgG (H+L) Cross-Adsorbed Secondary Antibody, Alexa Fluor™ 568 (Invitrogen, Cat# A-11004), anti-human CD8 antibody (RIV11 clone, Thermo Scientific, MA5-48276), Goat anti-Rabbit IgG (H+L) Cross-Adsorbed Secondary Antibody, Alexa Fluor 532 (Thermofisher Scientific, Cat#A-11009) and Goat anti-Mouse IgG (H+L) Cross-Adsorbed Secondary Antibody, Alexa Fluor 647 (Thermofisher Scientific, Cat#A-21235) Hamster anti-CD3 monoclonal antibody (clone 145-2C11, eBioscience, Cat #16-0031-82), goat anti-Armenian Hamster IgG (H+L) Alexa Fluor™ 555 secondary antibody (Thermofisher, Cat# A78964)

Validation	All antibodies employed in this study have been validated by commercial vendors. Specification sheet containing technical usage information and QC data for each antibody clone can be obtained from the manufacturer's website by searching for the associated catalog number. NKG7 antibodies used in this study are validated in-house by CRISPR-Cas9 knockout followed by western blotting.

Eukaryotic cell lines

Policy information about cell lines and Sex and Gender in Research

Cell line source(s)	MC38 colon adenocarcinoma cells were purchased from MIlliporeSigma (SCC172)<br>B16-OVA murine melanoma cell line was a gift from Dr. Richard Vile at Mayo Clinic Rochester.<br>B16-F10 murine melanoma cell line was purchased from ATCC (CRL-6475).<br>P815 mastocytoma cells were obtained from the American Type Culture Collection (ATCC® TIB-64™) and cultured using DMEM complete medium.
Authentication	Authentication was performed using short tandem repeat (STR) profiling for B16 cells (ATCC) and MC38 cells (IDEXX Laboratories). For other murine cell lines, authentication was based on source verification and consistency with expected growth characteristics and morphology.
Mycoplasma contamination	All cell lines are routinely tested for mycoplasma and were mycoplasma-free when used in experiments.
Commonly misidentified lines (See ICLAC register)	None were used.

Animals and other research organisms

Policy information about studies involving animals; ARRIVE guidelines recommended for reporting animal research, and Sex and Gender in Research

Laboratory animals	All experimental procedures were approved by the Institutional Animal Care and Use Committee (IACUC) at Mayo Clinic Rochester. Animals were maintained under specific pathogen-free conditions and housed in filter-top cages with access to food pellets and water under controlled environmental conditions (20–22 °C, 30–70% relative humidity) and a 12 h light/12 h dark cycle. Mice were euthanized by carbon dioxide inhalation in accordance with approved institutional protocols.Pdcd1flox/flox (fl/fl) mice on a C57BL/6 background were a kind gift from Dr. Vassiliki A. Boussiotis from Harvard Medical School, and the protocol for generating these mice has been previously described38. Nkg7fl/fl mice on a C57BL/6 background was generated by inGenious Targeting Laboratory (Ronkonkoma, NY) and details have been described39. E8I CD8-Cre mice were purchased from the Jackson Laboratory (C57BL/6-Tg (Cd8a-cre)1Itan/J, Strain stock #: 008766). The Pdcd1fl/fl E8I CD8Cre (CD8-Pdcd1 cKO) mice were generated by crossing Pdcd1fl/fl mice with E8I CD8-Cre mice. The Pdcd1fl/fl Nkg7fl/fl E8I CD8Cre+ (CD8-Pdcd1-Nkg7 dcKO) mice were generated by crossing Pdcd1fl/fl mice and Nkg7fl/fl mice to generate Pdcd1fl/fl Nkg7fl/fl and cross with E8I CD8-Cre mice.
Wild animals	N/A
Reporting on sex	In all experiments, male and female mice were randomly assigned for experiments.

Field-collected samples	N/A
Ethics oversight	Mayo Clinic IACUC #- A00006353-21-R24. A00002759-17-R23

Note that full information on the approval of the study protocol must also be provided in the manuscript.

Plants

Seed stocks	N/A
Novel plant genotypes	N/A
Authentication	N/A

Flow Cytometry

Plots

Confirm that:

- [x] The axis labels state the marker and fluorochrome used (e.g. CD4-FITC).
- [x] The axis scales are clearly visible. Include numbers along axes only for bottom left plot of group (a 'group' is an analysis of identical markers).
- [x] All plots are contour plots with outliers or pseudocolor plots.
- [x] A numerical value for number of cells or percentage (with statistics) is provided.

Methodology

Sample preparation	Mouse splenic and thymic CD8+ T cells were isolated using CD8a+ T cell isolation kit (Miltenyi Biotec, Cat#130-104-075) according to manufacturer instructions. For isolating CD8+ tumor infiltrating lymphocytes (TILs), tumors were first cut into small pieces of 2-4mm and digested using tumor dissociation kit at 37°C for 45 minutes with gentle agitation (Miltenyi Biotec, Cat#130-096-730) according to manufacturer instructions. Dead cells were further removed using Dead cell Removal kit (Miltenyi Biotec, Cat# 130-090-101). CD8+ TILs were then isolated using CD8 (TIL) microbeads, mouse (Miltenyi Biotec, Cat#130-116-478) based on manufacturer instructions. For in vivo CD45 intravenous labeling, mice were injected intravenously with 2 µg of FITC anti-mouse CD45 antibody (Biolegend, Cat#103118, Clone 30-F11) in 200 µL of PBS via the retro-orbital sinus 3 minutes before euthanasia. Thymus, spleen and peripheral blood were immediately harvested, and single-cell suspensions were prepared.
Instrument	All samples were acquired on a a CytoFLEX LX with CytExpert software (Beckman Coulter), or a Cytek Aurora with SpectroFlo software.
Software	All the analyses were performed using the FlowJo software (V10.0.0).
Cell population abundance	Column-based separation and dead cell removal kit was used to ensure the purity of relevant population >90%.
Gating strategy	All cells were gated based on FSC vs SSC and further only selecting singlets. Dead cells were then removed, followed by gating on appropriate cell types based on specific markers. Gating strategies were set on the isotype controls or FMOs to ensure clear separation between populations. Gates remain constant across all the comparing samples.

- [x] Tick this box to confirm that a figure exemplifying the gating strategy is provided in the Supplementary Information.
