# Supplementary material for: PD-1 regulates latent effector differentiation of thymic cytotoxic CD8+ T cells
Source: Nat Commun. 2026 May 23;17:6769. doi: 10.1038/s41467-026-73392-7 (PMC13385908; doi:10.1038/s41467-026-73392-7)
Supplement: Supplementary file 5 — Transparent Peer Review File [file 41467_2026_73392_MOESM5_ESM.pdf]

nature portfolio

Peer Review File

PD-1 regulates latent effector differentiation of thymic cytotoxic CD8⁺ T cells

Corresponding Author: Dr Haidong Dong

This file contains all reviewer reports in order by version, followed by all author rebuttals in order by version.

Version 0:

Reviewer comments:

Reviewer #1

(Remarks to the Author)
The study by Mao et al. aims to disentangle how the effector potential of CD8 thymocytes is regulated and how this contributes to the anti-tumoral responses. Even though the role of PD-1 in thymic selection had been studied, yet it is not fully understood what PD-1 signaling does at later phases of immune responses. The authors therefore employ a very elegent system with the E8I -driven deletion of PD1 to restrict the deletion to the later phase in the thymus, i.e. after the event of positive selection. With this deletion model, the authors find that PD-1 restricts the cytotoxic and exhaustion potential of thymic SP CD8 T cells, which limits the thymic output of effector T cells to drive peripheral tumor immunity, and propose that PD1 blockade in the thymuc could potentially help generate durable anti-tumor responses.

In my view, the study is interesting and provides insights in the consequences of PD1 deficiency in single positive thymocytes after the event of positive selection. The study seems to be founded on well performed experiments. I have primarily issues with the data interpretation.

1) General comment: the authors focus in their analysis on the functional effects of PD1 deficiency. To my opinion they miss out on another important aspect which could just as well explain their findings: namely the number of CD8 T cells in the peripery. Are they the same or does the PD1 deficiency lead to more T cells/million blood cells/splenocytes? Or is only the ratio of naïve versus effector altered? Provided the role of PD1 in lowering the activation threshold, does PD1 deficiency upon challenge result in much stronger proliferation? And thus more CD8 effector cells overall? Can that explain the data? The authors may already be able to extract those data from their experiments. Importantly it will help interpret the data.
2) Similarly, how does PD1 deficiency influence the TCR landscape? Does it result in expansion of some clones? In fact the data in Figure 4E/F already suggest such effect. This can also affect the T cell responses.

3) Figure 1 (line 82 onwards until 117): Considering PD1 deficient SP T cells as with 'activation/exhaustion' potential:
I fully agree with the activation potential. The findings that PD1 deficiency results in loss of the naïve phenotype is interesting and resonates with the data of Honjo and Ahmed showing that PD1 acts as a rheostat under physiological conditions that increases the threshold for responding to TCR triggering. The conclusion of the authors that T cells in cluster 1 of the RNA seq data (and the subsequent flow cytometric analysis) are more in an activated state is therefore well founded. However, it seems important to me to not only deduct the potential from gene/protein expression but also show whether these cells now respond within 4h by making cytokines, chemokines, acting as good effector cells. As naïve cells require at least 1 day to respond, this will confirm that T cells already in the thymus and possibly also in the periphery (splenic cells) are ahead in their differentiation path towards effector cells/ are real effector cells (comparable to virtual memory T cells.

4) Same point: on the 'exhaustion' potential used by the authors: this conclusion seems far-fetched. The so-called 'exhaustion'markers are all expressed in T cells upon activation. For instance, the authors refer to TOX solely as exhaustion marker. I would like to refer to a recent study from the Prlic lab (https://insight.jci.org/articles/view/150744#B6), clearly demonstrating that TOX also is expressed upon T cell activation. So again to me the data fully align with considering PD1 as rheostat that helps T cells in residing in a naïve state rather than becoming activated by small triggers. That TGF7 which is downstream of TCR engagement is increased also fits in this profile. This example for TOX also holds true for pretty much all other markers used to indicate 'exhausted' T cells in the context of chronic infection/tumors. Therefore, semantically- it is wrong to call this an "exhaustion potential". I don't even know what it means, as theoretically all T cells (including naïve T cells) have the potential to become exhausted provided they are exposed to the appropriate environment. To me these data

only again point to the fact that PD1 is required for SP cells to keep the cells in a naïve state and to keep the threshold of activation high enough (again shown by Honjo/Ahmed for peripheral naïve T cells).

5) It would be interesting to disentangle whether TCR triggering is required for this loss of naiveity and whether antigen specific activation is the trigger, or rather non-specific signals. Thus which signals PD-1 blocks in order to maintain the naïve state of SP thymocytes. With that note, I noticed that the CD3/CD8 expression at least on Gene level (Fig 1D) appears to be increased in PD1 deficient SP thymocytes. How do the authors interpret these findings?

6) The human data on Figure 4 are interesting but again to my opinion over interpreted. Stating that they 'demonstrate that PD1 regulates thymic output of Teff cells that are tumor reactive in peripheral tissues' is clearly an overstatement. There is no more than a small correlation, as this is not well studied. The authors should either provide data that better support such bold statements or tone down. On the same note, I find Extended data 7 belonging to this figure very difficult to understand and interpret.
7) Figure 5 shows tumor take, not tumor clearance. For tumor clearance T cell transfer experiments are required. Overall, I again find the data difficult to interpret. Is it a numbers game of more effector T cells? As the cells probably will produce much more rapidly and more quantities of IFNg and other cytokines upon activation: how to they shape the other immune cells which will respond to this altered response? There is no evidence that the thymocytes in fact contribute to better killing etc (as already indicated for the model description in figure 1). Such data are also lacking here. I understand that the tumors do not take, rendering it more difficult to follow immune responses. However, they could at least follow follow (the tumor specific) T cell responses in the blood which can serve as a proxy of what is going on.
8) The vaccination model already is better in studying the tumor responses. Yet again, it only studies tumor take and not the effect on tumor growth etc. It shows that T cells have the capacity to become memory cells- what type of memory cells? why are again T cell responses not followed? The authors lean in the manuscript too much on assumptions and deductions from their findings rather than confirming them experimentally.

9) Figure 3: the authors isolated and measured the TILs from the mice: again: what are the numbers of Tcells that infiltrate tumors (per gramm Tissue) from WT versus PD1 ko cells? what is their functional profile?

10) Also their main conclusion that the thymocytes contribute to anti-tumoral responses and that aPD1 blocking can contribute to it seems an interesting hypothesis but at this stage not well founded by experiments

Reviewer #2

(Remarks to the Author)
This manuscript "PD-1 limits thymic output of effector CD8+ T cells for peripheral tumor immunity" by Zhiming Mao and Colleagues uncovers a previously unappreciated population of total thymocytes in conditional PD-1 knockout mice. Using a combination of single-cell RNA sequencing, reporter genetics, and flow cytometry, the authors show that in the absence of PD 1, these cells acquire a full cytotoxic transcriptomic profile, challenging the classical view that the thymus lacks effector like $CD8^+$ differentiation. The authors present their findings in the context of checkpoint inhibition, showing that PD 1 deficient SP $CD8^+$ T cells infiltrate tumors more effectively, mediate early rejection or growth delay, and show enhanced effector molecule expression (e.g., Granzyme B, NKG7), but also increased expression of exhaustion markers (TOX, Havcr2) and ultimately lose cytotoxic function by day 21. These cells share TCR clonotypes with thymic cells, suggesting a thymic origin.

In my view, this study should not primarily be framed as a checkpoint-focused investigation. Its novelty lies in uncovering an aspect of thymic T cell development—specifically, the emergence of cytotoxic-like $CD8^+$ thymocytes under the control of PD-1. I strongly recommend reframing the manuscript to emphasize thymic T cell maturation and selection. This would more effectively highlight the mechanistic novelty of the findings and better align the work with the interests of a more relevant immunology-focused audience. Even with a revised focus, several major concerns remain.

Major Concerns
Extended Data Fig 1 F. Authors show that PD-1 knockout was achieved. The percentage of PD-1–positive cells in knockout mice remains at approximately 7%. How reliable is the knockout under these conditions? A general defect in T cells of cKO has to be ruled out. It would help to see as control staining of Eomes, Perforin… side-by-side using naive CD8 T cells from the periphery of the same mouse (plus FMO controls showing the FACS plots). Was the absence of PD-1 confirmed in the sequencing data?

Is PD-1 just lower expressed (deficient) or is it a complete knockout? Is the knockout stable over time, or can PD-1 expression re-emerge in these cells? What level of off-target effects can be expected from the knockout strain used? Given that PD-1 regulates a variety of pathways, it remains unclear whether the observed effects are specifically due to PD-1 deficiency or reflect broader dysregulation of immune signaling. According to the signaling pathway analysis, immune-related pathways appear strongly affected, while pathways involved in protein synthesis and processing are downregulated. This raises the question to what extent the phenotypes can be attributed directly to PD-1 loss, or whether they result from secondary effects.

Fig.1 E-J. and Text. Flow cytometry analysis of thymic SP CD8 T cells after isolation show increased expression of cytotoxic and exhaustion markers in PD cKO mice. Comment: What are the possible reasons for the presence of cytotoxic cells in the thymus? What mechanisms or factors account for the fact that these cells already exhibit activation and enhanced cytotoxic potential as well as exhaustion like features?

Extended Data Fig. 2D: Core TCR signalling and protein synthesis pathways are down regulated in PD 1 / SP CD8 T cells—how can these cells still mount effective antigen processing and cytotoxic function under such impairment? How reliable are the conclusions about their cytotoxic capacity when central pathways required for T cell activation and function appear inhibited? A ex vivo killing assay would further strengthen the central claim that these thymic cells are bona fide functional effectors.

Fig. 2 N–O. The sequencing tracks show higher Perforin, Granzyme B, Havcr2 and Tcf7 expression in PD 1 / cells, but it is unclear whether all displayed differences were formally tested. Please provide the statistical workflow—test applied, exact p values and FDR adjusted q values—for each gene. Without these metrics, several of the visual shifts could fall within noise and may not be biologically meaningful.

Extended Data Fig 3. To exclude that increased cytotoxic levels in thymic SP CD8 T cells are not recirculated mature CD8 cells, CD45 labeling was performed. This is nicely shown. This essential data belongs to the main figure part.

Fig 3 PD-1 knockout CD8 T cells exhibit enhanced effector function in vivo, as demonstrated by improved tumor control and greater tumor cell elimination. However, this effect appears restricted to tumors with high or intermediate immunogenicity, indicating that sufficient antigenic stimulation is required to unleash the full potential of these cells. By day 12, PD-1-deficient CD8 T cells show increased expression of exhaustion markers alongside an effector memory phenotype. This coincides with sustained upregulation of cytotoxic molecules such as Granzyme B and Perforin, suggesting that heightened effector activity is accompanied by progressive functional exhaustion.
Comment: Following immunization, are more recent thymic emigrants (RTEs) retained in the thymus to prime SP CD8 T cells with the specific peptide? Additionally, what is the role and phenotype of splenic CD8 T cells during this phase? Did the authors investigate whether the thymus still harbors mature, naïve T cells that could be mobilized and activated? Alternatively, could the early strong response result in a lymphopenic state, leaving insufficient numbers of functional T cells for sustained tumor control? This interpretation is supported by the observation in lines 143–144 that “mature peripheral $CD8^+$ T cells were reduced in frequency”. What are the implications of this decline for the long-term efficacy of PD-1-deficient CD8 T cells in anti-tumor immunity? What underlies this loss of function within the T cell pool by day 21?

In Fig. 5 E–F the PD 1 knock out mice display a brisk effector $CD8^+$ expansion that collapses after day 21, but three points remain unclear: first, why was an ex vivo restimulation step required instead of analysing the cells directly; second, would immunisation with a strongly immunogenic peptide elicit a similarly rapid burst and tumour rejection, thereby distinguishing PD 1 specific effects from generic antigen driven proliferation; and third, what is the phenotype or function of the residual cells at day 21—do they re express exhaustion markers or lose cytolytic capacity? Clarifying these issues would greatly refine the interpretation of the T cell dynamics.

Fig 5 H-J When exactly does the PD-L1 blockade occur in the experimental timeline? Do the targeted T cells exhibit features of terminal exhaustion, or do they retain the potential for functional rescue? It is well-established that PD-1/PD-L1 blockade primarily restores cells at a pre-exhausted or stem-like stage, but not those that are terminally exhausted. According to the Materials section (line 355), PD-L1 antibody was injected 7 days post tumor implantation. How does the tumor growth delay compare between fl/fl and fl/fl plus PD-L1 blockade, as well as KO and KO plus PD-L1 blockade groups? Is the observed tumor growth delay truly attributable to PD-L1 inhibition, or could the antibody alter immunological synapse conformation, thereby enhancing CD28/TCR signaling?

Finally, outline whether peripheral blockade of PD-1 or PD-L1 using specfic antibodies can recapitulate the reported thymic specific PD-1 cKO phenotype.

The discussion must be embedded in a larger context; it appears to be mainly descriptive of the results.

Minor Concern
Fig. 4 could be moved to an Extended Data Fig.

Reporting numbers of samples (n) is necessary throughout to give the reader an idea of the statistics.

Show the p-values for the correction of multiple comparisons as supplementary information. This is not clearly visible.

Furthermore, is a sequencing depth of approximately 40,000 reads per cell sufficient to generate reliable and statistically robust results? Could low level transcription inflate cluster assignment.

Reviewer #3

(Remarks to the Author)
Majors:

1 The scRNA-seq data (Figure 1B and 1C) indicate that all highlighted genes, except Nkg7, are enriched in CD8-Pdcd1 cKO SP CD8^{+} T cells versus control SP CD8^{+} T cells, while Cluster 1 is markedly less represented than Cluster 0. The authors' emphasis on cytotoxicity and exhaustion, with little consideration of stem-like characteristics, seems incomplete. A similar oversight is evident in their analysis of splenic SP CD8^{+} T cells.
2 The authors state that isolated thymic SP CD8^{+} T cells were stimulated with PMA and ionomycin. However, it is unclear why the control Pdcd1fl/fl SP CD8^{+} T cells fail to exhibit activation markers such as CD107a, NKG7, and GZMB (Figure 1N–R). The validity of the stimulation conditions or the responsiveness of the control cells are needed.
3 The authors propose that NKG7^{+} Pdcd1 cKO CD8^{+} T cells are the main contributors to tumor control. Have the authors considered performing functional validation, such as selective depletion or adoptive transfer of these NKG7^{+} subsets, to confirm their direct anti-tumor activity?
4 The author claims that PD-L1 blockade releases CD80 from its interaction with PD-L1, thereby permitting CD80 to engage CD28, which in turn promotes effector T cell expansion via co-stimulatory signaling and mitigates exhaustion. To substantiate this mechanistic claim, direct experimental evidence should be provided.

Minors:
1 Based on the UMAP in Extended Data Fig. 7B, human NKG7^{+}CD8^{+} and NKG7^{-}CD8^{+} T cells did not exhibit significant transcriptional differences, either within individual patients or between different patients.
2 It may be interesting to integrate scRNA-seq data of CD8^{+} T cells from the thymus, spleen, and tumor-infiltrating lymphocytes (TILs).
3 The authors suggest that PD-1 KO CD8^{+} T cells can exhibit the dual role of cytotoxic and exhaustion functions against tumors, depending on the tumor context. However, why do Pdcd1 cKO CD8^{+} T cells effectively control tumors only when tumor immunogenicity is high or intermediate? Does the underlying mechanism among the three tumor types appear to be the same? As the authors mention, immunized CD8-Pdcd1 cKO cells can develop long-term memory rather than exhaustion phenotypes. Why, then, does high immunogenicity drive the transition of effector CD8^{+} T cells toward a memory phenotype rather than exhaustion? These need to be at least discussed.
4. Both mouse data and HCC patient data indicate that Pdcd1 cKO CD8^{+} T cells expressing NKG7 contribute to anti-tumor functions. Can NKG7- CD8+ TILs be used as ICB treatment biomarker for tumor patients? Discussion on this point is suggested.

Reviewer #4

(Remarks to the Author)
Majors:
1 The scRNA-seq data (Figure 1B and 1C) indicate that all highlighted genes, except Nkg7, are enriched in CD8-Pdcd1 cKO SP CD8^{+} T cells versus control SP CD8^{+} T cells, while Cluster 1 is markedly less represented than Cluster 0. The authors' emphasis on cytotoxicity and exhaustion, with little consideration of stem-like characteristics, seems incomplete. A similar oversight is evident in their analysis of splenic SP CD8^{+} T cells.
2 The authors state that isolated thymic SP CD8^{+} T cells were stimulated with PMA and ionomycin. However, it is unclear why the control Pdcd1fl/fl SP CD8^{+} T cells fail to exhibit activation markers such as CD107a, NKG7, and GZMB (Figure 1N–R). This raises questions about the validity of the stimulation conditions or the responsiveness of the control cells.
3 The authors suggest that PD-1 KO CD8^{+} T cells can exhibit the dual role of cytotoxic and exhaustion functions against tumors, depending on the tumor context. However, why do Pdcd1 cKO CD8^{+} T cells effectively control tumors only when tumor immunogenicity is high or intermediate? Does the underlying mechanism among the three tumor types appear to be the same? As the authors mention, immunized CD8-Pdcd1 cKO cells can develop long-term memory rather than exhaustion phenotypes. Why, then, does high immunogenicity drive the transition of effector CD8^{+} T cells toward a memory phenotype rather than exhaustion?
4 The authors propose that NKG7^{+} Pdcd1 cKO CD8^{+} T cells are the main contributors to tumor control. Have the authors considered performing functional validation, such as selective depletion or adoptive transfer of these NKG7^{+} subsets, to confirm their direct anti-tumor activity?
5. Both mouse data and HCC patient data indicate that Pdcd1 cKO CD8^{+} T cells expressing NKG7 contribute to anti-tumor functions. Why is it that NKG7^{-} CD8^{+} TILs are enriched in the PD-1/PD-L1 pathway, whereas NKG7^{+} CD8^{+} TILs are not enriched in the "PD-1/PD-L1 cancer immunotherapy" pathway? Can NKG7- CD8+ TILs be used as ICB treatment biomarker for tumor patients?
6 The author claims that PD-L1 blockade releases CD80 from its interaction with PD-L1, thereby permitting CD80 to engage CD28, which in turn promotes effector T cell expansion via co-stimulatory signaling and mitigates exhaustion. To substantiate this mechanistic claim, direct experimental evidence should be provided.

Minors:
1 Based on the UMAP in Extended Data Fig. 7B, human NKG7^{+}CD8^{+} and NKG7^{-}CD8^{+} T cells did not exhibit significant transcriptional differences, either within individual patients or between different patients.
2 It may be interesting to integrate scRNA-seq data of CD8^{+} T cells from the thymus, spleen, and tumor-infiltrating lymphocytes (TILs).

Version 1:

Reviewer comments:

Reviewer #1

(Remarks to the Author)
I appreciate the in-depth response to my points.

Reviewer #2

(Remarks to the Author)
Summary
In this revised submission of the manuscript by Mao et al., the authors have clearly made a substantial effort to address prior reviewer concerns, including adding an ex vivo thymocyte-mediated killing assay, expanding the thymus–tumor TCR sequencing data set with additional mice, and incorporating RAG-GFP reporter experiments to track recent thymic emigrants (RTEs) in tumors with and without anti–PD-1 therapy. They also reframe the manuscript more explicitly around thymic programming ("latent effector differentiation") and reduce overstatement in places. The manuscript proposes a thymus-intrinsic checkpoint function for PD-1 in shaping the CD8 single-positive compartment, suggesting that loss of PD-1 permits emergence of an effector-like/cytotoxic program already in the thymus and that these cells contribute to tumor control. The most compelling novelty remains the thymic angle (resident CD8 SP with effector features) together with the observation that genetic Pdcd1 deletion is not trivially phenocopied by therapeutic anti–PD-1 blockade. However, the main weaknesses remain that the functional cytotoxicity claim is still not supported by statistically robust killing data, and that the thymus–TIL TCR overlap is still presented largely descriptively without a rigorous null model controlling for sampling depth and clonality. The peripheral phenotyping is extensive but in many places confirmatory of well-described PD-1–loss phenotypes and could be substantially condensed.
Major points
1. Scope and focus of peripheral assays. The thymus-centered concept is strong: the manuscript links PD-1 loss to an effector-like CD8 SP program in the thymus, makes a reasonable effort to address recirculation, and connects the program to tumor control with functional genetics and a thymus–tumor bridge. In contrast, much of the peripheral spleen/blood baseline phenotyping (multiple redundant effector/exhaustion marker panels in peripheral CD44+CD62L– cells, repeated activation/proxy readouts, and additional spleen scRNA feature/dot plots) remains largely confirmatory of peripheral PD-1–loss effector skewing. For a Nature Communications-level research manuscript, I recommend condensing these readouts and moving most redundant peripheral marker surveys to the Supplement unless they directly a) exclude alternative explanations, b) connect thymus output to tumors, or c) test causality.
2. Newly added thymocyte-mediated killing assay does not robustly support active cytotoxicity
I appreciate that the authors added an ex vivo thymocyte-mediated cytotoxicity assay in response to earlier concerns. However, as presented, the killing assay is not statistically convincing: only one E:T condition shows a borderline effect ($p = 0.0503$), while the other dilutions are clearly non-significant, arguing against a consistent dose–response. Because multiple E:T ratios are tested in parallel, these comparisons form a multiple-testing family and the borderline result would not survive simple correction; at most, this should be labeled exploratory/trend rather than evidence of bona fide/active cytotoxicity. A more appropriate analysis would treat the dilution series globally (two-way ANOVA or mixed-effects model with genotype × E:T interaction), or use a curve-based summary metric (AUC/EC50), and only proceed to corrected post hoc comparisons if the global effect is significant.
3. Expanded thymus–TIL TCR overlap still requires depth- and clonality-aware statistics
I appreciate that the authors expanded the TCR sequencing to additional mice/tumor settings and added a revised wording ("latent effector differentiation … can contribute to the TCR repertoire of TILs"). Nevertheless, the thymus–TIL overlap remains a central dataset supporting the proposed thymus-to-tumor link, and it is still not evaluated against an appropriate expectation. Even when all samples are processed identically and the same top-N strategy is used, observed overlap can be strongly influenced by effective sequencing depth, numbers of unique clonotypes, and clonotype frequency distributions (oligo- vs polyclonality), which is particularly consequential with few mice.
At minimum, the authors should:
• rarefy/downsample thymus and TIL repertoires to matched depth within each mouse
• report overlap using depth-robust metrics (e.g., Jaccard plus a frequency-weighted metric such as Morisita–Horn)
• benchmark observed overlap against a within-mouse permutation/bootstrapping null model that preserves marginal clonotype frequencies
• present mouse-level statistics and, if possible, demonstrate consistency across mice
This would clarify whether overlap reflects enrichment beyond sampling/detection and overall clonality.
4. Genetic deletion versus therapeutic blockade: distinguish programming from acute absence of PD-1 signaling
I appreciate that the revised manuscript includes additional data comparing peripheral anti–PD-1 blockade to the genetic setting (including RAG-GFP/RTE analyses and peripheral blockade experiments). The observation that therapeutic anti–PD-1 does not phenocopy genetic Pdcd1 deletion remains notable and consistent with an early programming/imprinting effect, but it does not by itself establish developmental imprinting. Because Pdcd1 loss removes an inhibitory brake at the time of stimulation, several functional readouts could be explained by acute absence of PD-1 signaling rather than stable developmental reprogramming. With minimal additional work, the authors should show a direct side-by-side in vitro comparison under identical conditions in which WT cells are assessed with acute PD-1/PD-L1 blockade (matched stimulation and E:T) directly against Pdcd1 cKO cells, ideally using rigorously naïve CD8 T cells from young, tumor-free, age-matched mice to avoid confounding by prior activation/homeostatic expansion. Demonstrating that the cKO phenotype exceeds what acute blockade produces would substantially strengthen the imprinting interpretation without requiring

additional tumor models. If blockade effects are central to conclusions, pharmacodynamic target engagement (at least one clear regimen/engagement control) should be documented.
5. Statistical framework and unit of replication (important for confidence across the manuscript)
Across several panels (including “selected genes” dot plots and multiple parallel flow endpoints), it is not always clear what the unit of replication is (mice/samples vs cells), raising concern about pseudoreplication. Multiple-testing control is also not consistently described for families of related endpoints. Please clarify the unit of replication for each analysis, report mouse-level statistics, and either predefine primary endpoints or apply appropriate multiple-comparison correction within each figure/family.
Minor points / clarifications
• For dot plots/selected-gene panels reporting t-test p-values: please specify the replicate unit, whether tests are performed at the mouse/sample level, and why FDR across the tested gene set (or broader universe) is not applied.
• For functional readouts relying on PMA/ionomycin: consider clarifying that these measure maximal effector capacity and may not be optimal to distinguish PD-1 pathway effects that act via TCR/CD28-proximal signaling; TCR-dependent stimulation controls are more informative for the key mechanistic question.
• Where conclusions hinge on resident thymic populations, please provide quantitative summaries across mice of the extent of recirculant exclusion (not necessarily new experiments, but clearer reporting of the existing controls and their variability).

Overall recommendation
The concept is strong and well aligned with the journal’s scope. However, the two central mechanistic claims—functional cytotoxicity of thymic cells and the thymus–tumor clonotype linkage—still require more rigorous quantification and depth-aware statistical treatment to be fully convincing. The requested additions are targeted and should be achievable without expanding into many additional tumor models. Given that these points underpin the manuscript’s main novelty, I do not recommend publication in its current form without addressing them.

Reviewer #3

(Remarks to the Author)
The revised version addresses all the comments. Suggested for publication.

Reviewer #4

(Remarks to the Author)
I co-reviewed this manuscript with one of the reviewers who provided the listed reports. This is part of the Nature Communications initiative to facilitate training in peer review and to provide appropriate recognition for Early Career Researchers who co-review manuscripts.

Version 2:

Reviewer comments:

Reviewer #2

(Remarks to the Author)
The authors have addressed all of my concerns comprehensively. The data are convincing, the interpretation is well supported, and I support publication in Nature Communications.

Open Access This Peer Review File is licensed under a Creative Commons Attribution 4.0 International License, which permits use, sharing, adaptation, distribution and reproduction in any medium or format, as long as you give appropriate credit to the original author(s) and the source, provide a link to the Creative Commons license, and indicate if changes were made.
In cases where reviewers are anonymous, credit should be given to 'Anonymous Referee' and the source.
The images or other third party material in this Peer Review File are included in the article's Creative Commons license, unless indicated otherwise in a credit line to the material. If material is not included in the article's Creative Commons license and your intended use is not permitted by statutory regulation or exceeds the permitted use, you will need to obtain permission directly from the copyright holder.
To view a copy of this license, visit https://creativecommons.org/licenses/by/4.0/

Reviewer's Comments:

Reviewer #1 (Remarks to the Author)

The study by Mao et al. aims to disentangle how the effector potential of CD8 thymocytes is regulated and how this contributes to the anti-tumoral responses. Even though the role of PD-1 in thymic selection had been studied, yet it is not fully understood what PD-1 signaling does at later phases of immune responses. The authors therefore employ a very elegent system with the E8I -driven deletion of PD1 to restrict the deletion to the later phase in the thymus, i.e. after the event of positive selection. With this deletion model, the authors find that PD-1 restricts the cytotoxic and exhaustion potential of thymic SP CD8 T cells, which limits the thymic output of effector T cells to drive peripheral tumor immunity, and propose that PD1 blockade in the thymuc could potentially help generate durable anti-tumor responses.

In my view, the study is interesting and provides insights in the consequences of PD1 deficiency in single positive thymocytes after the event of positive selection. The study seems to be founded on well performed experiments. I have primarily issues with the data interpretation.

We appreciate this reviewer's encouraging comments on the significance and innovation of our study.

1) General comment: the authors focus in their analysis on the functional effects of PD1 deficiency. To my opinion they miss out on another important aspect which could just as well explain their findings: namely the number of CD8 T cells in the peripery. Are they the same or does the PD1 deficiency lead to more T cells/million blood cells/splenocytes? Or is only the ratio of naïve versus effector altered? Provided the role of PD1 in lowering the activation threshold, does PD1 deficiency upon challenge result in much stronger proliferation? And thus more CD8 effector cells overall? Can that explain the data? The authors may already be able to extract those data from their experiments. Importantly it will help interpret the data.

It is a great question! Our three independent experiments show that the percentage of $CD8^+$ T cells in the periphery was comparable between Pdcd1 cKO mice and control mice (**New Fig. 3A**). However, the total number of $CD8^+$ T cells was significantly increased in Pdcd1 cKO mice (**New Fig. 3B**), accompanied by higher proliferative capacity as indicated by the Ki67 staining in splenic $CD8^+$ T cells (**New Fig. 3C**). These data suggest that PD-1 deficiency enhances the proliferative capacity and effector differentiation of $CD8^+$ T cells by lowering the activation threshold.

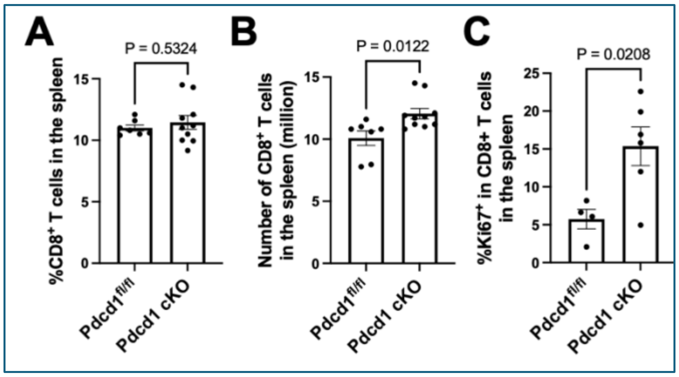

Figure 3. Increased cytotoxic potential of peripheral effector $CD8^+$ cells in the absence of PD-1.

(A-C) Frequencies, absolute numbers, and proliferative capacity of splenic $CD8^+$ T cells in $Pdcd1^{fl/fl}$ (n=7) and Pdcd1 cKO (n=10) mice.

2) Similarly, how does PD1 deficiency influence the TCR landscape? Does it result in expansion of some clones? In fact the data in Figure 4E/F already suggest such effect. This can also affect the T cell responses.

Our new TCR landscape analysis showed that the V(D)J rearrangements were not affected in the thymus or spleen of either control mice or PD-1 cKO mice. Similarly, the V(D)J rearrangement pattern in the thymus and tumor was comparable between these two groups after the tumor challenge. The landscape analysis is also consistent in our new TCR sequencing data of $CD8^+$ T cells in both thymus and B16-F10 tumor (**New Extended Data Fig. 8**). As expected, the E8i enhancer-driven cre deletes Pdcd1 after thymic positive selection, which occurs only after completion of V(D)J combination. In tumor-bearing mice, we found certain TCR clones underwent expansion and the diversity of T cell repertoire decreased in PD-1 cKO mice compared to control mice.

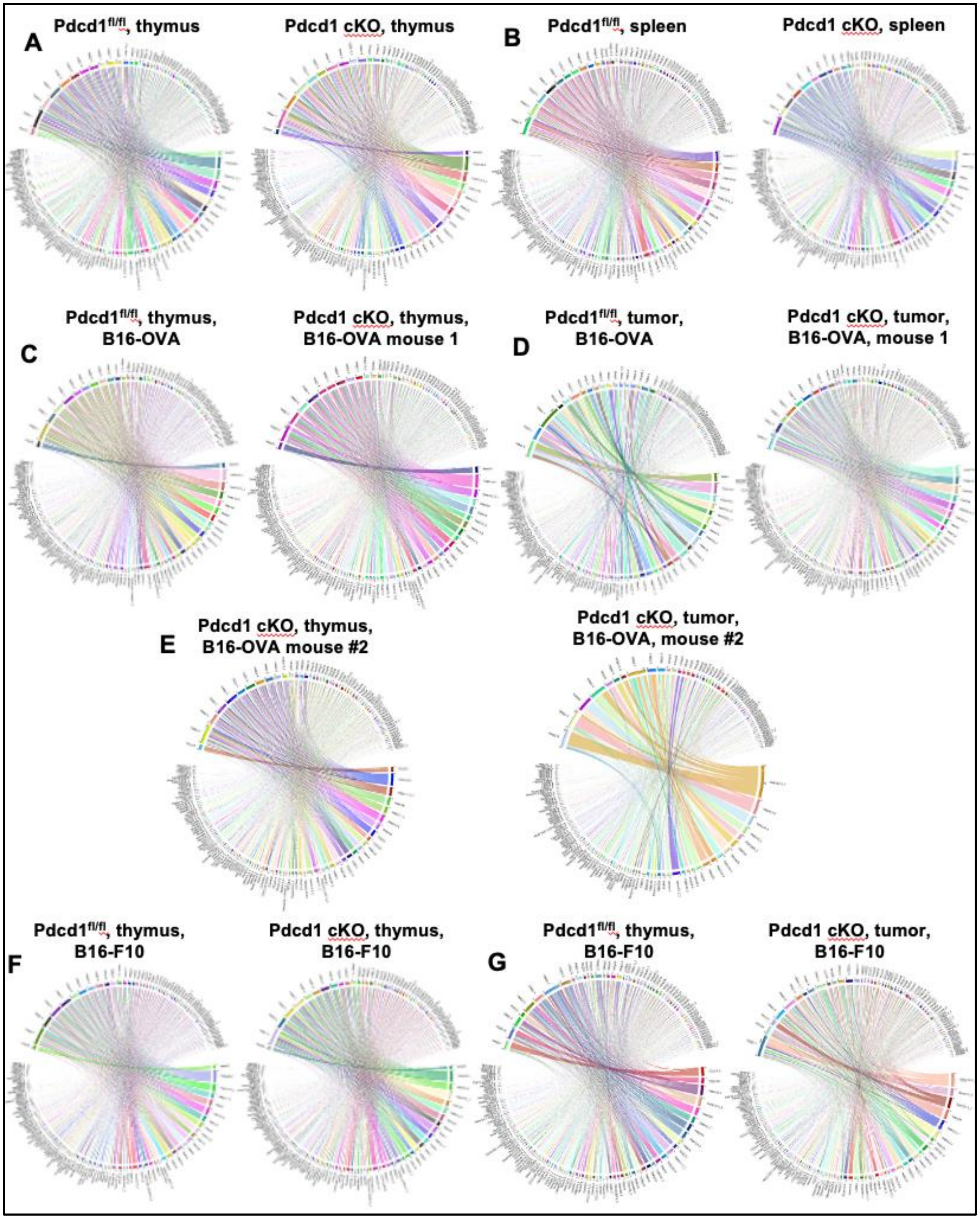

Extended Data Fig 8. TCR landscape analysis in thymus, spleen and tumors. V(D)J rearrangements of

TCR repertoire in the thymus (**A**) and spleen (**B**) of Pdcd1$^{fl/fl}$ and Pdcd1 cKO mice at baseline. V(D)J rearrangements in the thymus (**C**) and tumor (**D**) of Pdcd1$^{fl/fl}$ and Pdcd1 cKO mice at day 12 post B16-OVA tumor injection. (**E**) V(D)J rearrangements in the thymus and tumor of a second CD8-Pdcd1 cKO mouse following B16-OVA tumor injection. (**F, G**) V(D)J rearrangements in the thymus and tumor at day 12 after B16-F10 tumor injection in Pdcd1$^{fl/fl}$ (**F**) and Pdcd1 cKO mice (**G**).

3) Figure 1 (line 82 onwards until 117): Considering PD1 deficient SP T cells as with 'activation/exhaustion' potential:
I fully agree with the activation potential. The findings that PD1 deficiency results in loss of the naïve phenotype is interesting and resonates with the data of Honjo and Ahmed showing that PD1 acts as a rheostat under physiological conditions that increases the threshold for responding to TCR triggering. The conclusion of the authors that T cells in cluster 1 of the RNA seq data (and the subsequent flow cytometric analysis) are more in an activated state is therefore well founded. However, it seems important to me to not only deduct the potential from gene/protein expression but also show whether these cells now respond within 4h by making cytokines, chemokines, acting as good effector cells. As naïve cells require at least 1 day to respond, this will confirm that T cells already in the thymus and possibly also in the periphery (splenic cells) are ahead in their differentiation path towards effector cells/ are real effector cells (comparable to virtual memory T cells.

It is an insightful comment. Accordingly, we assessed degranulation and intracellular cytokine production following 4-hour PMA and ionomycin stimulation of isolated thymic CD8$^+$ T cells and splenic CD8$^+$ T cells. In the thymus, we observed a significant increase in the frequency of CD107a+ degranulating cells in both thymic semi-mature and mature SP CD8+ T cells in the absence of PD-1 (**New Fig. 2F**). From there, the frequency of CD107a$^+$IFNγ$^+$ thymic CD8$^+$ T cells significantly increased in the absence of PD-1 (**New Fig. 2H**), while the frequency of IFNγ$^+$ thymic CD8$^+$ T cells only marginally increased (**New Fig. 2G**). However, the frequencies of TNFα$^+$ thymic CD8+ T cells decreased in both semi-mature and mature thymic SP CD8$^+$ T cells (**New Fig. 2I**), while the percentage of IL-2$^+$ CD8$^+$ T cells did not change in absence of PD-1 (**New Extended Data Fig. 1O**). In the spleen, we found a notable increase in the frequencies of CD107a$^+$ or IFNγ$^+$ CD8$^+$ T cells, but a decrease in TNFα$^+$ CD8$^+$ T cells in the spleen of Pdcd1 cKO mice compared to control mice (**New Fig. 3D-F**). Collectively, our results suggest that latent effector differentiation can generate functional cytotoxic effector CD8$^+$ T cells, but their activity becomes detectable only in the absence of PD-1.

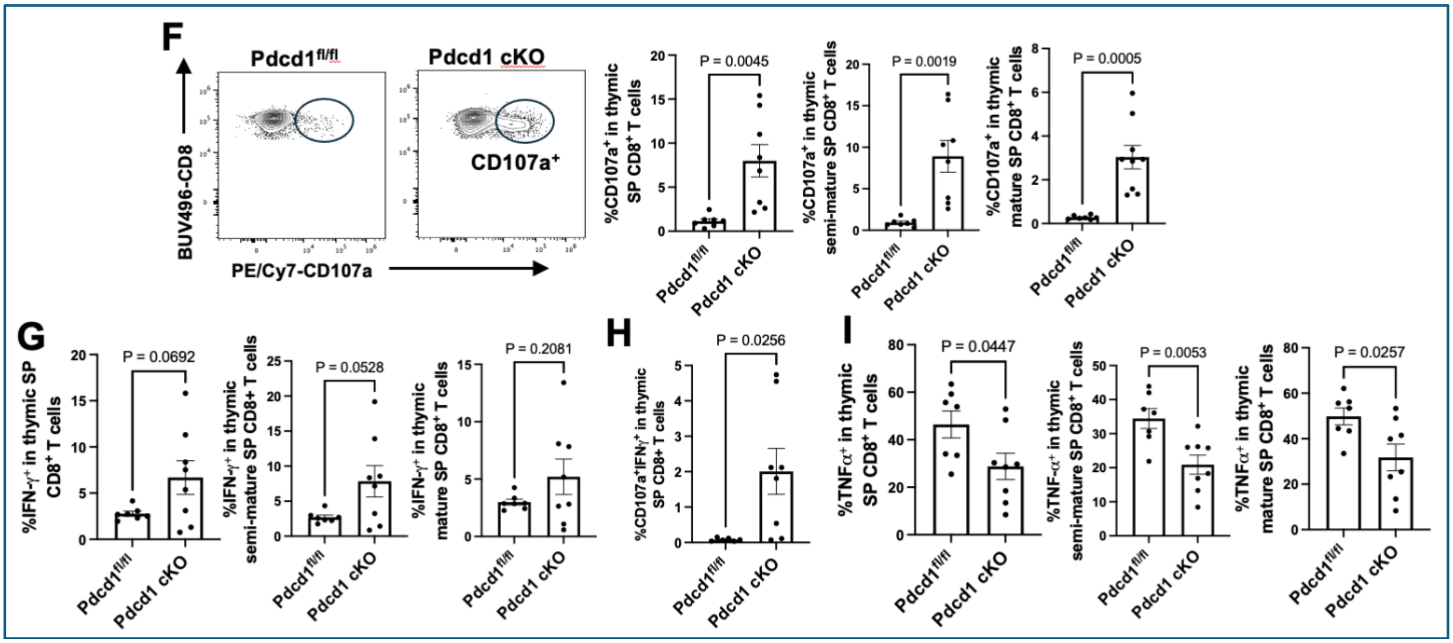

Figure 2. Thymic SP CD8$^+$ T cells exhibited cytotoxic effector function in the absence of PD-1. (F-I) Degranulation and cytokine responses of thymic CD8$^+$ T cells following PMA and ionomycin stimulation for 4 hours in Pdcd1$^{fl/fl}$ (n=7) and Pdcd1 cKO (n=8) mice. (F) Gating strategy (left) and percentage of CD107a$^+$ degranulation in thymic SP, semi-mature SP and mature SP CD8$^+$ T cells (right). (G) Percentage of IFN-γ$^+$ in thymic SP, semi-mature SP and mature SP CD8$^+$ T cells. (H) Percentage of CD107a$^+$ IFN-γ$^+$ thymic SP CD8$^+$ T cells. (I) Percentage of TNF-α$^+$ in thymic SP, semi-mature SP and mature SP CD8$^+$ T cells.

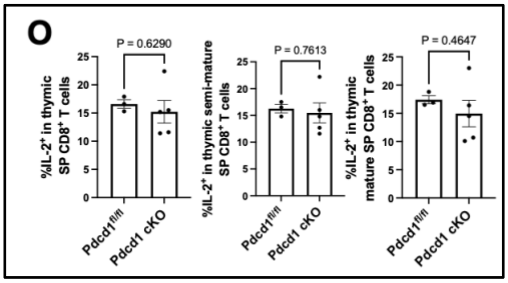

Extended Data Fig 1. Flow cytometry analysis of the thymic profile of single positive CD8$^+$ T cells.

(O) Frequency of IL-2$^+$ in thymic total, semi-mature and mature SP CD8$^+$ T cells following PMA and ionomycin stimulation in Pdcd1$^{fl/fl}$ (n=3) and Pdcd1 cKO (n=5) mice.

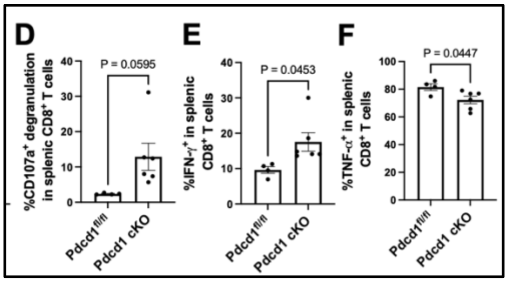

Figure 3. Increased cytotoxic potential of peripheral effector CD8$^+$ cells in the absence of PD-1.
(D-F) Degranulation and cytokine production of splenic CD8$^+$ T cells following PMA and ionomycin stimulation for 4 hours in Pdcd1$^{fl/fl}$ (n=4) and Pdcd1 cKO (n=6) mice. Percentage of CD107a$^+$ (D), IFN-γ$^+$ (E), and TNF-α$^+$(F) splenic CD8$^+$ T cells.

4) Same point: on the 'exhaustion' potential used by the authors: this conclusion seems far-fetched. The so-called 'exhaustion'markers are all expressed in T cells upon activation. For instance, the authors refer to TOX solely as exhaustion marker. I would like to refer to a recent study from the Prlic lab (https://insight.jci.org/articles/view/150744#B6), clearly demonstrating that TOX also is expressed upon T cell activation. So again to me the data fully align with considering PD1 as rheostat that helps T cells in residing in a naïve state rather than becoming activated by small triggers. That TGF7 which is downstream of TCR engagement is increased also fits in this profile. This example for TOX also holds true for pretty much all other markers used to indicate 'exhausted' T cells in the context of chronic infection/tumors. Therefore, semantically- it is wrong to call this an "exhaustion potential". I don't even know what it means, as theoretically all T cells (including naïve T cells) have the potential to become exhausted provided they are exposed to the appropriate environment. To me these data only again point to the fact that PD1 is required for SP cells to keep the cells in a naïve state and to keep the threshold of activation high enough (again shown by Honjo/Ahmed for peripheral naïve T cells).

We greatly appreciate this conceptually insightful comment! Accordingly, we removed the use of "exhaustion potential" from our manuscript as pointed out by this reviewer, those "exhaustion markers" are reflecting an activation status of T cells rather than in "exhaustion state" yet at least in thymus and spleen. We also cited the reference this reviewer provided for us.

5) It would be interesting to disentangle whether TCR triggering is required for this loss of naiveity and whether antigen specific activation is the trigger, or rather non-specific signals. Thus which signals PD-1 blocks in order to maintain the naïve state of SP thymocytes. With that note, I noticed that the CD3/CD8 expression at least on Gene level (Fig 1D) appears to be increased in PD1 deficient SP thymocytes. How do the authors interpret these findings?

That is a very interesting and insightful question. Single-positive (SP) CD8$^+$ T cells have undergone both positive and negative thymic selection, during which their TCRs engage with appropriate ligands. We speculate that basal signaling resulting from prior TCR engagement may initiate effector differentiation, but this process

is restrained by PD-1. PD-1, also induced by earlier TCR signaling, likely functions as a rheostat to modulate this activation. To test whether the disruption of homeostasis/loss of naivety of $CD8^+$ T cells in the absence of PD-1 is due to TCR signaling, we measured Nur77 expression in thymic SP $CD8^+$ T cells. We found that Nur77 expression tended to increase in the absence of PD-1, particularly in semi-mature T cells (**New Extended Data Fig. 1L**). Consequently, the absence of PD-1 may lead to high-strength TCR signals in SP T cells along with their increased expression of CD3 and CD8. This observation suggests a potential regulatory role for PD-1 in regulation of signal strength and expression of TCR, which warrants further investigation.

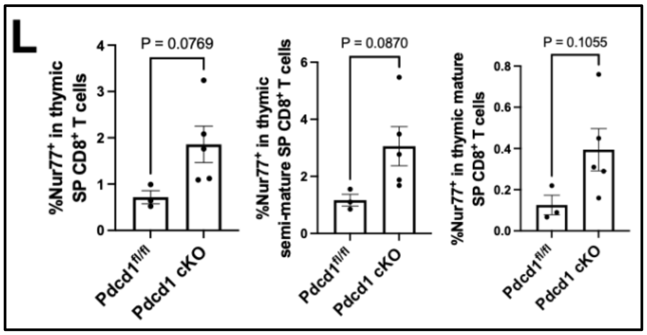

Extended Data Fig 1. Flow cytometry analysis of the thymic profile of single positive $CD8^+$ T cells.

(**L**) Frequencies of $Nur77^+$ in thymic total, semi-mature, and mature SP $CD8^+$ T cells in $Pdcd1^{fl/fl}$ (n=3) and Pdcd1 cKO (n=5) mice.

6) The human data on Figure 4 are interesting but again to my opinion over interpreted. Stating that they 'demonstrate that PD1 regulates thymic output of Teff cells that are tumor reactive in peripheral tissues' is clearly an overstatement. There is no more than a small correlation, as this is not well studied. The authors should either provide data that better support such bold statements or tone down. On the same note, I find Extended data 7 belonging to this figure very difficult to understand and interpret.

We appreciate the reviewer's critical comment on this point. To that end, we performed new TCR sequencing analysis in additional mice with B16-OVA tumor or B16-F10 tumor, along with their thymus. These new data further support that, thymic $CD8^+$ T cells shared TCR clonotypes with $CD8^+$ tumor-infiltrating lymphocytes (TILs) in the absence of PD-1 **(New Fig. 6A-C)**. In addition, we added new data using *RAG-GFP* reporter mice, demonstrating the presence of RTE within the tumor tissues **(New Fig. 6D-I)**. Per this reviewer's concern regarding overinterpretation, we revised our statement as "latent effector differentiation of thymic SP $CD8^+$ T cells can directly contribute to the TCR repertoire of TILs". In Extended data Figure 7, we provide the figure showing the original tumor section used for spatial transcriptomics. We also revised the figure labeling and legend to improve their clarity.

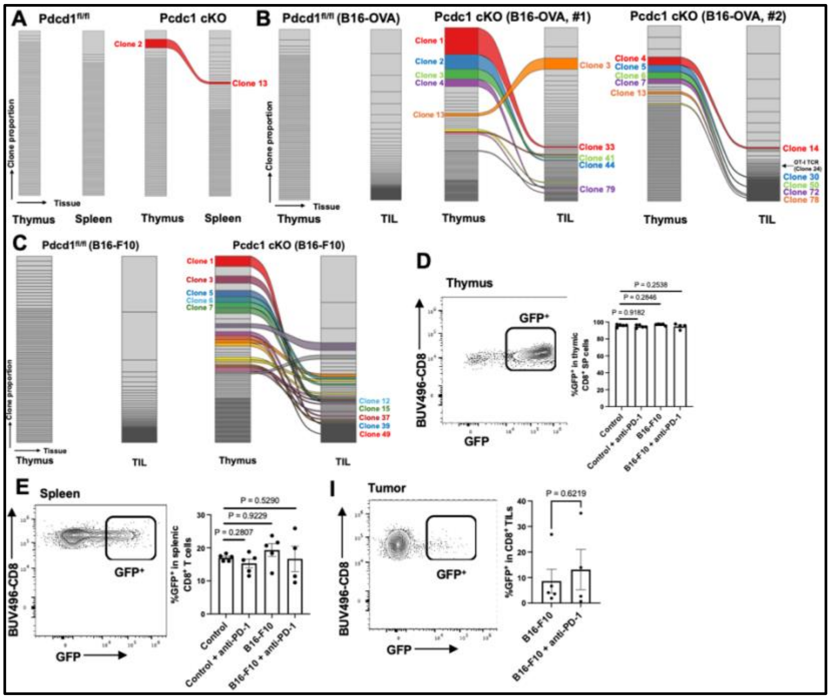

Figure 6. Shared CD8^{+} T cell clonotypes in the thymus and tumors in the absence of PD-1.
(**A–C**) TCR sequencing analysis comparing CD8^{+} T cell clonotypes across thymus, spleen, and tumors in Pdcd1$^{fl/fl}$ and Pdcd1 cKO mice. Arrow indicates TCR clone (#24) that is identical to OT-1 TCR.
(**A**) Comparison of CD8^{+} T cell clonotypes between thymus and spleen of Pdcd1$^{fl/fl}$ (left) and Pdcd1 cKO mice (right) at baseline.
(**B**) Comparison of CD8^{+} T cell clonotypes between thymus and CD8^{+} TILs of Pdcd1$^{fl/fl}$ (left), two representative Pdcd1 cKO mice (middle, right) on day 12 after B16-OVA tumor challenge.
(C) Comparison of CD8^{+} T cell clonotypes between thymus and CD8^{+} TILs of Pdcd1$^{fl/fl}$ (left) and Pdcd1 cKO mice (right) on day 12 post B16-F10 tumor challenge.
(**D-I**) Detection of GFP^{+} recent thymic emigrants in RAG-GFP knock-in mice at baseline and following B16-F10 tumor challenge, with or without anti-PD-1 blockade. Groups included baseline control (n=5), control + anti–PD-1 (n=5), B16-F10 tumor (n=5), and B16-F10 tumor + anti-PD-1 blockade (n=4). (**D**) Gating strategy and percentage of GFP^{+} CD8^{+} T cells in the thymus. (**E**) Gating strategy and percentage of GFP^{+} CD8^{+} T cells in the spleen. **(I**) Gating strategy and percentage of GFP^{+} CD8^{+} T cells in the tumor.

7) Figure 5 shows tumor take, not tumor clearance. For tumor clearance T cell transfer experiments are required. Overall, I again find the data difficult to interpret. Is it a numbers game of more effector T cells? As the cells probably will produce much more rapidly and more quantities of IFNg and other cytokines upon activation: how to they shape the other immune cells which will respond to this altered response? There is no evidence that the thymocytes in fact contribute to better killing etc (as already indicated for the model description in figure 1). Such data are also lacking here. I understand that the tumors do not take, rendering it more difficult to follow immune responses. However, they could at least follow (the tumor specific) T cell responses in the blood which can serve as a proxy of what is going on.

We appreciate this insightful comment. We changed the wording in the main text highlighting “tumor take”. Firstly, we added the percentage and total cell number of splenic CD8^{+} T cells after poly I:C/OVA immunization for 1 week, 2-week and 3-week (**New Extended Data Fig 9 I-K**). We found an increase in the percentage of CD8^{+} T cells and total CD8^{+} T cell number in the spleen in the absence of PD1 compared to the control, with the peak at 1 week after immunization. We also found an increased proliferative capacity of CD8^{+} T cells after immunization marked by Ki67 expression. In addition, we isolated control and *Pdcd1*-deficient thymic CD8+ T cells and performed a killing assay towards P815 target cells (**New Fig 7E)**. We observed a statistically significant decrease in the number of live target cells in culture with PD-1 deficient SP CD8^{+} T cells, indicating the SP CD8 thymocytes have the cytotoxic capacity that may contribute to tumor killing in the absence of PD-1 after immunization.

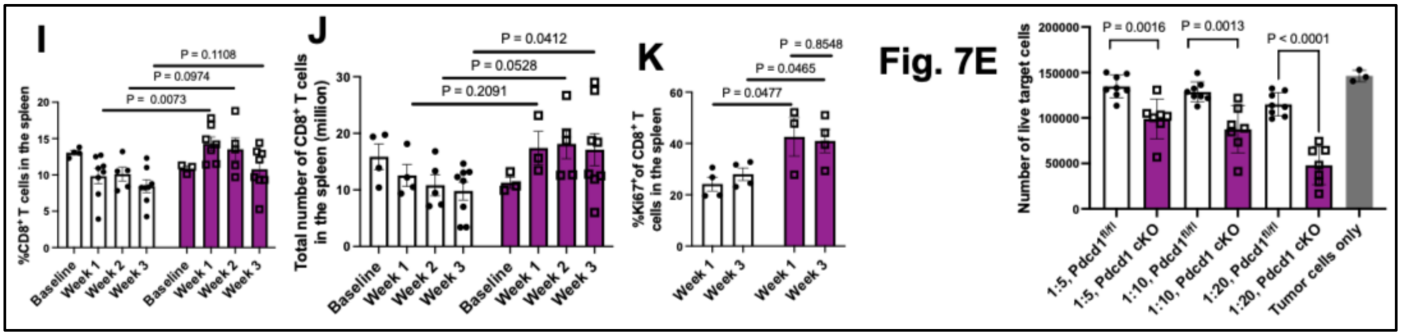

Extended Data Fig 9. Thymus and spleen phenotype of CD8+ T cells after poly(I:C)/OVA immunization. (**H**) Percentage of Ki67$^+$ in CD55$^+$CD24$^+$ splenic CD8$^+$ T cells after poly(I:C)/OVA immunization. (**I**) Percentage of splenic CD8+ T cells at baseline (n=4 and n=3) and following 1-week (n=8 and n=7), 2-week (n=5 and n=5) and 3-week (n=8 and n=8) poly(I:C)/OVA immunization in Pdcd1$^{fl/fl}$ and Pdcd1 cKO mice. (**J**) Total number count of splenic CD8+ T cells at baseline (n=4 and n=3) and following 1-week (n=4 and n=3), 2-week (n=5 and n=5) and 3-week (n=8 and n=8) poly(I:C)/OVA immunization in Pdcd1$^{fl/fl}$ and Pdcd1 cKO mice. (**K**) Frequency of Ki67$^+$ of splenic CD8+ T cells at 1-week (n=4 and n=3) and 3-week (n=4 and n=4) after immunization. **Fig 7(E).** Number of live P815 target cells after 48-hour co-culture with isolated thymic CD8$^+$ T cells from Pdcd1$^{fl/fl}$ (n=7) or CD8-Pdcd1 cKO (n=7) mice in the presence of IL-2 (40 IU/mL) and IL-15 (10 ng/mL) at target-to-effector ratios of 1:5, 1:10, and 1:20.

On the other hand, although we did not observe an overall change in a spectrum of immune cell profile including dendritic cells and B cells in the Pdcd1 cKO mice compared to control mice, we noticed a decrease in the frequency of CD4+ T cells and NK cells, whereas the frequency of macrophages were trending towards an increase after immunization in Pdcd1 cKO mice. We are adding these new data in **Extended Data Fig 9 O-T.**

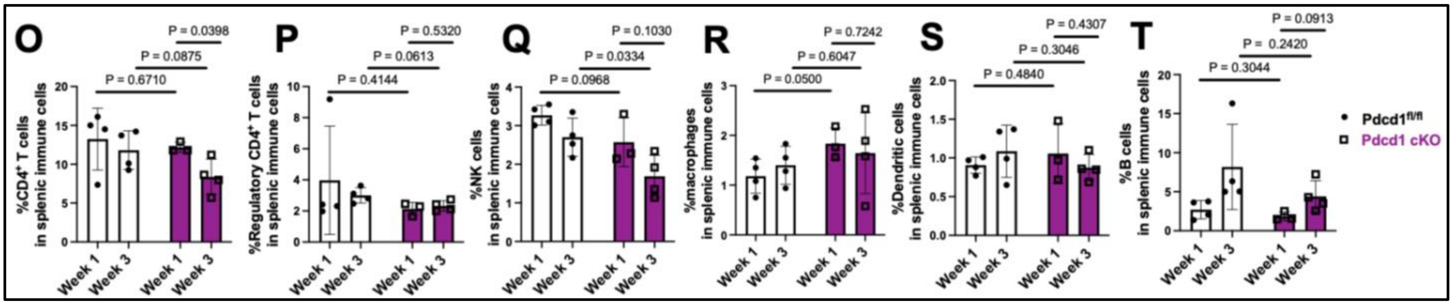

Extended Data Fig 9. Thymus and spleen phenotype of CD8+ T cells after poly(I:C)/OVA immunization. (**O-T**) Frequencies of CD4$^+$ T cells (**O**), Foxp3$^+$ regulatory CD4$^+$ T cells (**P**), NK cells (**Q**), TCRβ$^-$CD11b$^+$F4-80$^+$ macrophages (**R**), TCRβ$^-$CD11c$^+$dendritic cells (**S**), CD19$^+$B cells (**T**) in splenic CD45$^+$ immune cells after immunization.

To assess the contribution of thymocytes to tumor control, we tracked the tumor infiltration of recent thymic emigrants (RTEs), serving as a proxy for thymocytes, into tumor sites using *RAG-GFP* mice treated with or without anti-PD-1 therapy (**New Fig. 6D-I**). As expected, most GFP$^+$ cells were enriched in the thymus and some of them were detected in the spleens. Interestingly, GFP$^+$ (RTE) CD8$^+$ T cells were also identified within tumor tissues, suggesting that a recent thymic output of SP CD8$^+$ T cells may contribute to the tumor control. Of note, anti-PD-1 treatment did not affect the frequency of GFP$^+$ cells in both thymus and spleens. The phenotype of GFP$^+$ RTE CD8$^+$ T cells demonstrated feature of naïve-like (CD44$^-$CD62L$^+$) compared to GFP$^-$ non-RTE CD8$^+$ T cells within, which was affected by the anti-PD-1 therapy. Since anti-PD-1 treatment did not affect the frequency of GFP$^+$ RTE CD8$^+$ T cells within tumor sites, our data suggest that the increased effector phenotype of CD8$^+$ TILs in Pdcd1 mice could be pre-programmed by PD-1's intrinsic signals that may not be affected by anti-PD-1 antibody as reported (PMID: 40705010). To further test this possibility, we performed single-cell TCR sequencing among single-positive (SP) CD8$^+$ thymocytes and CD8$^+$ tumor-infiltrating lymphocytes (TILs) in

mice bearing B16-OVA or B16-F10 tumors. Our new data show that several TCR clones were shared between SP CD8$^+$ thymocytes and CD8$^+$ TILs only in PD-1 cKO mice, but not in control mice (**new Fig. 6B-C**). Taken together, our findings suggest the absence of intrinsic PD-1 signaling increases the thymic output of SP CD8$^+$ T cells that are capable of infiltrating peripheral tumor tissues.

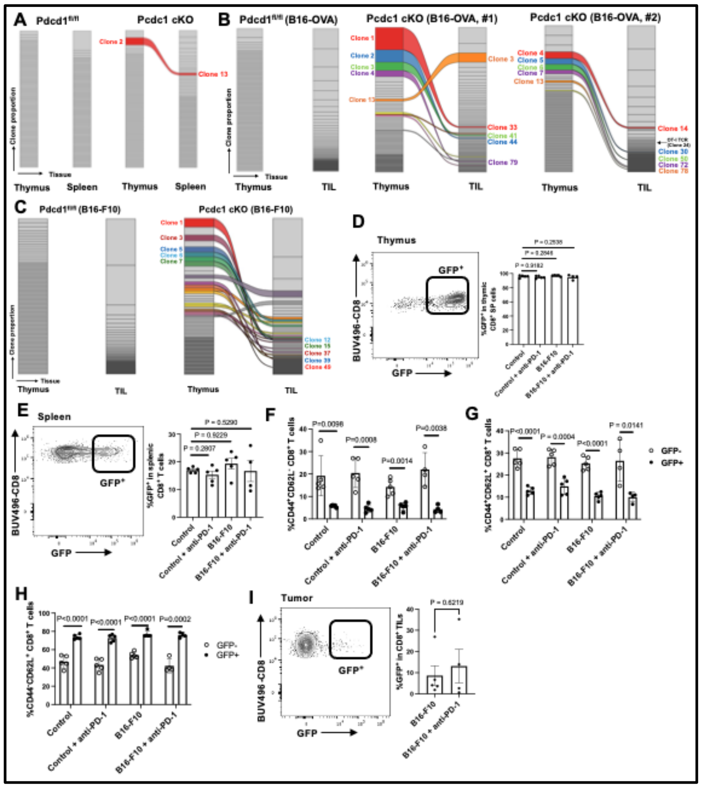

Figure 6. Shared CD8$^+$ T cell clonotypes in the thymus and tumors in the absence of PD-1. A–C) TCR sequencing analysis comparing CD8$^+$ T cell clonotypes across thymus, spleen, and tumors in Pdcd1$^{fl/fl}$ and Pdcd1 cKO mice. (**A**) Comparison of CD8$^+$ T cell clonotypes between thymus and spleen of Pdcd1$^{fl/fl}$ (left) and Pdcd1 cKO mice (right) at baseline. (**B**) Comparison of CD8$^+$ T cell clonotypes between thymus and CD8$^+$ TILs of Pdcd1$^{fl/fl}$ (left), two representative Pdcd1 cKO mice (middle, right) on day 12 after B16-OVA tumor challenge. Arrow indicates TCR clone that is identical to OT-1 TCR. (**C**) Comparison of CD8$^+$ T cell clonotypes between thymus and CD8$^+$ TILs of Pdcd1$^{fl/fl}$ (left) and Pdcd1 cKO mice (right) on day 12 post B16-F10 tumor challenge. (**D-I**) Detection of GFP$^+$ recent thymic emigrants in RAG-GFP knock-in mice at baseline and following B16-F10 tumor challenge, with or without anti-PD-1 blockade. Groups included baseline control (n=5), control + anti–PD-1 (n=5), B16-F10 tumor (n=5), and B16-F10 tumor + anti-PD-1 blockade (n=4). (**D**) Gating strategy and percentage of GFP$^+$ CD8$^+$ T cells in the thymus. (**E**) Gating strategy and percentage of GFP$^+$ CD8$^+$ T cells in the spleen. (**F-H**) Frequencies of GFP$^+$ and GFP$^-$ CD8$^+$ T cells within splenic effector-like CD44$^+$CD62L$^-$ (**F**), central memory-like CD44$^+$CD62L$^+$ (**G**) and CD44$^-$CD62L$^+$ naïve (**H**) subsets. (**I**) Gating strategy and percentage of GFP$^+$ CD8$^+$ T cells in the tumor.

8) The vaccination model already is better in studying the tumor responses. Yet again, it only studies tumor take and not the effect on tumor growth etc. It shows that T cells have the capacity to become memory cells- what type of memory cells? why are again T cell responses not followed? The authors lean in the manuscript too much on assumptions and deductions from their findings rather than confirming them experimentally.

We appreciate this critical comment. To investigate the generation of memory CD8$^+$ T cells following immunization, we performed phenotypic analyses over a 3-week period after poly(I:C)/OVA immunization. We observed a higher frequency of effector memory cells (CD44$^+$CD62L$^-$) and a proportional lower frequency of central memory (CD44$^+$CD62L$^-$) and naive (CD44$^-$CD62L$^+$) cells in the splenic CD8$^+$ T cells in PD-1 cKO mice compared to control mice (**New Extended Data Fig. 9L-M**). Within antigen-primed CD11a^{high} CD8+ T cell subsets, PD-1 cKO cells expressed higher levels of CX3CR1 and exhibited enhanced cytotoxic capacity (CD107a) in PD-1 cKO mice compared to the control mice (**new Fig.7 L-O**). Among the effector memory cells, we found the absence of PD-1 increased the expression of cytotoxic molecules (NKG7, Perforin, Granzyme B) and exhaustion-like markers, (TOX and TIM3) compared with control cells after immunization (**new Fig.7 P-T**). To further test the memory potential of these effector memory cells, we measured their expression of phosphorylated S6 (pS6)—a marker of mTORC1 activity known to regulate memory T cell differentiation. While the expression of pS6 had a trend of decrease in effector memory cells in PD-1 cKO mice compared to control mice (**New Extended Data Fig. 9N**), our results suggest that PD-1's intrinsic signals may marginally overlap with mTOR pathway in regulation of memory potential of CD8+ T cells after immunization.

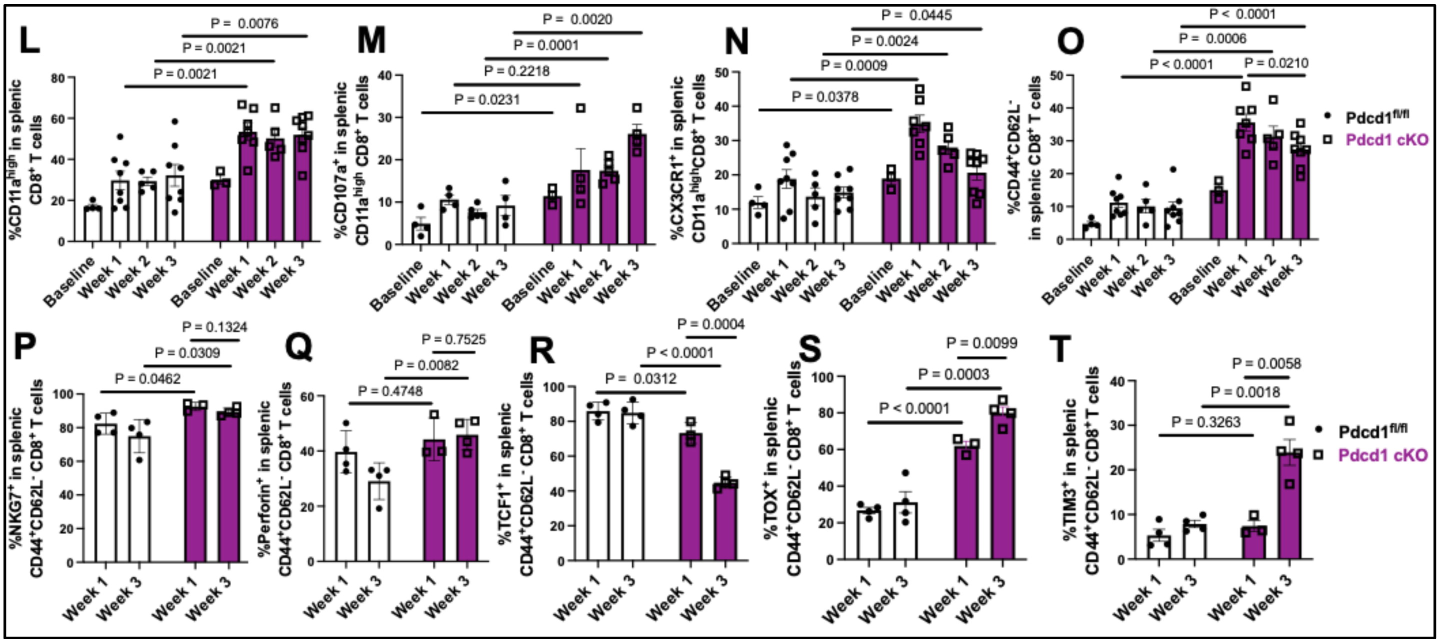

Figure 7. PD-1 deficiency distinctly shapes effector- and memory phase immunization responses in long-term anti-tumor immunity. (**L–T**) Peripheral CD8$^+$ T cell responses following poly(I:C)/OVA immunization. (**L**) Frequency of antigen-primed CD11a^{high} splenic CD8+ T cells at baseline (n=4 and n=3), 1-week (n=8 and n=7), 2-week (n=5 and n=5) and 3-week (n=8 and n=8) after immunization in Pdcd1$^{fl/fl}$ and Pdcd1 cKO mice. (**M**) Frequencies of CD107a$^+$ degranulation in splenic CD11a^{high} CD8$^+$ T cells after anti-CD3/CD28 beads stimulation for 48 hours at baseline (n=4 and n=3) and following 1 week (n=4 and n=3), 2-week (n=5 and n=5) and 3-week (n=4 and n=4) poly(I:C)/OVA immunization in Pdcd1$^{fl/fl}$ and Pdcd1 cKO mice. (**N**) Frequencies of CX3CR1$^+$ in splenic CD11a^{high} CD8+ T cells at baseline (n=4 and n=3), 1-week (n=8 and n=7), 2-week (n=5 and n=5) and 3-week (n=8 and n=8) after immunization in Pdcd1$^{fl/fl}$ and Pdcd1 cKO mice.
(**O**) Frequencies of effector-like CD44$^+$CD62L$^-$ splenic CD8$^+$ T cells after poly(I:C)/OVA immunization at baseline (n=4 and n=3) and following 1 week (n=8 and n=7), 2-week (n=5 and n=5) and 3-week (n=8 and n=8) immunization in Pdcd1$^{fl/fl}$ and Pdcd1 cKO mice. (**P-T**) Frequencies of NKG7$^+$ (**P**), Perforin$^+$ (**Q**), TCF1$^+$(**R**), TOX$^+$(**S**) and TIM3$^+$ (**T**) in splenic CD44$^+$CD62L$^-$ CD8$^+$ T cells at 1-week (n=4 and n=3) and 3-week (n=4 and n=4) poly(I:C)/OVA immunization in Pdcd1$^{fl/fl}$ and Pdcd1 cKO mice.

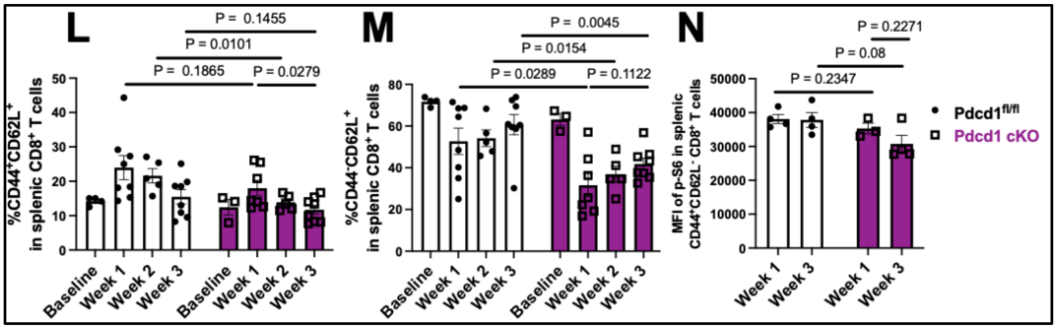

Extended Data Fig 9. Thymus and spleen phenotype of CD8$^+$ T cells after poly(I:C)/OVA immunization. (L, M) Percentage of CD44$^+$CD62L$^-$ (L) and CD44$^-$CD62L$^+$ (M) CD8$^+$ T cells at baseline and following 1-week (n=8 and n=7), 2-week (n=5 and n=5) and 3-week (n=8 and n=8) immunization in Pdcd1$^{fl/fl}$ and Pdcd1 cKO mice. (N) MFI of p-S6 in splenic effector-like CD44$^+$CD62L$^-$ splenic CD8$^+$ T cells after immunization.

9) Figure 3: the authors isolated and measured the TILs from the mice: again: what are the numbers of Tcells that infiltrate tumors (per gramm Tissue) from WT versus PD1 ko cells? what is their functional profile?

We provided new data showing the percentages of CD8$^+$ TILs was significantly increased in the absence of PD-1, whereas the total number of CD8$^+$ TILs per gram tumor tissues remained comparable between control and PD-1 cKO mice at day 12 post tumor injection (**new Fig.4E-F**). Our data suggest that PD-1 deficiency may promotes the functional CD8$^+$ T cells to retain within the tumor microenvironment (TME). To support this possibility, we found the frequency of MitoSOX$^+$ cells increased in Pdcd1-deficient CD8$^+$ TILs along with increased effector capacity (CD107a$^+$) cells compared to control cells, but their expression of phosphorylated S6 (pS6) decreased (**new Fig.4O, S, T**). Our data suggest that although CD8$^+$ TILs experience mitochondrial oxidative stress within TME as reflected by their expression of MitoSOX, more PD-1 deficient CD8$^+$ TILs can retain in TME due to their lower activity of mTOR (pS6) which is associated with less T cell exhaustion and better T cell longevity (PMID: 36378537, PMID: 39952956).

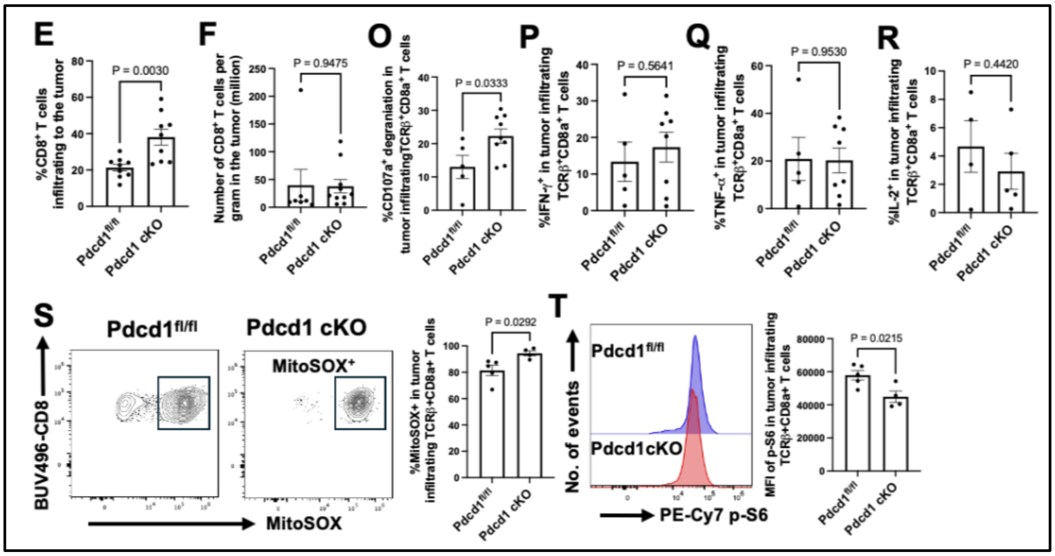

Figure 4. Tumor immunity is regulated by PD-1 according to the immunogenicity. (E) Percentage of CD8$^+$ TILs (n=9 and n=9). (F) Absolute number of CD8$^+$ TILs (n=7 and n=10). (O-R) Cytokine production and degranulation of CD8$^+$ TILs following 4-hour PMA and ionomycin stimulation at day 12 post B16-OVA tumor challenge: CD107a$^+$ degranulation (O, n=5 and n=9), IFN-γ^+ (P, n=5 and n=9), TNFα^+ (Q, n=5 and n=9) and IL-2$^+$ (R, n=4 and n=5). (S) Gating strategy (left) and frequency of MitoSOX$^+$ CD8$^+$ TILs (right) in Pdcd1$^{fl/fl}$ (n=5) and Pdcd1 cKO mice (n=4). (T) Gating strategy (left) and median fluorescence intensity (MFI) of phosphorylated S6 (p-S6) (right) in CD8$^+$ TILs in Pdcd1$^{fl/fl}$ (n=5) and Pdcd1 cKO mice (n=4). (A) is created using Biorender. P value was calculated using an unpaired, two-tailed t test.

10) Also their main conclusion that the thymocytes contribute to anti-tumoral responses and that aPD1 blocking can contribute to it seems an interesting hypothesis but at this stage not well founded by experiments

We appreciate these constructive comments. To support our main conclusion, we added additional mice challenged with B16-OVA and B16-F10 tumors. From there, we found TCR clonotypes in the tumors can be tracked back to thymus in the absence of PD-1 (**New Fig. 6A-C**). Furthermore, we incorporated additional experiments, including the use of *RAG-GFP* reporter mice to track recent thymic emigrants (RTEs) in tumor tissues with and without anti-PD-1 therapy (**New Fig. 6D-I**). Although there was no statistically significant difference in the percentages of GFP$^+$CD8$^+$ T cells between B16-F10 tumors treated with or without anti-PD-1 therapy, GFP$^+$ CD8$^+$ T cells were detected within the tumor, indicating CD8$^+$ thymocytes contributed to anti-tumor responses. While the reason is still not clear about why anti-PD-1 treatment did not change the RTE in tumor sites, it is possible that PD-1 blockade primarily promotes peripheral CD8$^+$ T cell activation, thymic PD-1 deficiency may intrinsically enhance the effector competence of CD8$^+$ T cells during their development.

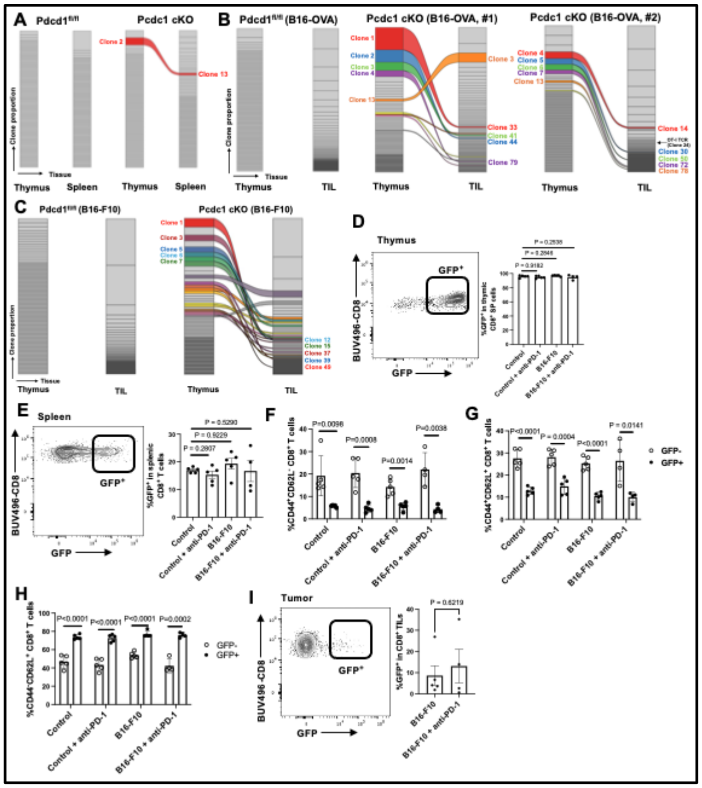

Figure 6. Shared CD8$^+$ T cell clonotypes in the thymus and tumors in the absence of PD-1. A–C) TCR sequencing analysis comparing CD8$^+$ T cell clonotypes across thymus, spleen, and tumors in Pdcd1$^{fl/fl}$ and Pdcd1 cKO mice. (**A**) Comparison of CD8$^+$ T cell clonotypes between thymus and spleen of Pdcd1$^{fl/fl}$ (left) and Pdcd1 cKO mice (right) at baseline. (**B**) Comparison of CD8$^+$ T cell clonotypes between thymus and CD8$^+$ TILs of Pdcd1$^{fl/fl}$ (left), two representative Pdcd1 cKO mice (middle, right) on day 12 after B16-OVA tumor challenge. Arrow indicates TCR clone that is identical to OT-1 TCR. (**C**) Comparison of CD8$^+$ T cell clonotypes between thymus and CD8$^+$ TILs of Pdcd1$^{fl/fl}$ (left) and Pdcd1 cKO mice (right) on day 12 post B16-F10 tumor challenge. (**D-I**) Detection of GFP$^+$ recent thymic emigrants in RAG-GFP knock-in mice at baseline and following B16-F10 tumor challenge, with or without anti-PD-1 blockade. Groups included baseline control (n=5), control + anti–PD-1 (n=5), B16-F10 tumor (n=5), and B16-F10 tumor + anti-PD-1 blockade (n=4). (**D**) Gating strategy and percentage of GFP$^+$ CD8$^+$ T cells in the thymus. (**E**) Gating strategy and percentage of GFP$^+$ CD8$^+$ T cells in the spleen. (**F-H**) Frequencies of GFP$^+$ and GFP$^-$ CD8$^+$ T cells within splenic effector-like CD44$^+$CD62L$^-$ (**F**), central memory-like CD44$^+$CD62L$^+$ (**G**) and CD44$^-$CD62L$^+$ naïve (**H**) subsets. (**I**) Gating strategy and percentage of GFP$^+$ CD8$^+$ T cells in the tumor.

Reviewer #2 (Remarks to the Author)

This manuscript "PD-1 limits thymic output of effector CD8+ T cells for peripheral tumor immunity" by Zhiming Mao and Colleagues uncovers a previously unappreciated population of total thymocytes in conditional PD-1 knockout mice. Using a combination of single-cell RNA sequencing, reporter genetics, and flow cytometry, the authors show that in the absence of PD 1, these cells acquire a full cytotoxic transcriptomic profile, challenging the classical view that the thymus lacks effector like CD8$^+$ differentiation. The authors present their findings in the context of checkpoint inhibition, showing that PD 1 deficient SP CD8$^+$ T cells infiltrate tumors more effectively, mediate early rejection or growth delay, and show enhanced effector molecule expression (e.g., Granzyme B, NKG7), but also increased expression of exhaustion markers (TOX, Havcr2) and ultimately lose cytotoxic function by day 21. These cells share TCR clonotypes with thymic cells, suggesting a thymic origin.

In my view, this study should not primarily be framed as a checkpoint-focused investigation. Its novelty lies in uncovering an aspect of thymic T cell development—specifically, the emergence of cytotoxic-like CD8$^+$ thymocytes under the control of PD-1. I strongly recommend reframing the manuscript to emphasize thymic T cell maturation and selection. This would more effectively highlight the mechanistic novelty of the findings and better align the work with the interests of a more relevant immunology-focused audience. Even with a revised focus, several major concerns remain.

We deeply appreciate this reviewer's comments and suggestions. We reframed our manuscript to align with the discovery of the emergence of cytotoxic-like CD8$^+$ thymocytes under the control of PD-1. Accordingly, we revised our title as: **PD-1 regulates latent effector differentiation of thymic cytotoxic CD8$^+$ T cells.**

Major Concerns

Extended Data Fig 1 F. Authors show that PD-1 knockout was achieved. The percentage of PD-1–positive cells in knockout mice remains at approximately 7%. How reliable is the knockout under these conditions? A general defect in T cells of cKO has to be ruled out. It would help to see as control staining of Eomes, Perforin… side-by-side using naive CD8 T cells from the periphery of the same mouse (plus FMO controls showing the FACS plots). Was the absence of PD-1 confirmed in the sequencing data?

These are important questions for a clarification. Following our confirmation of the absence of PD-1 protein in $CD8^+$ T cells by flow cytometry (**Extended Data Fig. 1F**), we performed the single-cell RNA sequencing of $CD8^+$ T cells from PD-1 cKO mice. Although we did not find a total loss of Pdcd1 transcript (**Fig. 1D**), the discrepancy between the loss of PD-1 protein and remaining PD-1 transcription could be explained as: the E8I-Cre recombinase only deletes exons 2 and 3 of the Pdcd1 gene, which are flanked by LoxP sites in introns 1 and 3. Consequently, the remained exon 1 that encodes the 5′ UTR and signal peptide, and exons 4–5 that encode the intracellular domain of PD-1, may contribute to the partial transcription of Pdcd1 that can be detected by scRNA-seq, but is not be enough for protein translation. As suggested, we performed flow cytometry analysis of PD-1 cKO $CD8^+$ T cells side by side between naive and effector cells in the same mouse using the markers as mentioned (**new Extended Data Fig.4**). We found that peripheral naïve $CD8^+$ T cells did not spontaneously upregulate effector or exhaustion markers compared peripheral effector $CD8^+$ T cells in the same PD-1 conditional knockout (cKO) mice, suggesting there is no general defect in $CD8^+$ T cells in the absence of PD-1.

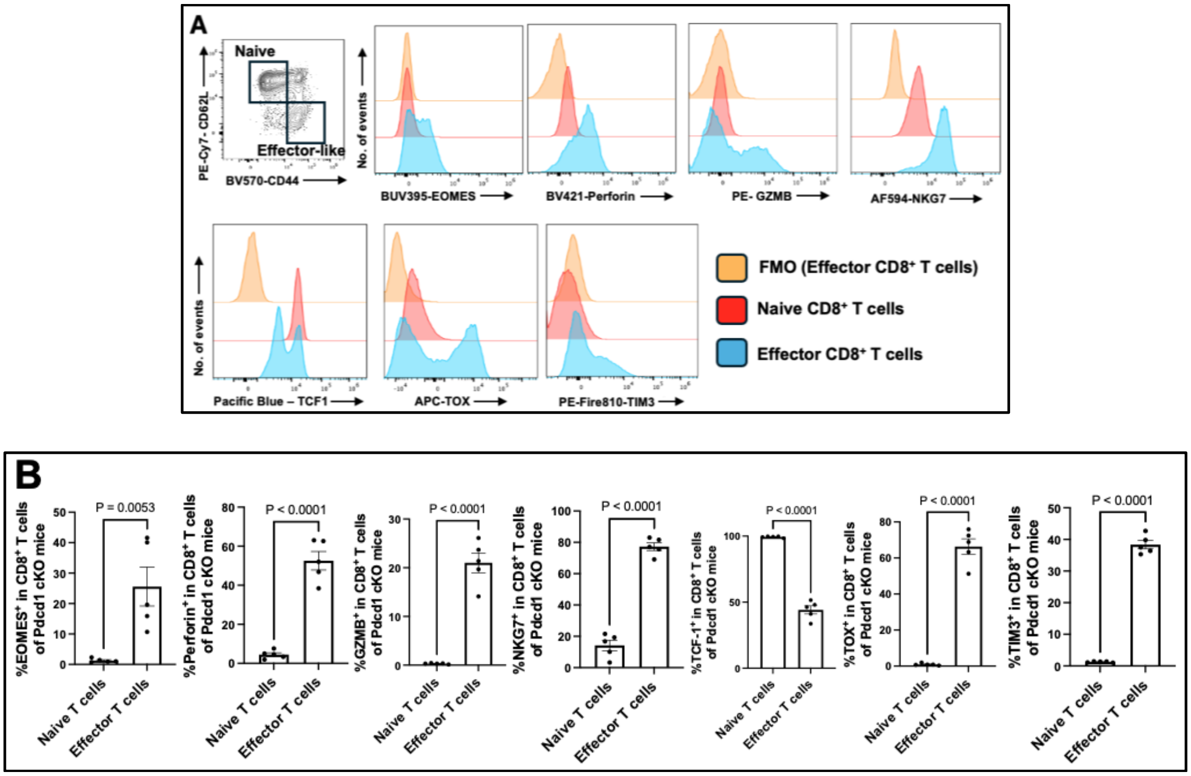

Extended Data Fig 4. Cytotoxic effector marker expression in naïve T cells and effector-like splenic $CD8^+$ T cells at baseline in the absence of PD-1. (A) Contour plot showing naïve and effector-like splenic CD8+ T cells followed by histograms showing the protein expression of EOMES, Perforin, GZMB, NKG7, TCF1, TOX and TIM3 in these cells using FMO as control in Pdcd1 cKO mice (n=5). (**B**) Frequencies of $EOMES^+$, $Perforin^+$, $GZMB^+$, $NKG7^+$, $TCF1^+$, TOX^+ and $TIM3^+$ in naïve $CD8^+$ T cells and effector-like $CD8^+$ T cells in Pdcd1 cKO mice (n=5)**.** P value was calculated using an unpaired, two-tailed t test.

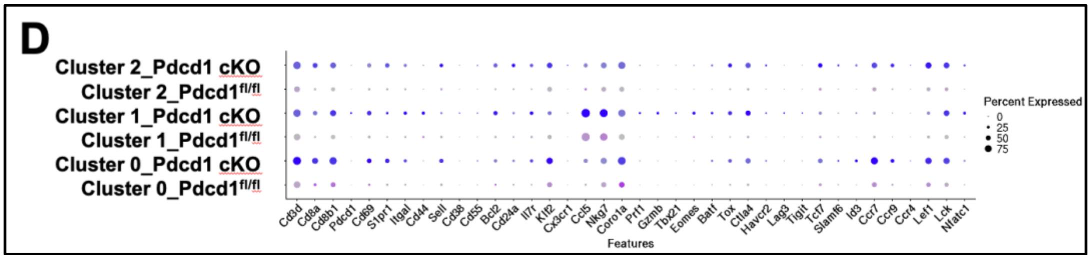

Figure 1. The absence of PD-1 increases the transcriptional and protein expression of cytotoxic effector-associated genes in thymic SP CD8$^+$ T cells. (D) Dotplot summarizing the expression of selected genes associated with cytotoxic effector, stem-like features, and TCR signaling across identified three clusters, stratified by Pdcd1 cKO and Pdcd1$^{fl/fl}$ mice. Dot color indicates the average expression level, with darker colors indicating higher expression (blue: Pdcd1 cKO; purple: Pdcd1$^{fl/fl}$). Dot size represents the percentage of cells expressing each gene within a cluster.

Is PD-1 just lower expressed (deficient) or is it a complete knockout? Is the knockout stable over time, or can PD-1 expression re-emerge in these cells? What level of off-target effects can be expected from the knockout strain used? Given that PD-1 regulates a variety of pathways, it remains unclear whether the observed effects are specifically due to PD-1 deficiency or reflect broader dysregulation of immune signaling. According to the signaling pathway analysis, immune-related pathways appear strongly affected, while pathways involved in protein synthesis and processing are downregulated. This raises the question to what extent the phenotypes can be attributed directly to PD-1 loss, or whether they result from secondary effects.

We believe PD-1 expression at least at protein levels is completely knocked out in CD8$^+$ T cells in our model. The deletion is mediated by E8I-Cre, which induces a permanent, DNA-level recombination event that is irreversible in affected cells. Consistent with this, we have not observed re-expression of PD-1 protein at any time point in Pdcd1-deficient CD8$^+$ T cells, including tumor-infiltrating CD8$^+$ T cells, as confirmed by flow cytometry (**New Fig. 4G**).

Regarding potential off-target effects, we consider them minimal for the following reasons:

1. Lineage specificity of E8I-Cre: PD-1 deletion and subsequent effector gene expression (e.g., NKG7) were not observed in double-positive (DP) or CD4 single-positive (SP) thymocytes (**New Extended Data Fig. 1I, J**), suggesting that E8I-Cre activity is restricted to the CD8 lineage without leakage into other thymocyte populations.
2. Peripheral T cell homeostasis: We did not detect changes in total number of CD8$^+$ T cells, despite an increased frequency of peripheral CD8$^+$ T cells and increased proliferative capacity (Ki67$^+$), indicating that E8I-Cre-mediated deletion of PD-1 does not broadly disrupt T cell biology (**New Fig. 3A-C**).
3. Functional validation: The observed phenotype of PD-1 deficiency CD8$^+$ T cells aligns with the functional consequences of PD-1 signaling in regulating effector CD8$^+$ T cells.

Although there are no published studies directly linking PD-1 to protein synthesis or processing pathways, our data suggest a potential new direction in PD-1 biology. Specifically, the absence of PD-1 appears to lead to downregulation of transcriptional programs associated with protein synthesis and processing. We interpret this as a reflection of T cell differentiation toward terminal effector states, which are characterized by reduced capacity for self-renewal.

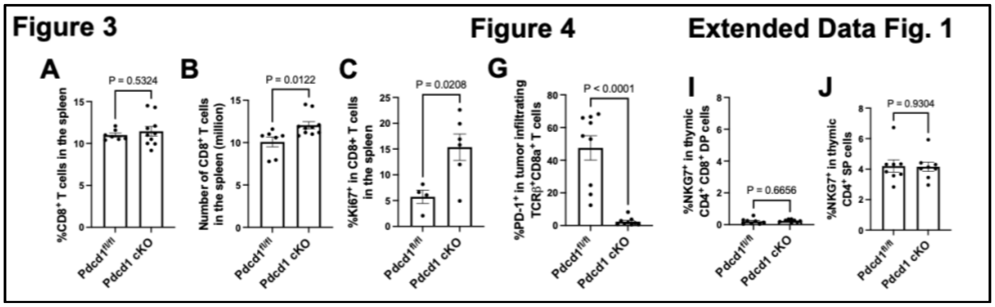

Figure 3. Increased cytotoxic potential of peripheral effector $CD8^+$ cells in the absence of PD-1. (A-C) Frequencies, absolute numbers, and proliferative capacity of splenic $CD8^+$ T cells in $Pdcd1^{fl/fl}$ (n=7) and Pdcd1 cKO (n=10) mice.
Figure 4. Tumor immunity is regulated by PD-1 to the immunogenicity. (G-N) Frequencies of $PD\text{-}1^+$ (G, n=10 and n=9) in CD8+ TILs isolated at day 12 post B16-OVA tumor injection.
Extended Data Fig 1. Flow cytometry analysis of the thymic profile of single positive $CD8^+$ T cells. (I, J) Frequency of $NKG7^+$ in thymic $CD4^+CD8^+$ double positive (DP) cells (I) and $CD4^+$ SP cells (J) in $Pdcd1^{fl/fl}$ (n=11) and Pdcd1 cKO (n=12) mice.

Fig.1 E-J. and Text. Flow cytometry analysis of thymic SP CD8 T cells after isolation show increased expression of cytotoxic and exhaustion markers in PD1 cKO mice. Comment: What are the possible reasons for the presence of cytotoxic cells in the thymus? What mechanisms or factors account for the fact that these cells already exhibit activation and enhanced cytotoxic potential as well as exhaustion like features?

That is a wonderful and insightful question that prompted us to explore recent literature. Since we observed that NKG7 is upregulated in SP $CD8^+$ T cells in the thymus, which exhibit cytotoxic potential, we used NKG7 as a keyword to search for articles addressing thymic development with any cytotoxic potential. Interestingly, a study by Steier et al. (Nat Immunol, 2023; PMID: 37580604) also reported NKG7 transcription in mature $CD8^+$ T cells within the thymus, although they did not further investigate the cytotoxic potential of these cells. Notably, they identified a second wave of TCR signaling that occurs specifically in CD8-fated thymocytes during lineage commitment. Based on these findings, we hypothesize that this second TCR signaling wave may not only drive $CD8^+$ T cell lineage commitment but also induce cytotoxic capacity, as evidenced by NKG7 expression. We measured the expression of Nur77, a specific and sensitive marker of TCR signals, in thymic SP $CD8^+$ T cells. We found that the frequency of $Nur77^+$ cells were modestly increased in the absence of PD-1 within semi-mature T cells (**Extended Data Fig. 1L**). These results suggest that latent effector differentiation may be initiated by TCR signals following positive selection but is restrained by PD-1 signaling at this stage in thymus to prevent newly generated cytotoxic $CD8^+$ T cells from causing nonspecific damage to healthy tissues prior to encountering pathogenic or malignant antigens. Also, Kalia, et al (PMID: 34644150) reported that PD-1 deficiency in peripheral mature $CD8^+$ T cells does not affect effector programing during acute infection. Therefore, thymic SP $CD8^+$ T cells may be uniquely sensitive to intrinsic PD-1-mediated regulation of their cytotoxic capacity.

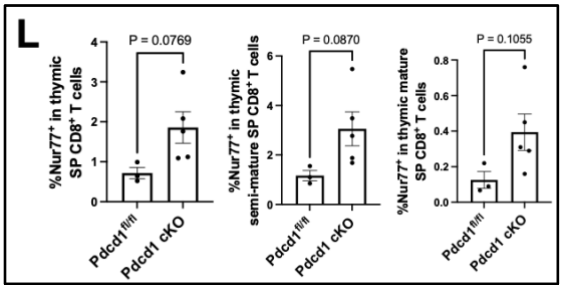

Extended Data Fig 1. Flow cytometry analysis of the thymic profile of single positive $CD8^+$ T cells. (L) Frequencies of $Nur77^+$ in thymic total, semi-mature, and mature SP $CD8^+$ T cells in $Pdcd1^{fl/fl}$ (n=3) and Pdcd1 cKO (n=5) mice.

Extended Data Fig. 2D: Core TCR signalling and protein synthesis pathways are down regulated in PD 1 / SP CD8 T cells—how can these cells still mount effective antigen processing and cytotoxic function under such impairment? How reliable are the conclusions about their cytotoxic capacity when central pathways required for T cell activation and function appear inhibited? A ex vivo killing assay would further strengthen the central claim that these thymic cells are bona fide functional effectors.

Again, we greatly appreciate this insightful question. As discussed above, the absence of PD-1 appears to lead to downregulation of transcriptional programs associated with protein synthesis and processing. We interpret this

as indicative of T cell differentiation toward terminal effector states, which are typically characterized by a diminished capacity for self-renewal. To investigate whether latent effector differentiation would produce virtual effector cells that are capable of killing tumor cells, we performed an *ex vivo* thymocyte-mediated cytotoxicity assay, in which freshly isolated thymic CD8^{+} cells were co-cultured with P815 target cells for 48 hours. Thymic CD8^{+} cells from Pdcd1 cKO mice exhibited increased killing activity compared with controls, which were more pronounced with increasing effector-to-target ratios (**new Fig. 2E**).

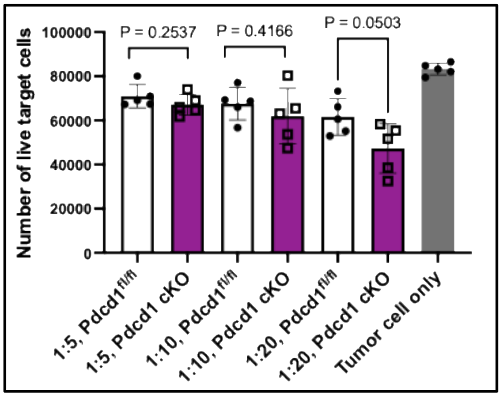

Figure 2. Thymic SP CD8^{+} T cells exhibited cytotoxic effector function in the absence of PD-1. (E) Number of live target P815 cells after 48-hour co-culture with isolated thymic CD8^{+} T cells from Pdcd1$^{fl/fl}$ (n=5) and Pdcd1 cKO (n=5) at the target-to-effector ratios of 1:5, 1:10 and 1:20.

Fig. 2 N–O. The sequencing tracks show higher Perforin, Granzyme B, Havcr2 and Tcf7 expression in PD 1 / cells, but it is unclear whether all displayed differences were formally tested. Please provide the statistical workflow—test applied, exact p values and FDR adjusted q values—for each gene. Without these metrics, several of the visual shifts could fall within noise and may not be biologically meaningful.

We appreciate these critical questions and the request for clarification regarding our observations. We have included the statistical analysis of signature gene expression in each cluster with both p values and FDR adjusted q values in (**New Extended Data Fig. 5F**). These genes are formally tested using Wilcoxon rank-sum test, and the exact p-value and FDR adjusted q values (p_val_adj) for the genes displayed in the Dotplot for cluster 5 are highlighted in **New Extended Data Fig. 5F**. We found that cytotoxic effector molecules such as Perforin, Granzyme B, Tox, Eomes and Tbx21 exhibited statistically significant differences in cluster 5 ($p < 0.05$) compared with other clusters, whereas Havcr2 and Tigit had statistically significant p values ($p < 0.05$) but did not reach significant difference after FDR adjustment (p_val_adj >0.05). This analysis revealed that the expression levels of key cytotoxic-related genes are significantly altered in the absence of PD-1 compared to control group, supporting our hypothesis about PD-1's regulatory role in modulating cytotoxic capacity of CD8^{+} T cells.

	p_val	avg_log2FC	pct.1	pct.2	p_val_adj	Cluster
Nkg7	1.98828487450551e-246	1.73382912	0.891	0.512	3.32997950782183e-242	5
Rgs1	1.85259144821737e-201	2.44180573	0.543	0.171	3.10272015747445e-197	5
Cd82	3.64504645068329e-121	2.09080079	0.33	0.09	6.10472379560437e-117	5
Bhlhe40	3.02099226148114e-107	1.98900936	0.315	0.09	5.05955783952861e-103	5
S100a11	1.25558241815149e-105	1.82188012	0.371	0.122	2.10284943392011e-101	5
Cd7	2.40655443743325e-103	2.01759094	0.348	0.113	4.03049737181321e-99	5
Prf1	1.04913466172465e-27	1.84287327	0.082	0.023	1.75709073145645e-23	5
Gzmb	5.19823333143861e-100	3.30817295	0.122	0.015	8.70600118349339e-96	5
Tox	1.82873350774494e-54	1.72262774	0.196	0.062	3.06276287877122e-50	5
Havcr2	4.83987440242106e-06	1.4469446	0.027	0.01	0.081058216	5
Tigit	8.02012097415379e-06	1.7843441	0.015	0.004	0.134320986	5
Eomes	1.34982479373736e-53	1.80768214	0.184	0.056	2.26068656455133e-49	5
Tbx21	7.64689295679068e-11	1.47094764	0.055	0.022	1.2807016324033e-06	5

Extended Data Fig 5. Single cell RNA-seq analysis of splenic CD8^{+} T cells. (**F**) the exact p-value and FDR adjusted q values (p_val_adj) for the featured cytotoxic-associated genes displayed in the Dotplot for cluster 5 are highlighted in **Fig. 3T**.

Extended Data Fig 3. To exclude that increased cytotoxic levels in thymic SP CD8 T cells are not recirculated mature CD8 cells, CD45 labeling was performed. This is nicely shown. This essential data belongs to the main figure part.

Thank you for the suggestion—we've now moved this figure to the main figure section (**new Fig. 2A-B**).

Fig 3 PD-1 knockout CD8 T cells exhibit enhanced effector function in vivo, as demonstrated by improved tumor control and greater tumor cell elimination. However, this effect appears restricted to tumors with high or intermediate immunogenicity, indicating that sufficient antigenic stimulation is required to unleash the full potential of these cells. By day 12, PD-1-deficient CD8 T cells show increased expression of exhaustion markers alongside an effector memory phenotype. This coincides with sustained upregulation of cytotoxic molecules such as Granzyme B and Perforin, suggesting that heightened effector activity is accompanied by progressive functional exhaustion.
Comment: Following immunization, are more recent thymic emigrants (RTEs) retained in the thymus to prime SP CD8 T cells with the specific peptide? Additionally, what is the role and phenotype of splenic CD8 T cells during this phase? Did the authors investigate whether the thymus still harbors mature, naïve T cells that could be mobilized and activated? Alternatively, could the early strong response result in a lymphopenic state, leaving insufficient numbers of functional T cells for sustained tumor control? This interpretation is supported by the observation in lines 143–144 that "mature peripheral CD8^{+} T cells were reduced in frequency". What are the implications of this decline for the long-term efficacy of PD-1-deficient CD8 T cells in anti-tumor immunity? What underlies this loss of function within the T cell pool by day 21?

We appreciate these insightful questions. To address them, we measured the frequency of thymic recirculating CD55^{+}H2-Kb^{+}CD8^{+} T cells following poly(I:C)/OVA immunization at 1 and 3 weeks. Our new data (**New Fig. 7F, G**) demonstrated that, in the absence of PD-1, the percentage of CD55^{+}H2-Kb^{+} thymic SP CD8^{+} T cells increased at both week 1 and week 3 after immunization, although their proliferative capacity declines by week 3 post-immunization. We also found a trend towards increase in the CD55^{+}CD24^{+}splenic RTE following immunization (**new Extended Data Fig. 9G, H**). These findings suggest that the thymus continues to harbor mature T cells capable of mobilization and activation.

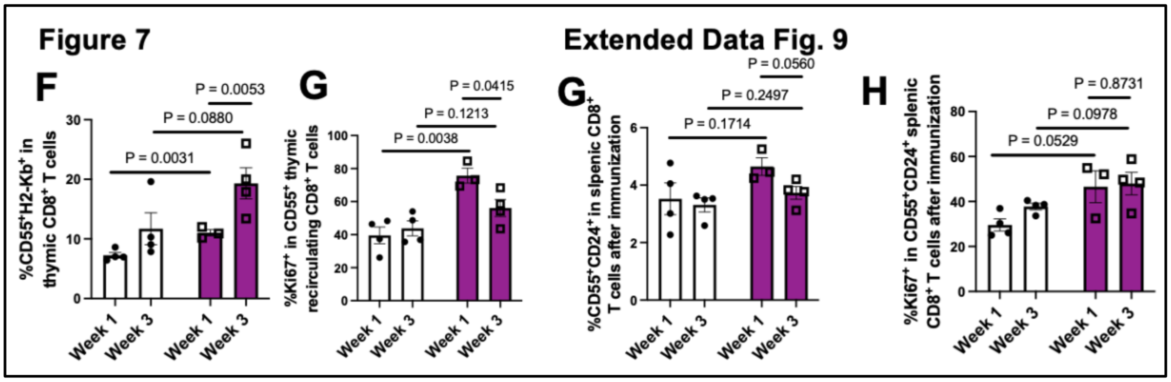

Figure 7. PD-1 deficiency distinctly shapes effector- and memory phase immunization responses in long-term anti-tumor immunity. (F) Frequency of thymic recirculating CD55^{+}H2-K^{b+} SP CD8^{+} T cells in Pdcd1$^{fl/fl}$ and Pdcd1 cKO mice after poly(I:C)/OVA immunization for one week (n=4 and n=3) and three weeks (n=4 and n=4). (G) Frequency of Ki67^{+} thymic recirculating CD55^{+} SP CD8^{+} T cells in Pdcd1$^{fl/fl}$ and Pdcd1 cKO mice after poly(I:C)/OVA immunization for one week and three weeks.

Extended Data Fig 9. Thymus and spleen phenotype of CD8^{+} T cells after poly(I:C)/OVA immunization. **(G)** Frequencies of CD55^{+}CD24^{+} RTE in splenic CD8+ T cells after poly(I:C)/OVA immunization. **(H)** Percentage of Ki67^{+} in CD55^{+}CD24^{+} splenic CD8^{+} T cells after poly(I:C)/OVA immunization.

We further profiled thymic SP CD8$^+$ T cells after immunization and found that Pdcd1-deficient CD8$^+$ T cells continued to generate cells with effector features, including upregulation of NKG7, Perforin, TIM3 and downregulation of TCF1 in both thymic CD55$^+$ recirculating and CD55$^-$ non-recirculating SP CD8$^+$ T cells (**New Fig. 9H-K**), accompanied by increased Ki67 proliferative capacity and enhanced *ex vivo* killing activity towards P815 target cells (**New Fig. 9E, Extended Data Fig. 9A-D**), suggesting thymus still harbor mature CD8$^+$ T cells that can be activated to kill target cells. Notably, we observed a significant increase in the phosphorylated S6 (p-S6) expression (a marker of mTORC1 activation) at three weeks compared with one week after immunization in the absence of PD-1, indicating a sustained metabolically active state (**New Extended Data Fig. 9E-F**).

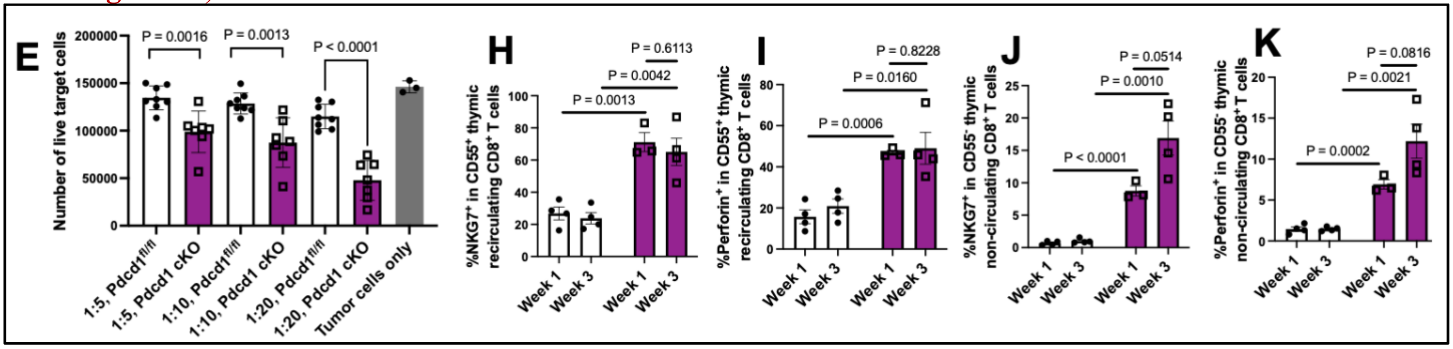

Figure 7. PD-1 deficiency distinctly shapes effector- and memory phase immunization responses in long-term anti-tumor immunity. (**E**) Number of live P815 mastocytoma target cells after 48-hour co-culture with isolated thymic CD8$^+$ T cells from Pdcd1$^{fl/fl}$ (n=7) or CD8-Pdcd1 cKO (n=7) mice in the presence of IL-2 (40 IU/mL) and IL-15 (10 ng/mL) at target-to-effector ratios of 1:5, 1:10, and 1:20.

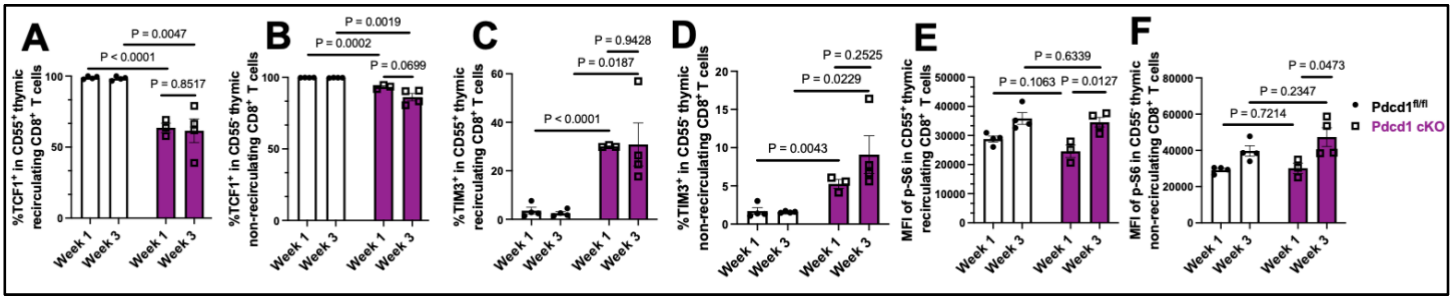

Extended Data Fig 9. Thymus and spleen phenotype of CD8$^+$ T cells after poly(I:C)/OVA immunization. (A, B) Frequencies of TCF1$^+$ in CD55$^+$ thymic recirculating CD8$^+$ T cells **(A)** and CD55$^-$ thymic non-recirculating **(B)** CD8$^+$ T cells at 1-week (n=4 and n=3) and 3-week (n=4 and n=3) after poly(I:C)/OVA immunization in Pdcd1$^{fl/fl}$ and Pdcd1 cKO mice. **(C, D)** Frequencies of TIM3$^+$ in CD55$^+$ thymic recirculating CD8$^+$ T cells **(C)** and CD55$^-$ thymic non-recirculating **(D)** CD8$^+$ T cells at 1-week and 3-week after poly(I:C)/OVA immunization. (**E, F**) Median fluorescent intensity (MFI) of p-S6 in CD55$^+$ thymic recirculating **(E)** and CD55$^-$ non-recirculating **(F)** CD8+ T cells at 1-week and 3-week after poly(I:C)/OVA immunization.

In terms of splenic CD8$^+$ T cell compartment during this phase, we observed a decrease in the naïve (CD44$^-$CD62L$^+$) population and an increase in both total cell number and the proportion of effector and effector memory cells (CD44$^+$CD62L$^-$, CX3CR1$^+$) by 21 days post-immunization, with the response peaking around one week after immunization in the absence of PD-1 (**New Fig.7N-O, Extended Data Fig. 9 I, J, L, M**).

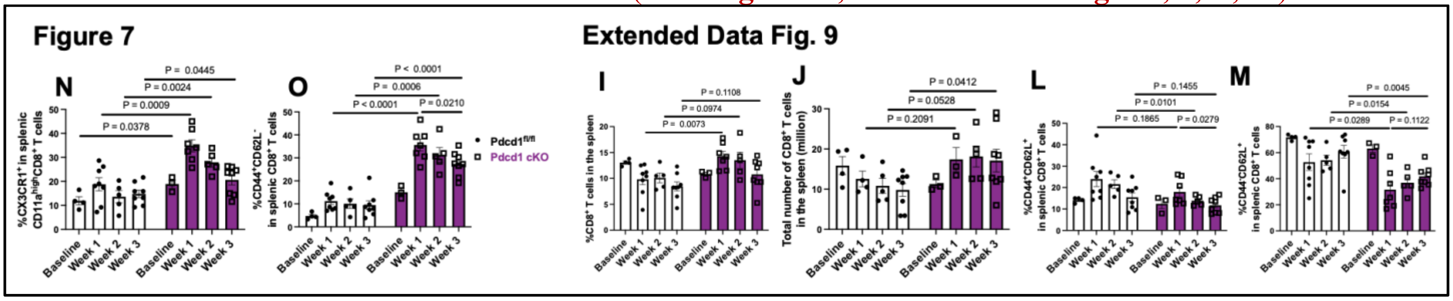

Figure 7. PD-1 deficiency distinctly shapes effector- and memory phase immunization responses in long-term anti-tumor immunity. (N) Frequencies of CX3CR1^{+} in splenic CD11a^{high} CD8+ T cells at baseline (n=4 and n=3), 1-week (n=8 and n=7), 2-week (n=5 and n=5) and 3-week (n=8 and n=8) after immunization in Pdcd1$^{fl/fl}$ and Pdcd1 cKO mice. (O) Frequencies of effector-like CD44^{+}CD62L^{-} splenic CD8^{+} T cells after poly(I:C)/OVA immunization at baseline (n=4 and n=3) and following 1 week (n=8 and n=7), 2-week (n=5 and n=5) and 3-week (n=8 and n=8) immunization in Pdcd1$^{fl/fl}$ and Pdcd1 cKO mice.

Extended Data Fig 9. Thymus and spleen phenotype of CD8^{+} T cells after poly(I:C)/OVA immunization. (**I**) Percentage of splenic CD8+ T cells at baseline (n=4 and n=3) and following 1-week (n=8 and n=7), 2-week (n=5 and n=5) and 3-week (n=8 and n=8) poly(I:C)/OVA immunization in Pdcd1$^{fl/fl}$ and Pdcd1 cKO mice. (**J**) Total number count of splenic CD8+ T cells at baseline (n=4 and n=3) and following 1-week (n=4 and n=3), 2-week (n=5 and n=5) and 3-week (n=8 and n=8) poly(I:C)/OVA immunization in Pdcd1$^{fl/fl}$ and Pdcd1 cKO mice. (**L, M**) Percentage of CD44^{+}CD62L^{-} (**L**) and CD44^{-}CD62L^{+} (**M**) CD8^{+} T cells at baseline and following 1-week (n=8 and n=7), 2-week (n=5 and n=5) and 3-week (n=8 and n=8) immunization in Pdcd1$^{fl/fl}$ and Pdcd1 cKO mice.

However, as the reviewer rightly pointed out, a robust early response may lead to attrition of the functional peripheral CD8^{+} T cell pool, which is essential for sustaining long-term antitumor immunity. One possible mechanism underlying this loss of function is the attrition of memory-like CD8^{+} T cells, which depend on PD-1 signaling for their maintenance. Supporting this, a study by Kalia et al. (PMID: 34644150) demonstrated that PD-1 expression is critical for the development and homeostatic renewal of memory CD8^{+} T cells following antigen clearance. Loss of PD-1 signaling led to increased contraction of the memory pool and impaired long-term maintenance. Mechanistically, PD-1 signaling helps balance mTOR activation and fatty acid oxidation, and its absence renders CD8^{+} T cells more susceptible to oxidative stress. To further investigate this, we performed new experiments showing that PD-1 deficiency results in increased mitochondrial ROS accumulation and decreased p-S6 in tumor-infiltrating CD8^{+} T cells in PD-1 cKO mice compared to control mice (**New Fig 4. S-T**).

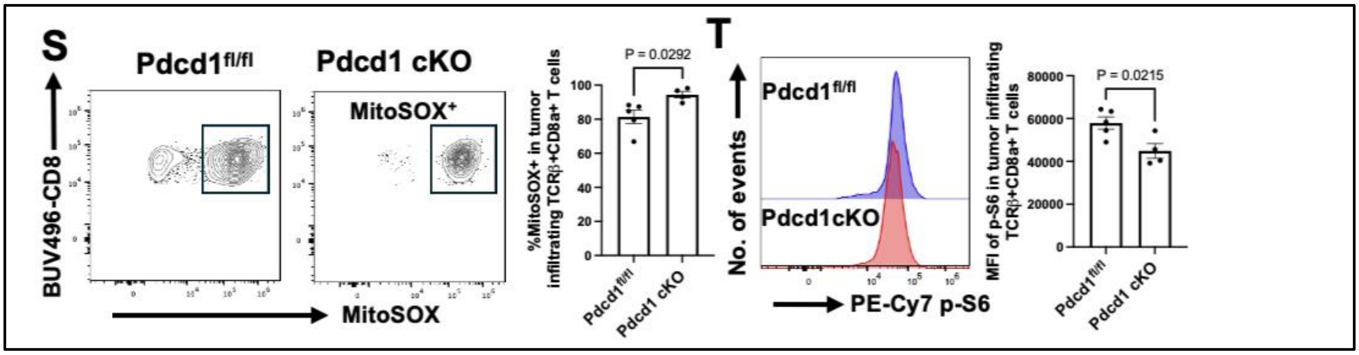

Figure 4. Tumor immunity is regulated by PD-1 to the immunogenicity. (S) Gating strategy (left) and frequency of MitoSOX^{+} CD8^{+} TILs (right) in Pdcd1$^{fl/fl}$ (n=5) and Pdcd1 cKO mice (n=4). (T) Gating strategy (left) and median fluorescence intensity (MFI) of phosphorylated S6 (p-S6) (right) in CD8^{+} TILs in Pdcd1$^{fl/fl}$ (n=5) and Pdcd1 cKO mice (n=4). (A) is created using Biorender. P value was calculated using an unpaired, two-tailed t test.

We also measured the p-S6 expression in effector memory CD44^{+}CD62L^{-} splenic CD8^{+} T cells after immunization. We observed a trend towards decrease in MFI of p-S6 expression at day 21 compared with day 7 in Pdcd1-deficient splenic CD8+ T cells after immunization (**Extended Data Fig. 9N**), suggesting impaired metabolic fitness that may contribute to defects in long-term memory and anti-tumor immunity.

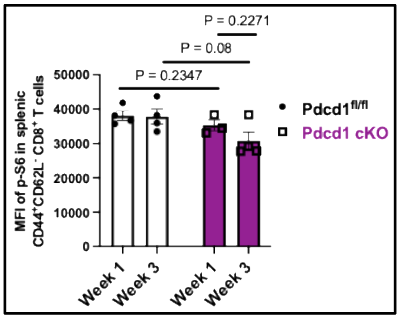

Extended Data Fig 9. Thymus and spleen phenotype of $CD8^+$ T cells after poly(I:C)/OVA immunization. (N) MFI of p-S6 in splenic effector-like $CD44^+CD62L^-$ splenic $CD8^+$ T cells after immunization.

In Fig. 5 E–F the PD 1 knock out mice display a brisk effector $CD8^+$ expansion that collapses after day 21, but three points remain unclear: first, why was an ex vivo restimulation step required instead of analysing the cells directly; second, would immunisation with a strongly immunogenic peptide elicit a similarly rapid burst and tumour rejection, thereby distinguishing PD 1 specific effects from generic antigen driven proliferation; and third, what is the phenotype or function of the residual cells at day 21—do they re express exhaustion markers or lose cytolytic capacity? Clarifying these issues would greatly refine the interretation of the T cell dynamics.

We appreciate these insightful questions.

First, since degranulation (as measured by the CD107a assay) is a stimulation-dependent and transient process, ex vivo re-stimulation is necessary to trigger CD107a surface expression. This allows antibody access to detect CD107a during active degranulation. To improve the clarify, we rearranged these data and now in new **Figure 1 and 2.**

Second, since PD-1 primarily targets CD28 signaling, as reported by Hui et al. (Science, 2017; PMID: 28280247), a strong TCR stimulation can mitigate PD-1–mediated inhibition, it does not fully override it. Our OVA/poly(I:C) immunization introduces a highly immunogenic peptide (SIINFEKL), which significantly delays tumor growth.

Third, we performed new experiments to assess the expression of Tox, NKG7, and GZMB in residual $CD8^+$ T cells at day 21 post-immunization. Within the effector memory $CD44^+CD62L^-$ $CD8^+$ T cell compartment, our new findings show that the absence of PD-1 increased the expression of cytotoxic molecules (NKG7, Perforin) and exhaustion-like markers, (TOX and TIM3) compared with control cells after immunization. Although NKG7 and perforin levels did not differ significantly between Pdcd1-deficient splenic $CD8^+$ T cells at day 7 and day 21 immunization, we observed higher expression of exhaustion-associated markers (TOX and TIM3) at day 21 compared with day 7, indicating these cells are developing towards a more terminally differentiated state.

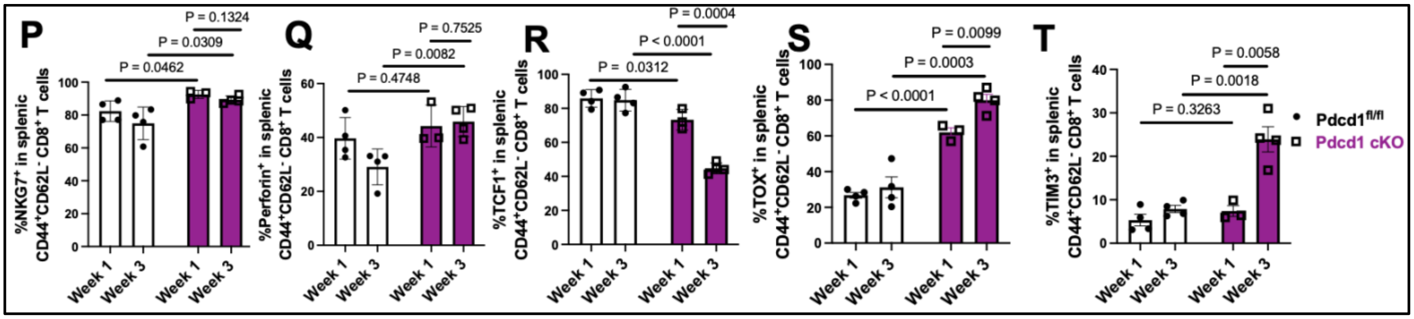

Figure 7. PD-1 deficiency distinctly shapes effector- and memory phase immunization responses in long-term anti-tumor immunity. (P-T) Frequencies of $NKG7^+$ (P), $Perforin^+$ (Q), $TCF1^+$(R), TOX^+(S) and $TIM3^+$ (T) in splenic $CD44^+CD62L^-$ $CD8^+$ T cells at 1-week (n=4 and n=3) and 3-week (n=4 and n=4) poly(I:C)/OVA immunization in $Pdcd1^{fl/fl}$ and Pdcd1 cKO mice.

Fig 5 H-J When exactly does the PD-L1 blockade occur in the experimental timeline? Do the targeted T cells exhibit features of terminal exhaustion, or do they retain the potential for functional rescue? It is well-

established that PD-1/PD-L1 blockade primarily restores cells at a pre-exhausted or stem-like stage, but not those that are terminally exhausted. According to the Materials section (line 355), PD-L1 antibody was injected 7 days post tumor implantation. How does the tumor growth delay compare between fl/fl and fl/fl plus PD-L1 blockade, as well as KO and KO plus PD-L1 blockade groups? Is the observed tumor growth delay truly attributable to PD-L1 inhibition, or could the antibody alter immunological synapse conformation, thereby enhancing CD28/TCR signaling?

We appreciate these questions seeking clarification and insights into the potential mechanisms. We administered PD-L1 antibody on days 7, 10, 13, 16, and 19 following the injection of B16-OVA tumor cells. In our experimental setting, we hypothesize that the observed delay in tumor growth is attributable to the blockade of PD-L1's interaction with CD80, another receptor expressed on activated T cells. CD80 on T cells has been shown to suppress T cell expansion and memory formation (PMID: 20472828; PMID: 23170254). Therefore, PD-L1 antibodies that block PD-L1/CD80 interaction may enhance the expansion of effector and memory T cells, which are essential for durable tumor control. Additionally, PD-L1 blockade may disrupt the cis-interaction between CD80 and PD-L1 on antigen-presenting cells, thereby freeing CD80 to engage CD28. This could enhance CD28-mediated co-stimulation and help prevent T cell exhaustion. However, due to space limitations and the need for additional experiments beyond the scope of this paper, we have removed these data from the revised manuscript.

Finally, outline whether peripheral blockade of PD-1 or PD-L1 using specific antibodies can recapitulate the reported thymic specific PD-1 cKO phenotype.

We greatly appreciate the reviewer's suggestion for performing this experiment. To evaluate whether peripheral PD-1 blockade could recapitulate the thymic effector phenotype observed in our PD-1 cKO mice, we administered intraperitoneal injections of 300 µg anti-PD-1 antibody (clone: G4) every two days for a total of three doses (day 1, day 3, day 5, day 7 and day 9) in naïve mice (**New Extended Data Fig. 10**). While peripheral PD-1 blockade clearly induced an effector phenotype in splenic $CD8^+$ T cells—characterized by increased expression of NKG7, perforin, granzyme B, and TOX, and decreased TCF-1 expression—we did not observe any corresponding phenotypic changes in thymic SP $CD8^+$ T cells, regardless of anti–PD-1 treatment. These data suggest acquisition of cytotoxic effector features we observed in Pdcd1-deficient T cells may be due to cell-intrinsic PD-1 signals and cannot be recapitulated by peripheral PD-1 blockade alone, which could be indirectly supported by a recent study from Dr. Sharpe's group (PMID: 40705010).

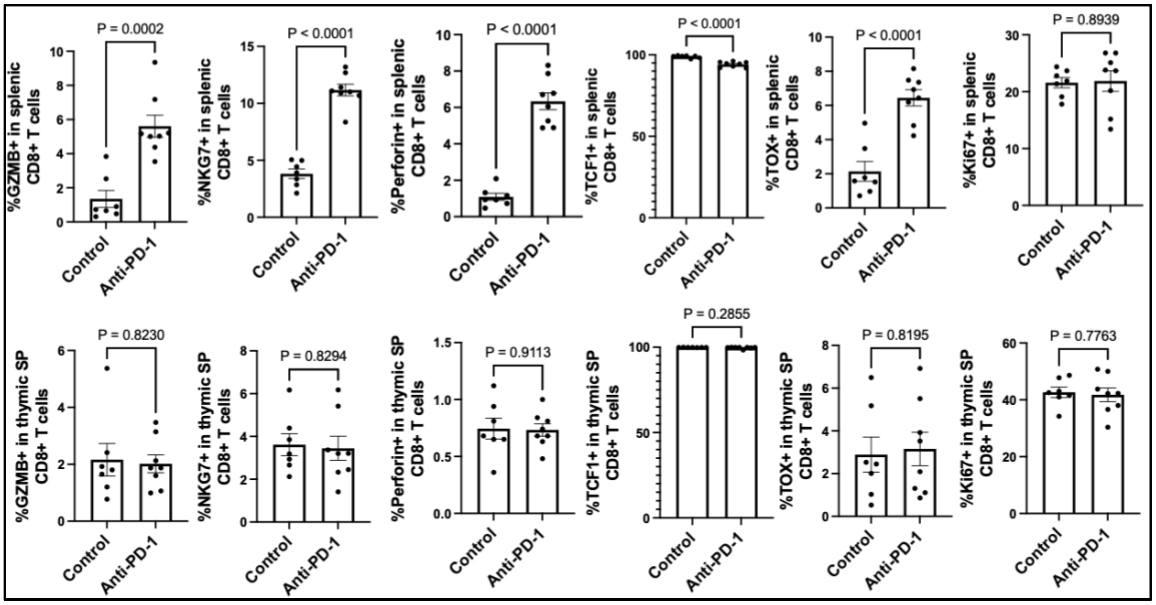

Extended Data Fig 10. Peripheral anti-PD-1 blockade does not recapitulate thymic effector phenotype in Pdcd1 cKO mice. Frequencies of GZMB$^+$, NKG7$^+$, Perforin$^+$, TCF1$^+$, TOX$^+$ and Ki67$^+$ in splenic CD8+ T cells (**A-F**) and thymic SP CD8$^+$ T cells (**G-L**) with or without 5 doses of 300 µg anti-PD-1 blockade every other day (day 1, 3, 5, 7, 9). **(M)** Schematic role of PD-1 in regulation of cytotoxic effector capacity of CD8$^+$ T cells in thymus and peripheral tissues including tumor tissues.

The discussion must be embedded in a larger context; it appears to be mainly descriptive of the results.
We appreciate this very encouraging and inspiring suggestions. We re-wrote our discussion session in the revised manuscript.

Minor Concern
Fig. 4 could be moved to an Extended Data Fig.
Thank you for the suggestion and we removed part of Figure 4 into Extended Data Fig 7. Since we added new data using PD-1-NKG7 double conditional knockout mice that is also suggested by reviewer 3, we rearranged these data in new Figure 5.

Reporting numbers of samples (n) is necessary throughout to give the reader an idea of the statistics.
We have added the number of samples in each figure legend. We appreciate the advice.

Show the p-values for the correction of multiple comparisons as supplementary information. This is not clearly visible.

We have added the p-values for multiple comparisons now in new Figure 7 and Extended Data Figure 9.

Furthermore, is a sequencing depth of approximately 40,000 reads per cell sufficient to generate reliable and statistically robust results? Could low level transcription inflate cluster assignment.

We appreciate this critical question about the rigor of scRNA-seq analysis. While 40,000 reads/cell is generally considered moderate depth and adequate for standard clustering and marker identification, but it is not for exhaustive detection of rare transcripts. We agree that a low-level transcription can inflate cluster assignment, but we performed critical QC and normalization to filter low-quality cells and reduce technical noise.

Reviewer #3 (Remarks to the Author):

Majors:
1 The scRNA-seq data (Figure 1B and 1C) indicate that all highlighted genes, except Nkg7, are enriched in CD8-Pdcd1 cKO SP CD8$^+$ T cells versus control SP CD8$^+$ T cells, while Cluster 1 is markedly less represented than Cluster 0. The authors' emphasis on cytotoxicity and exhaustion, with little consideration of stem-like characteristics, seems incomplete. A similar oversight is evident in their analysis of splenic SP CD8$^+$ T cells.

We appreciate the reviewer's helpful suggestion. In our thymus single cell RNA sequencing data, we identified two additional clusters of CD8$^+$ T cells with stem-like or naïve-like transcriptional features. This observation indicates that intrinsic PD-1 ablation after thymic positive selection does not uniformly drive all thymic SP CD8$^+$ T cells towards an effector fate. Instead, only a subset acquires cytotoxic effector characteristics in the absence of PD-1. Although these two clusters express naïve or stem like-associated markers, they also express TCR-signaling–related genes such as *Lck* and *Nfatc1*, suggesting a degree of tonic or low-level signaling. We did not assign specific functional labels to these clusters because several important questions remain unsolved. For instance, given that single cell RNA sequencing captures cells at only a single time point, it is unclear whether these naïve or stem-like cells are inherently incapable of acquiring cytotoxic potential in the absence of PD-1, or whether they simply have not yet transitioned towards an effector state. Moreover, it remains to be

determined whether post-positive selection TCR signaling is sufficient for thymic CD8+ T cells to acquire effector potential, and what factors dictate certain thymic CD8+ T cell subsets undergo latent effector differentiation in the absence of PD-1. Addressing these questions in future studies will be essential for a full understanding of the phenotype and developmental trajectory of PD-1 deficient thymic CD8+ T cells that retain naïve or stem-like characteristics. Similar uncertainties exist in the periphery, where it can be difficult to determine whether naïve or stem-like CD8+ T cells in the absence of PD-1 actually reflect a quiescent naïve or stem-like state or represent "primed" cells in their earlier stages of activation or differentiation.

2 The authors state that isolated thymic SP CD8+ T cells were stimulated with PMA and ionomycin. However, it is unclear why the control Pdcd1fl/fl SP CD8+ T cells fail to exhibit activation markers such as CD107a, NKG7, and GZMB (Figure 1N–R). The validity of the stimulation conditions or the responsiveness of the control cells are needed.

We appreciate the reviewer's critical comments. In the original Figure 1N-R, only thymic SP CD8+ T cells were stimulated with PMA and ionomycin as shown in Figure 1N-O, since CD107a exposure is a stimulation-dependent and transient event that requires *ex vivo* activation to be reliably detected. The samples shown in original Figure 1P-R were stained directly with anti-CD107a antibody following PMA and ionomycin stimulation. We thank the reviewer for highlighting this out, and we have now reorganized these panels accordingly for clarify in **new Figure 1 and 2** for clarity. To ensure the validity of our stimulation conditions, we confirmed a robust CD107a degranulation of CD8+ T cells in the spleen and tumor in PD-1 cKO mice (**new Figure 3D and 4O**), while the control groups exhibited ~13% CD107a+ cells as expected in these studies (new Fig. 3D, 4O). The relatively less CD107a degranulation observed in the control thymic CD8+ T cells likely reflect an intrinsic biological difference. To that end, thymic SP CD8+ T cells in control (Pdcd1$^{fl/fl}$) mice may have limited pre-existing cytolytic machinery and reduced granule content, which constrains the magnitude of CD107a degranulation even under maximal PMA/ionomycin stimulation.

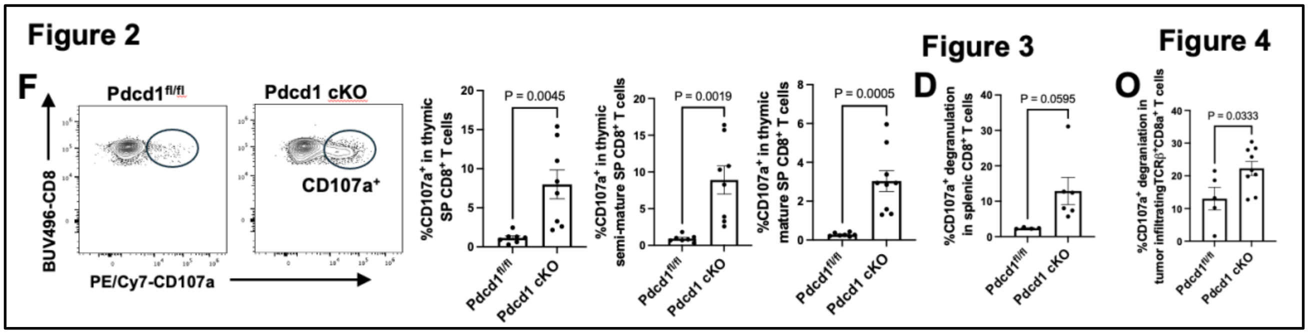

Figure 2. Thymic SP CD8+ T cells exhibited cytotoxic effector function in the absence of PD-1. (F) Gating strategy (left) and percentage of CD107a+ degranulation in thymic SP, semi-mature SP and mature SP CD8+ T cells (right) following PMA and ionomycin stimulation for 4 hours in Pdcd1$^{fl/fl}$ (n=7) and Pdcd1 cKO (n=8) mice.
Figure 3. Increased cytotoxic potential of peripheral effector CD8+ cells in the absence of PD-1. (D). Percentage of CD107a+ production of splenic CD8+ T cells following PMA and ionomycin stimulation for 4 hours in Pdcd1$^{fl/fl}$ (n=4) and Pdcd1 cKO (n=6) mice.
Figure 4. Tumor immunity is regulated by PD-1 to the immunogenicity. Percentage of CD107a+ degranulation following PMA and ionomycin stimulation for 4 hours in Pdcd1$^{fl/fl}$ (n=5) and Pdcd1 cKO (n=9) mice.

3 The authors propose that NKG7+ Pdcd1 cKO CD8+ T cells are the main contributors to tumor control. Have the authors considered performing functional validation, such as selective depletion or adoptive transfer of these NKG7+ subsets, to confirm their direct anti-tumor activity?

We greatly appreciate the reviewer's insightful comments on this point. To address whether NKG7 plays a necessary role in establishing durable anti-tumor immunity in the absence of PD-1, we generated a new strain of

Pdcd1$^{fl/fl}$Nkg7^{flfl} CD8 E8i Cre mice, in which both PD-1 and NKG7 are conditionally deleted after thymic positive selection in thymic CD8$^+$ T cells. Successful deletion NKG7 is shown in **Extended Data Fig. 7D**. Using this model, we subcutaneously injected B16-OVA tumors in CD8-Pdcd1 cKO mice and Pdcd1$^{fl/fl}$ control mice (**Fig. 5I-J**). We found mice lacking both PD-1 and NKG7 deficient CD8$^+$ T cells exhibited increased tumor growth compared with mice with only PD-1 deficient CD8$^+$ T cells; nevertheless, they still showed partial tumor control compared with Pdcd1$^{fl/fl}$ mice. These results indicate that NKG7 is required for durable anti-tumor immunity in the absence of PD-1, although a loss of NKG7 did not completely abrogate anti-tumor immunity in the absence of PD-1 in CD8$^+$ T cells.

I **J**

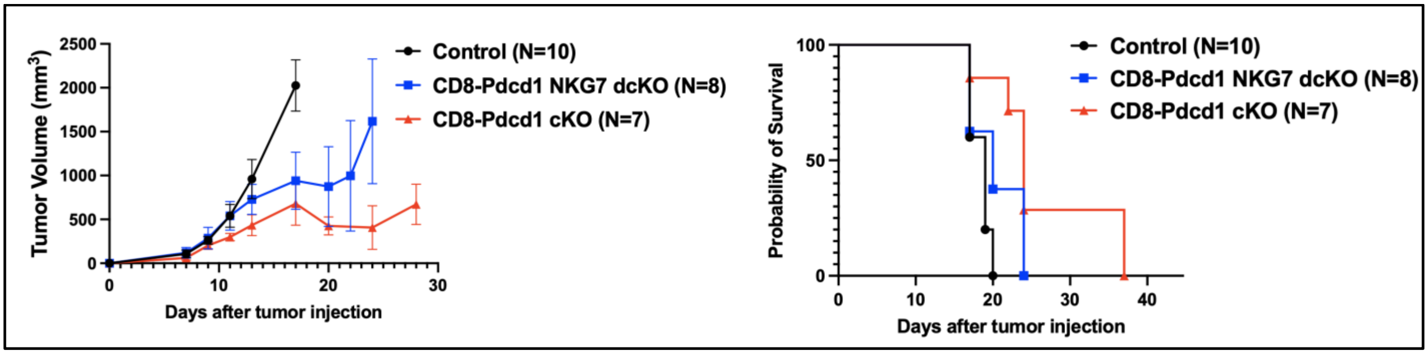

Figure 5. NKG7 is necessary to regulate anti-tumor immunity in the absence of PD-1. (I-J) Functional validation of NKG7 in regulation of PD-1 deficient CD8$^+$ T cell-mediated anti-tumor immunity.
(I) Tumor growth curve of control (n=10), CD8-Pdcd1 cKO (n=8) and *Pdcd1$^{fl/fl}$ Nkg7*$^{fl/fl}$CD8 E8I cre$^+$ (CD8-Pdcd1 Nkg7 dcKO, n=7) mice following B16-OVA tumor injection.
(J) Survival curves of control, CD8-Pdcd1 cKO and CD8-Pdcd1 Nkg7 dcKO mice after B16-OVA tumor injection.

Extended Data Figure 7(D) The frequency of NKG7$^+$ in splenic CD8+ T cells of CD8-Pdcd1 Nkg7 dcKO (n=8) compared with CD8-Pdcd1 cKO (n=6) and control mice (n=8) at the end point post B16-OVA tumor challenge.

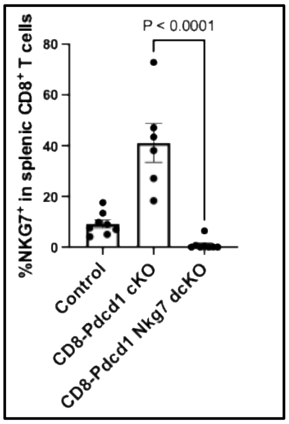

4 The author claims that PD-L1 blockade releases CD80 from its interaction with PD-L1, thereby permitting CD80 to engage CD28, which in turn promotes effector T cell expansion via co-stimulatory signaling and mitigates exhaustion. To substantiate this mechanistic claim, direct experimental evidence should be provided.

We appreciate this insightful comment. PD-L1 blockade may disrupt the cis-interaction between CD80 and PD-L1 on antigen-presenting cells, thereby freeing CD80 to engage CD28. This interaction could enhance CD28-mediated co-stimulation and help prevent T cell exhaustion. However, due to space limitations and the need for additional experiments beyond the scope of this paper, we have removed these data from the revised manuscript.

Minors:
1 Based on the UMAP in Extended Data Fig. 7B, human NKG7$^+$CD8$^+$ and NKG7$^-$CD8$^+$ T cells did not exhibit significant transcriptional differences, either within individual patients or between different patients.

We relabeled the data in Extended Figure 7 to improve the clarity.

2 It may be interesting to integrate scRNA-seq data of CD8$^+$ T cells from the thymus, spleen, and tumor-infiltrating lymphocytes (TILs).

We appreciate the reviewer's creative and thoughtful suggestion. We explored the possibility of integrating thymus, spleen, and TIL scRNA seq datasets for a joint analysis. However, since each dataset was generated with distinct filtering strategies, quality-control thresholds, and batch-specific preprocessing pipelines, their inherent incompatibilities prevented a robust integration without introducing significant technical artifacts. For these reasons, a combined analysis across the three tissues was not feasible.

3 The authors suggest that PD-1 KO CD8$^+$ T cells can exhibit the dual role of cytotoxic and exhaustion functions against tumors, depending on the tumor context. However, why do Pdcd1 cKO CD8$^+$ T cells effectively control tumors only when tumor immunogenicity is high or intermediate? Does the underlying mechanism among the three tumor types appear to be the same? As the authors mention, immunized CD8-Pdcd1 cKO cells can develop long-term memory rather than exhaustion phenotypes. Why, then, does high immunogenicity drive the transition of effector CD8$^+$ T cells toward a memory phenotype rather than exhaustion? These need to be at least discussed.

We appreciate these insightful questions. The possible reason for effective tumor control with high immunogenicity is that tumor immunogenicity may shape the fate and function of PD-1-deficient CD8$^+$ T cells. High immunogenic tumors provide strong TCR stimulation that drives rapid clonal expansion and efficient tumor clearance. Under these circumstances, the effector response is robust but transient, thereby limiting prolonged antigen exposure that would otherwise promote terminally exhaustion. In contrast, tumors with low immunogenicity generate weaker TCR signals that may trigger a more diverse T cell repertoire for targeting the tumor cells and effective tumor killing. To explore this, we added new TCR sequencing analysis comparing thymus and tumor CD8$^+$ T cells after B16-OVA (higher immunogenicity) or B16-F10 (lower immunogenicity) challenge. Interestingly, we observed greater TCR clonotype overlap between thymus and tumor in the low-immunogenic B16-F10 model than in the B16-OVA model in the absence of PD-1 (**New Fig. 6A-C**). This suggests that when antigenic strength is low, a broader set of TCR repertoire—including RTE-derived clones—may be recruited to recognize weak tumor antigens as a potential compensation mechanism of tumor immunity. However, since each clonotype of TCRs receives relatively weak but persistent stimulation, these sub-optimal T cell responses are less efficient at mediating tumor control even in the absence of PD-1. In our immunization model, our new data demonstrates some upregulated exhaustion-associated markers, including TOX and TIM-3, in effector memory CD44$^+$CD62L$^-$ CD8$^+$ T cells on day 12 compared to day 7 post immunization in the absence of PD-1 (**New Fig. 7P-T**). Collectively, our findings suggest a prolonged antigen exposure may drive CD8$^+$ T cells towards exhaustion, whereas strong and transient antigen stimulation facilitates long-term anti-tumor immunity in the absence of PD-1.

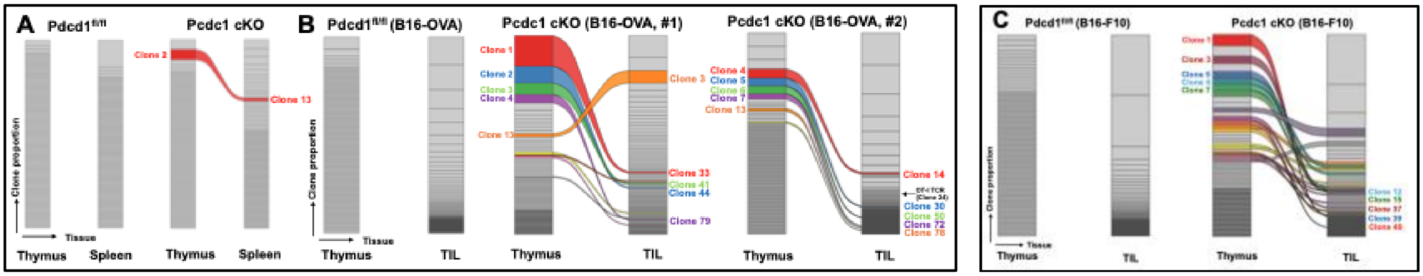

Figure 6. Shared CD8$^+$ T cell clonotypes in the thymus and tumors in the absence of PD-1. A–C) TCR sequencing analysis comparing CD8$^+$ T cell clonotypes across thymus, spleen, and tumors in Pdcd1$^{fl/fl}$ and

Pdcd1 cKO mice. (**A**) Comparison of CD8$^+$ T cell clonotypes between thymus and spleen of Pdcd1$^{fl/fl}$ (left) and Pdcd1 cKO mice (right) at baseline. (**B**) Comparison of CD8$^+$ T cell clonotypes between thymus and CD8$^+$ TILs of Pdcd1$^{fl/fl}$ (left), two representative Pdcd1 cKO mice (middle, right) on day 12 after B16-OVA tumor challenge. Arrow indicates TCR clone that is identical to OT-1 TCR. (**C**) Comparison of CD8$^+$ T cell clonotypes between thymus and CD8$^+$ TILs of Pdcd1$^{fl/fl}$ (left) and Pdcd1 cKO mice (right) on day 12 post B16-F10 tumor challenge.

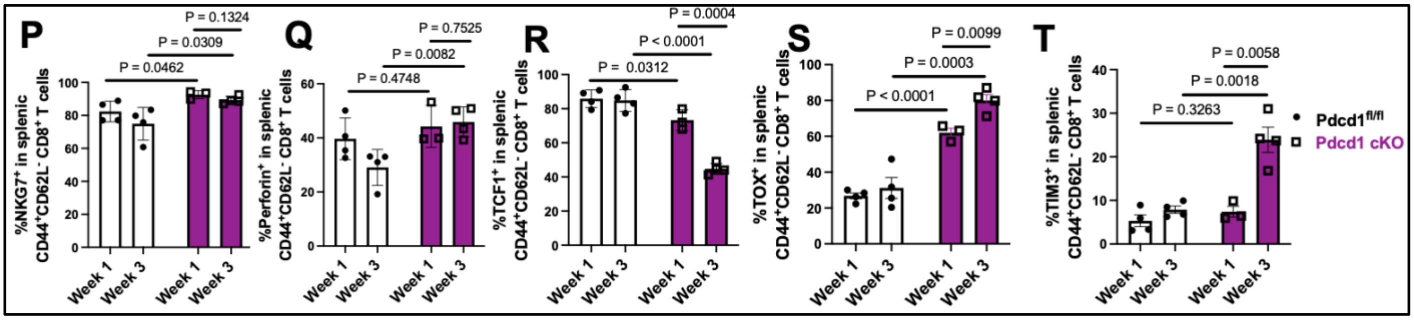

Figure 7. PD-1 deficiency distinctly shapes effector- and memory phase immunization responses in long-term anti-tumor immunity. (P-T) Frequencies of NKG7$^+$ (P), Perforin$^+$ (Q), TCF1$^+$(R), TOX$^+$(S) and TIM3$^+$ (T) in splenic CD44$^+$CD62L$^-$ CD8$^+$ T cells at 1-week (n=4 and n=3) and 3-week (n=4 and n=4) poly(I:C)/OVA immunization in Pdcd1$^{fl/fl}$ and Pdcd1 cKO mice.

4 Both mouse data and HCC patient data indicate that Pdcd1 cKO CD8$^+$ T cells expressing NKG7 contribute to anti-tumor functions. Can NKG7- CD8+ TILs be used as ICB treatment biomarker for tumor patients? Discussion on this point is suggested.

We appreciate reviewer's suggestion in highlighting the importance of NKG7 as a marker for ICB treatment. Yes, we believe patients with low or no NKG7 expression in CD8$^+$ T cells would be able to predict a non-responsiveness to ICB treatment as reported by a recent review article (PMID 40824199). We have added this point in the discussion.

Reviewer #4 (Remarks to the Author):

Majors:

1 The scRNA-seq data (Figure 1B and 1C) indicate that all highlighted genes, except Nkg7, are enriched in CD8-Pdcd1 cKO SP CD8$^+$ T cells versus control SP CD8$^+$ T cells, while Cluster 1 is markedly less represented than Cluster 0. The authors' emphasis on cytotoxicity and exhaustion, with little consideration of stem-like characteristics, seems incomplete. A similar oversight is evident in their analysis of splenic SP CD8$^+$ T cells.

2 The authors state that isolated thymic SP CD8$^+$ T cells were stimulated with PMA and ionomycin. However, it is unclear why the control Pdcd1fl/fl SP CD8$^+$ T cells fail to exhibit activation markers such as CD107a, NKG7, and GZMB (Figure 1N–R). This raises questions about the validity of the stimulation conditions or the responsiveness of the control cells.

3 The authors suggest that PD-1 KO CD8$^+$ T cells can exhibit the dual role of cytotoxic and exhaustion functions against tumors, depending on the tumor context. However, why do Pdcd1 cKO CD8$^+$ T cells effectively control tumors only when tumor immunogenicity is high or intermediate? Does the underlying mechanism among the three tumor types appear to be the same? As the authors mention, immunized CD8-Pdcd1 cKO cells can develop long-term memory rather than exhaustion phenotypes. Why, then, does high immunogenicity drive the transition of effector CD8$^+$ T cells toward a memory phenotype rather than exhaustion?

4 The authors propose that NKG7$^+$ Pdcd1 cKO CD8$^+$ T cells are the main contributors to tumor control. Have the authors considered performing functional validation, such as selective depletion or adoptive transfer of these

NKG7^{+} subsets, to confirm their direct anti-tumor activity?
5. Both mouse data and HCC patient data indicate that Pdcd1 cKO CD8^{+} T cells expressing NKG7 contribute to anti-tumor functions. Why is it that NKG7^{-} CD8^{+} TILs are enriched in the PD-1/PD-L1 pathway, whereas NKG7^{+} CD8^{+} TILs are not enriched in the "PD-1/PD-L1 cancer immunotherapy" pathway? Can NKG7- CD8+ TILs be used as ICB treatment biomarker for tumor patients?
6 The author claims that PD-L1 blockade releases CD80 from its interaction with PD-L1, thereby permitting CD80 to engage CD28, which in turn promotes effector T cell expansion via co-stimulatory signaling and mitigates exhaustion. To substantiate this mechanistic claim, direct experimental evidence should be provided.

Minors:
1 Based on the UMAP in Extended Data Fig. 7B, human NKG7^{+}CD8^{+} and NKG7^{-}CD8^{+} T cells did not exhibit significant transcriptional differences, either within individual patients or between different patients.
2 It may be interesting to integrate scRNA-seq data of CD8^{+} T cells from the thymus, spleen, and tumor-infiltrating lymphocytes (TILs).

Since these comments of reviewer #4 are identical with comments raised by reviewer #3, please refer to our answers to reviewer #3 for our responses. Thank you so much!

Reviewer #2 (Remarks to the Author):

Summary
In this revised submission of the manuscript by Mao et al., the authors have clearly made a substantial effort to address prior reviewer concerns, including adding an ex vivo thymocyte-mediated killing assay, expanding the thymus–tumor TCR sequencing data set with additional mice, and incorporating RAG-GFP reporter experiments to track recent thymic emigrants (RTEs) in tumors with and without anti–PD-1 therapy. They also reframe the manuscript more explicitly around thymic programming ("latent effector differentiation") and reduce overstatement in places. The manuscript proposes a thymus-intrinsic checkpoint function for PD-1 in shaping the CD8 single-positive compartment, suggesting that loss of PD-1 permits emergence of an effector-like/cytotoxic program already in the thymus and that these cells contribute to tumor control. The most compelling novelty remains the thymic angle (resident CD8 SP with effector features) together with the observation that genetic Pdcd1 deletion is not trivially phenocopied by therapeutic anti–PD-1 blockade. However, the main weaknesses remain that the functional cytotoxicity claim is still not supported by statistically robust killing data, and that the thymus–TIL TCR overlap is still presented largely descriptively without a rigorous null model controlling for sampling depth and clonality. The peripheral phenotyping is extensive but in many places confirmatory of well-described PD-1–loss phenotypes and could be substantially condensed.

Major points
1. Scope and focus of peripheral assays. The thymus-centered concept is strong: the manuscript links PD-1 loss to an effector-like CD8 SP program in the thymus, makes a reasonable effort to address recirculation, and connects the program to tumor control with functional genetics and a thymus–tumor bridge. In contrast, much of the peripheral spleen/blood baseline phenotyping (multiple redundant effector/exhaustion marker panels in peripheral CD44+CD62L− cells, repeated activation/proxy readouts, and additional spleen scRNA feature/dot plots) remains largely confirmatory of peripheral PD-1–loss effector skewing. For a Nature Communications-level research manuscript, I recommend condensing these readouts and moving most redundant peripheral marker surveys to the Supplement unless they directly a) exclude alternative explanations, b) connect thymus output to tumors, or c) test causality.

We appreciate this reviewer for the insightful comments and constructive suggestions. Accordingly, we moved our original **Figure 3** into **Extended Data Figure 4 and 6**.

2. Newly added thymocyte-mediated killing assay does not robustly support active cytotoxicity
I appreciate that the authors added an ex vivo thymocyte-mediated cytotoxicity assay in response to earlier concerns. However, as presented, the killing assay is not statistically convincing: only one E:T condition shows a borderline effect ($p = 0.0503$), while the other dilutions are clearly non-significant, arguing against a consistent dose–response. Because multiple E:T ratios are tested in parallel, these comparisons form a multiple-testing family and the borderline result would not survive simple correction; at most, this should be labeled exploratory/trend rather than evidence of bona fide/active cytotoxicity. A more appropriate analysis would treat the dilution series globally (two-way ANOVA or mixed-effects model with genotype × E:T interaction), or use a curve-based summary metric (AUC/EC50), and only proceed to corrected post hoc comparisons if the global effect is significant.

We thank the reviewer for the careful evaluation of the thymocyte-mediated cytotoxicity assay and for the constructive statistical insights. In response, we increased biological repeats (added more mice to each group) and reanalyzed the full dilution series using a mixed-effects model including genotype, E:T ratio and their interaction term. The global analysis revealed a significant effect of E:T ratio ($P = 0.0074$), a significant main effect of genotype ($P = 0.0011$) and a significant genotype × E:T ratio interaction ($P = 0.0348$). These results indicate that PD-1 deficiency significantly

increased the killing activity of CD8+ thymocytes across the dilution series. Post hoc comparisons, corrected for multiple testing, confirmed a statistically significant decrease in the live target cells (shown as percentage of tumor-only control) at the 1:20 E:T condition. Rather than claiming the definitive cytotoxic competence, we now changed our interpretation as PD-1–deficient thymocytes exhibited a genotype-dependent trend toward increased target cell killing *ex vivo*, detectable under higher effector density conditions. The revised data and statistical analyses are presented in the updated main **Figure 2E** and Methods section.

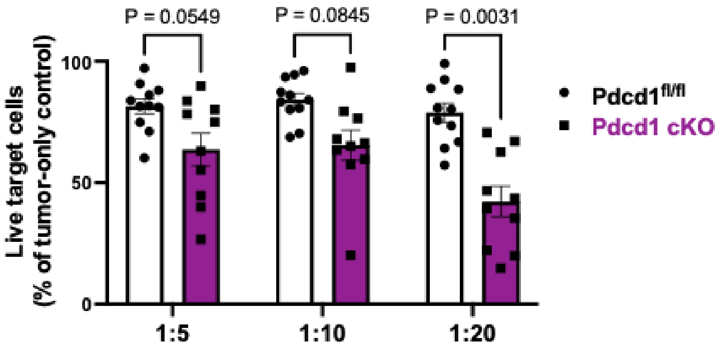

Figure 2. Thymic SP CD8+ T cells exhibited cytotoxic effector function in the absence of PD-1. (**E**) Number of live target P815 mastocytoma cells (Calculated as % of tumor-only control) after 48-hour co-culture with isolated thymic CD8+ T cells from Pdcd1fl/fl (n=11) and Pdcd1 cKO (n=10) at the target-to-effector ratios of 1:5, 1:10 and 1:20. Each point represents one mouse, with three technical replicates averaged per mouse. Statistical analyses in (**E**) were performed using mixed-effects model with Geisser-Greenhouse correction to account for repeated measures across dilution series, followed by Sidak’s multiple comparison test.

3. Expanded thymus–TIL TCR overlap still requires depth- and clonality-aware statistics

I appreciate that the authors expanded the TCR sequencing to additional mice/tumor settings and added a revised wording (“latent effector differentiation … can contribute to the TCR repertoire of TILs”). Nevertheless, the thymus–TIL overlap remains a central dataset supporting the proposed thymus-to-tumor link, and it is still not evaluated against an appropriate expectation. Even when all samples are processed identically and the same top-N strategy is used, observed overlap can be strongly influenced by effective sequencing depth, numbers of unique clonotypes, and clonotype frequency distributions (oligo- vs polyclonality), which is particularly consequential with few mice.

At minimum, the authors should:

• rarefy/downsample thymus and TIL repertoires to matched depth within each mouse
• report overlap using depth-robust metrics (e.g., Jaccard plus a frequency-weighted metric such as Morisita–Horn)
• benchmark observed overlap against a within-mouse permutation/bootstrapping null model that preserves marginal clonotype frequencies
• present mouse-level statistics and, if possible, demonstrate consistency across mice

This would clarify whether overlap reflects enrichment beyond sampling/detection and overall clonality.

We thank the reviewer for this insightful suggestion regarding the statistical rigor of our TCR overlap analysis. We agree that simple "shared clone" counts can be influenced by sequencing depth and clonal dominance. In response, we reanalyzed our TCR sequencing data to include depth-normalized rarefaction and robust similarity metrics across all experimental cohorts. We first employed a stochastic downsampling (rarefaction) approach to match the sequencing depth within each mouse comparison. For every pair, we identified the sample with the lowest total read count and randomly subsampled the larger repertoire to match this depth. The mouse-level statistics are presented in **Supplementary Table 4**.

Supplementary Table 4. Rarefication number per mouse for TCR sequencing

Comparison	Organ	Mouse condition	Productive clonotype Reads number	**After Rarefication**
Comparision 1. Baseline thymus-spleen	Thymus	Control Baseline	1,909	**1,909**
	Spleen	Control Baseline	2,943	
	Thymus	Pdcd1 cKO Baseline	1,097	**1,097**
	Spleen	Pdcd1 cKO Baseline	1,252	
Comparision 2. B16-OVA thymus-tumor	Thymus	Control B16-OVA	4,125	**3,623**
	Tumor	Control B16-OVA	3,623	
	Thymus	Pdcd1 cKO B16-OVA	5,557	**5,557**
	Tumor	Pdcd1 cKO B16-OVA	7,558	
Comparision 3. B16-F10 thymus-tumor	Thymus	Control B16-F10	5,901	**2,834**
	Tumor	Control B16-F10	2,834	
	Thymus	Pdcd1 cKO B16-F10	7,222	**5,759**
	Tumor	Pdcd1 cKO B16-F10	5,759	
B16-OVA thymus-tumor mouse 2	Thymus	Pdcd1 cKO B16-OVA	2,139	**1,765**
	Tumor	Pdcd1 cKO B16-OVA	1,765	

Rarefaction was repeated for 1,000 independent iterations to ensure stability. To evaluate the overlap, we moved beyond the "Top N" strategy and now report two distinct, depth-robust metrics:

1. **Jaccard Index:** To measure the similarity of unique clonotype identities (binary presence/absence).
2. **Morisita-Horn Index:** To provide a frequency-weighted overlap measurement that is highly robust to variations in sampling depth and library size.

To clarify whether the observed overlap reflects a biological link or random sampling noise, we benchmarked our within-mouse results against a cross-mouse null model for comparison (e.g., Mouse A TIL vs Mouse B Thymus). We performed our analysis at baseline (thymus vs spleen) as well as two independent tumor models (B16-OVA and B16-F10 tumors) between thymus and TIL. At baseline, we observed a systemic increase in the overlapping productive pair of TCRs between thymus and spleen in the absence of PD1 compared with controls with the increase of both Jaccard and Morisita-Horn index. In mice with B16-OVA tumors, we also observed a significant increase in the overlapping TCR clonotypes between thymus and tumor in the absence of PD-1 compared with controls with concordant increases in both identity-based and frequency-weighted metrics. In mice with B16-F10 tumors, we similarly observed a significant increase in the overlapping TCR clonotypes between thymus and tumor in the absence of PD-1 compared with controls. When comparing with cross-mouse null model, we all observed a significant elevation in the absence of PD-1 both at baseline and following tumor injection, indicating that the overlap is mouse-specific. Collectively, these data confirms that increased overlap between peripheral TCR clonotypes between thymic TCR clonotypes, reinforcing our conclusion that the "thymus-to-tumor" link is a direct consequence of PD-1 deficiency and is not driven by sampling or detection artifacts. We have updated **Figure 5**, **Extended Data Fig 9.** and the Methods section to reflect these new analyses.

4. Genetic deletion versus therapeutic blockade: distinguish programming from acute absence of PD-1 signaling
I appreciate that the revised manuscript includes additional data comparing peripheral anti–PD-1 blockade to the genetic setting (including RAG-GFP/RTE analyses and peripheral blockade experiments). The observation that therapeutic anti–PD-1 does not phenocopy genetic Pdcd1 deletion remains notable and consistent with an early programming/imprinting effect, but it does not by itself establish developmental imprinting. Because Pdcd1 loss removes an inhibitory brake at the time of stimulation, several functional readouts could be explained by acute absence of PD-1 signaling rather than stable developmental reprogramming. With minimal additional work, the authors should show a direct side-by-side in vitro comparison under identical conditions in which WT cells are assessed with acute

PD-1/PD-L1 blockade (matched stimulation and E:T) directly against Pdcd1 cKO cells, ideally using rigorously naïve CD8 T cells from young, tumor-free, age-matched mice to avoid confounding by prior activation/homeostatic expansion. Demonstrating that the cKO phenotype exceeds what acute blockade produces would substantially strengthen the imprinting interpretation without requiring additional tumor models. If blockade effects are central to conclusions, pharmacodynamic target engagement (at least one clear regimen/engagement control) should be documented.

We thank the reviewer for the insightful and constructive comments. We fully agree that distinguishing developmental programming from the acute absence of PD-1 signaling is critical for interpreting the phenotype observed in Pdcd1 cKO $CD8^+$ T cells. To directly test whether acute PD-1 blockade is sufficient to recapitulate the effector differentiation phenotype observed in Pdcd1 cKO cells, we performed a direct, side-by-side *in vitro* comparison under identical conditions using naïve $CD8^+$ T cells from age-matched, tumor-free young mice (6-week-old). $Pdcd1^{fl/fl}$ and Pdcd1 cKO naïve $CD8^+$ T cells were stimulated with anti-CD3 and anti-CD28 antibodies for 24 hours. To model acute PD-1 blockade, these $Pdcd1^{fl/fl}$ naive $CD8^+$ T cells were additionally cultured in the presence of anti–PD1 antibody at a dose previously validated in our studies to achieve complete PD-1/PD-L1 blockade (PMID: 23170254). As a pharmacodynamic control, anti–PD-1 antibody was also included in cultures of Pdcd1 cKO cells. Flow cytometric analysis confirmed robust binding of anti-PD-1 antibody to $Pdcd1^{fl/fl}$ $CD8^+$ T cells, with minimal binding observed in Pdcd1 cKO cells, consistent with loss of PD-1 expression.

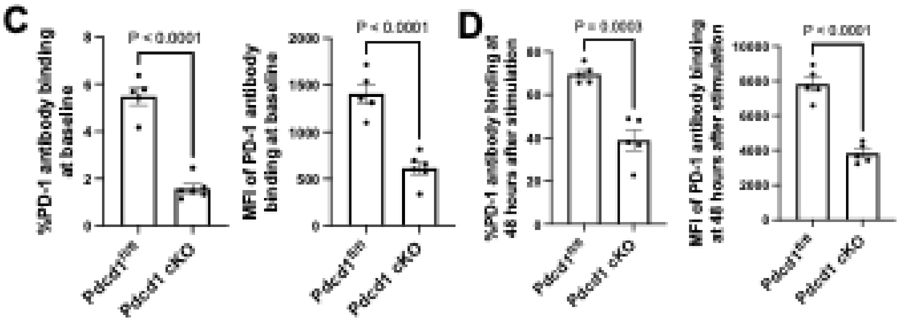

Extended Data Figure 5. (**C-D**) Pharmacodynamic PD-1 binding target engagement assay. Splenic $CD8^+$ T cells from $Pdcd1^{fl/fl}$ (n=5) and Pdcd1 cKO (n=5) were incubated with Hamster anti-PD-1 antibody (Clone: G4) at baseline (**C**) or following 48h anti-CD3/CD28 stimulation (**D**), followed by staining with Alexa Fluor 555-conjugated anti-Hamster secondary antibody. Frequencies (Left) and MFI (right) of $AF555^+$ cells indicating PD-1 antibody binding are shown at baseline (**C**) and after stimulation (**D**).

As shown in newly added data, early T cell activation as indicated by CD69 and phosphorylation of S6 (as a readout of mTOR activation) were similar between $Pdcd1^{fl/fl}$ and Pdcd1 cKO $CD8^+$ T cells in the presence of anti-PD-1 antibody. Thus, neither genetic PD-1 loss nor acute PD-1 blockade substantially altered proximal activation signaling under these conditions. In terms of effector differentiation outcomes, we observed an increased frequency of $NKG7^+$ and TOX^+ $CD8^+$ T cells in the genetic absence of PD-1. Acute anti-PD-1 blockade of $Pdcd1^{fl/fl}$ $CD8^+$ T cells, however, did not increase the frequency of $NKG7^+$ or TOX^+ cells compared without blockade. While Pdcd1 cKO cells exhibited higher baseline frequencies of $NKG7^+$ and TOX^+ cells, acute PD-1 blockade did not significantly augment these populations. These findings indicate that enhanced effector differentiation observed in Pdcd1 cKO cells cannot be explained solely by the acute removal of PD-1 inhibitory signaling at the time of stimulation. Together, these results support the interpretation that PD-1 deficiency establishes an effector-like program that is imprinted prior to peripheral activation and persists into the mature $CD8^+$ T cell compartment. In contrast, acute PD-1 blockade in naïve WT $CD8^+$ T cells, even under identical stimulation conditions, is insufficient to induce a comparable effector phenotype. This side-by-side comparison therefore strengthens our conclusion that genetic Pdcd1 deletion results in developmental or early-life programming of effector differentiation rather than a purely acute signaling effect. While our data strongly

support a model in which PD-1 constrains early effector imprinting, future studies defining the precise temporal window, molecular mechanisms, and functional consequences of this programming will be important.

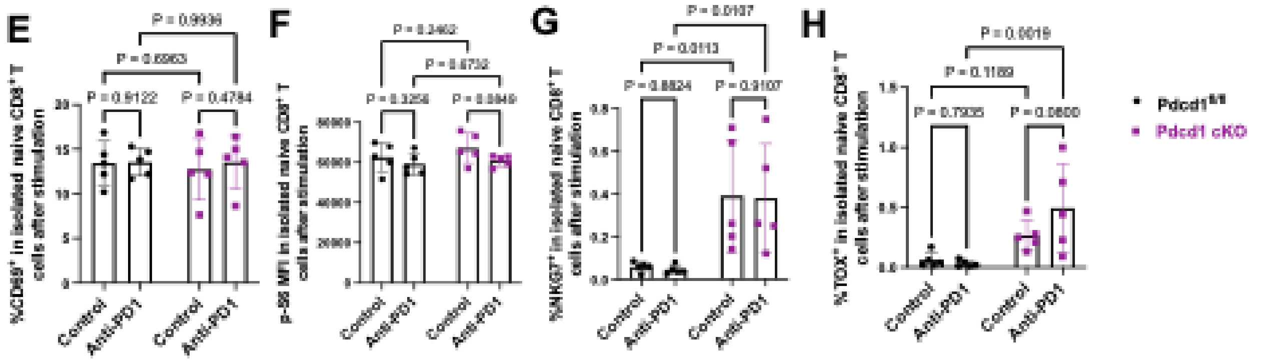

Extended Data Figure 5. (**E-H**) Evaluation of activation and cytotoxic effector protein expression following acute PD-1 blockade. Splenic naïve CD8$^+$ T cells from Pdcd1$^{fl/fl}$ (n=5) and Pdcd1 cKO (n=5) mice were isolated and stimulated with anti-CD3/CD28 stimulation for 48 hours, with or without addition of 20 µg/mL anti-PD-1 antibody. Frequencies of CD69$^+$ (**E**) NKG7$^+$(**G**), TOX$^+$ (**H**), and MFI of p-S6 (**F**) are shown for stimulated naïve CD8$^+$ T cells. Each dot represents one biologically independent mouse, with two technical repeats averaged per mouse prior to statistical analysis. Data are presented as mean ± SEM. Statistical analysis was performed using repeated measures two-way ANOVA followed by uncorrected Fisher's LSD with a single pooled variance.

5. Statistical framework and unit of replication (important for confidence across the manuscript) Across several panels (including "selected genes" dot plots and multiple parallel flow endpoints), it is not always clear what the unit of replication is (mice/samples vs cells), raising concern about pseudoreplication. Multiple-testing control is also not consistently described for families of related endpoints. Please clarify the unit of replication for each analysis, report mouse-level statistics, and either predefine primary endpoints or apply appropriate multiple-comparison correction within each figure/family.

We appreciate the reviewer for the helpful suggestion. We have made clarifications of appropriate multiple comparison correction, unit of replication and mouse-level statistics in our revised manuscript.

Minor points / clarifications

• For dot plots/selected-gene panels reporting t-test p-values: please specify the replicate unit, whether tests are performed at the mouse/sample level, and why FDR across the tested gene set (or broader universe) is not applied.

We thank the reviewer for highlighting this point. In our revised manuscript, we have clarified that for dot plot and selected-gene panels, expression values were first aggregated at the mouse level (mean expression per mouse per cluster), and statistical comparisons were performed using mouse-level averages as the unit of replication. Individual cells were not treated as independent replicates. Differential expression analyses at the single-cell level were performed using the Wilcoxon rank-sum test with Benjamini–Hochberg false discovery rate (FDR) correction. In Supplementary Table 2, we have included both representative differentially expressed genes (DGE) in the effector cluster and the complete DGE list.

• For functional readouts relying on PMA/ionomycin: consider clarifying that these measure maximal effector capacity and may not be optimal to distinguish PD-1 pathway effects that act via TCR/CD28-proximal signaling; TCR-dependent stimulation controls are more informative for the key mechanistic question.

We appreciate the reviewer for highlighting this point. We have made clarifications on using PMA and ionomycin for measuring T cell-intrinsic maximal effector capacity in our degranulation and intracellular cytokine staining for our $Pdcd1^{fl/fl}$ and Pdcd1 cKO mice in our revised manuscript.

• Where conclusions hinge on resident thymic populations, please provide quantitative summaries across mice of the extent of recirculant exclusion (not necessarily new experiments, but clearer reporting of the existing controls and their variability).

We thank the reviewer for this suggestion. In the revised manuscript, we provide mouse-level quantification of intravenous (i.v.) CD45 labeling to document the extent of recirculant exclusion within thymic SP $CD8^+$ T cells (**Extended Data Fig. 3A**, **Fig. 2A**). Across biological replicates, i.v. $CD45^+$ cells comprised only a mean of 0.0416% of thymic SP $CD8^+$ T cells in control mice (N=7) and 0.0566% in CD8-Pdcd1 cKO mice (N=5), demonstrating consistently minimal recirculant contribution. We additionally report the frequency and variability of $CD55^+H2\text{-}Kb^+$ subsets across mice to distinguish recirculating-like and resident compartments (**Fig. 2C-D**). In all panels where analyses are restricted to resident thymic populations, this exclusion is now explicitly stated in the figure legends. We have made clarifications in our revised manuscript.

Overall recommendation

The concept is strong and well aligned with the journal's scope. However, the two central mechanistic claims—functional cytotoxicity of thymic cells and the thymus–tumor clonotype linkage—still require more rigorous quantification and depth-aware statistical treatment to be fully convincing. The requested additions are targeted and should be achievable without expanding into many additional tumor models. Given that these points underpin the manuscript's main novelty, I do not recommend publication in its current form without addressing them.

We appreciated the overall comments of this reviewer, as above we have made point-to-point responses to all major and minor suggestions.
